# Genetic variants affect diurnal glucose levels throughout the day

**DOI:** 10.1038/s41467-026-72432-6

**Published:** 2026-05-22

**Authors:** Nasa Sinnott-Armstrong, Satu Strausz, Lea Urpa, Erik Abner, Josephine P. Johnson, Jesse Valliere, Teele Palumaa, FinnGen, Mark Daly, Samuel E. Jones, Hanna M. Ollila, Estonian Biobank Research Team, Erik Abner, Teele Palumaa, Priit Palta, VA Million Veteran Program, Josephine P. Johnson, Kyong-Mi Chang, Marijana Vujkovic, Priit Palta, Hassan S. Dashti, Kyong-Mi Chang, Marijana Vujkovic, Mark Daly, Jonathan K. Pritchard, Richa Saxena, Samuel E. Jones, Hanna M. Ollila

**Affiliations:** 1 Herbold Computational Biology Program, Fred Hutchinson Cancer Center, Seattle, WA USA; 2 Institute for Molecular Medicine Finland, FIMM, HiLIFE, University of Helsinki, Helsinki, Finland; 3 Department of Genome Sciences, University of Washington, Seattle, USA; 4 Brotman Baty Institute, University of Washington, Seattle, USA; 5 Department of Genetics, Stanford University, Stanford, CA USA; 6 Department of Oral and Maxillofacial Diseases, Helsinki University Hospital and University of Helsinki, Helsinki, Finland; 7 Department of Plastic Surgery, Cleft Palate and Craniofacial Center, Helsinki University Hospital and University of Helsinki, Helsinki, Finland; 8 Broad Institute of Harvard and MIT, Cambridge, MA USA; 9 Center for Genomic Medicine, Massachusetts General Hospital, Boston, MA USA; 10 Estonian Genome Center, Institute of Genomics, University of Tartu, Tartu, Estonia; 11 Corporal Michael J. Crescenz VA Medical Center, Philadelphia, PA USA; 12 Department of Medicine, University of Pennsylvania Perelman School of Medicine, Philadelphia, PA USA; 13 Department of Ophthalmology, Emory University, Atlanta, GA USA; 14 East Tallinn Central Hospital Eye Clinic, Tallinn, Estonia; 15 Department of Anesthesiology, Mass General Brigham, Harvard Medical School, Boston, MA USA; 16 Analytic and Translational Genetics Unit, Massachusetts General Hospital, Boston, MA USA; 17 Department of Biology, Stanford University, Stanford, CA USA; 18 Division of Sleep and Circadian Disorders, Brigham and Women’s Hospital, Harvard Medical School, Boston, MA USA

**Keywords:** Molecular medicine, Genetics, Genetic association study

## Abstract

Circadian rhythms not only coordinate the timing of wake and sleep but also regulate homeostasis within the body, including glucose metabolism. The genetic variants that contribute to the temporal control of glucose levels have not been previously examined. Using genome-wide data from ~420,000 individuals from the UK Biobank and replication in ~100,000 individuals from the Estonian Biobank, ~500,000 from FinnGen, ~160,000 from the VA Million Veteran Program, and ~52,000 from the MGB Biobank, we show that glucose levels are under diurnal genetic control. We discover a robust temporal association of glucose levels at the Melatonin receptor 1B (*MTNR1B*, rs10830963, *P* = 1×10^−22^) and a canonical circadian pacemaker gene Cryptochrome 2 (*CRY2)* loci (rs12419690, *P* = 1×10^−16^). Furthermore, we show that sleep modulates glucose levels, and the genetic variants have an independent role in diurnal glucose control. Finally, we show that these variants independently modulate risk of type 2 diabetes and that sleep medications including melatonin associate with type 2 diabetes. Our findings, together with earlier genetic and epidemiological evidence, show a clear connection between sleep and metabolism and highlight genetic variation at *MTNR1B* and *CRY2* in the control of diurnal glucose levels.

## Abstract (web summary)

Circadian rhythms influence metabolism, but genetic control of glucose timing was unknown. Here, the authors show that glucose levels are under diurnal genetic regulation, implicating variants in *MTNR1B* and *CRY2* that independently affect glucose rhythms and type 2 diabetes risk.

## Introduction

Circadian rhythms not only orchestrate the timing of wake and sleep but also regulate homeostasis within the body. Circadian rhythms are controlled by core circadian genes *CLOCK* and *BMAL1. CLOCK* and *BMAL* drive the expression of their repressors *PER* and *CRY* genes and create a ~24-h oscillation of gene activity^1^. This molecular clock is present in virtually all cells, with the central pacemaker located in the suprachiasmatic nucleus of the hypothalamus. The circadian system integrates external cues, such as light, temperature, feeding, and activity, to synchronize internal homeostasis and physiology with the environment. Circadian rhythms also significantly contribute to physiological processes, including diurnal variation in homeostasis and gene expression^2,3^. In addition to sleep-wake regulation, circadian rhythms contribute to the regulation of metabolism, affecting nutrient sensing, energy expenditure, glucose and lipid homeostasis, and hormone release. Furthermore, the core clock genes directly connect with metabolism. Glucose metabolism is affected by gluconeogenesis that is coordinated by the CRY genes. In addition, CLOCK and BMAL activate downstream effector genes like REV-ERBa directly at central and peripheral tissues, affecting the diurnal control of metabolism. Finally, disruption of circadian genes in model organisms manifests with altered glucose metabolism and diabetes^4^.

Melatonin, a molecule secreted at night from the pineal gland, is an important factor in regulating diurnal rhythms in humans. Its role extends from regulating circadian rhythms^5^ and circadian gene expression to influencing the timing and structure of sleep^6,7^. Moreover, melatonin is routinely measured in clinical settings for the diagnosis of suspected sleep-wake disorders. Additionally, it is commonly administered orally to adapt to time zones due to its phase-shifting properties and to treat insomnia due to its mild sedative effects.

Sleep and metabolism are tightly linked through diurnal rhythms^8^. Disrupted metabolism, exemplified by high glucose levels or clinical type-2 diabetes mellitus (T2DM),is associated with symptoms of insomnia, a late chronotype, and shortened sleep duration. These associations have been consistently observedin epidemiological cohort studies and are further supported by genetic analyses^9–12^. Nevertheless, the direct impact of genetic variation on diurnal variation in metabolism remains largely unclear.

Glucose levels fluctuate over the day and are affected by diet, physical activity, stress, sleep, and by hormonal factors that maintain physiologically safe levels in circulation^13^. Furthermore, the response to insulin, quantified as insulin sensitivity, is generally higher during the daytime and lower at night. Insulin secretion then typically peaks in the early morning, promoting glucose uptake by cells^14^. In contrast, glucagon, which raises blood glucose levels,is more active during periods of fasting, such as overnight. Moreover, during fasting periods, the liver releases glucose into the bloodstream and consequently, the liver plays a significant role in maintaining glucose homeostasis^13^.

A noteworthy aspect of T2DM and circulating glucose levels is their unique association with genetic variants from circadian rhythm genes in humans,notably melatonin receptor 1B (*MTNR1B*)^15–17^ and cryptochrome 2 (*CRY2*)^18^. Additionally, both sleep and glucose traits exhibit high heritability and robust genetic associations: glucose levels are associated with genes expressed in pancreatic tissue, adipose tissue and liver, and the associations have highlighted specific associations for different components of glucose, including fasting glucose^15–17^, random glucose^19^, T2DM^20,21^ or gestational diabetes^20^. Similarly, genome-wide association studies (GWAS) have identified core circadian genes and over 500 other variants associated with chronotype^22^, insomnia^23–25^, and sleep duration^26^.

A larger number of variants have been identified that contribute to biomarkers and metabolites at the normal range^27^. In addition, previous research conducted by our team and others has demonstrated that the disruption of circadian rhythms, such as through night shift work, escalates the risk of cardiovascular diseases and T2DM^28,29^. Furthermore, insomnia, evening chronotype, and short sleep are established independent risk factors for cardiometabolic diseases. The strength of this connection is further underscored by studies on cardiometabolic traits themselves, revealing that two of the largest-effect genetic variants associated with elevated glucose levels and T2DM are located within regulatory regions of *MTNR1B*^15–18^. Previous investigations into *MTNR1B* have established that the risk variant rs10830963 influences insulin secretion and glucose levels^17^. Functional studies on pancreatic islets have revealed that the alternative allele of *MTNR1B* exerts an inhibitory effect on melatonin response, leading to reduced insulin secretion, increased fasting glucose levels, and a heightened risk of T2DM^30^.

These compelling earlier findings prompt the question of whether genetic variants are linked to the diurnal control of glucose levels. In this study, we present evidence using data from 420,000 individuals from the UK Biobank demonstrating that the genetic variants at *MTNR1B* and *CRY2* modulate the diurnal pattern of glucose levels.

## Results

### Glucose levels show time-dependent associations with *CRY2* and *MTNR1B*

**Fig. 1:**
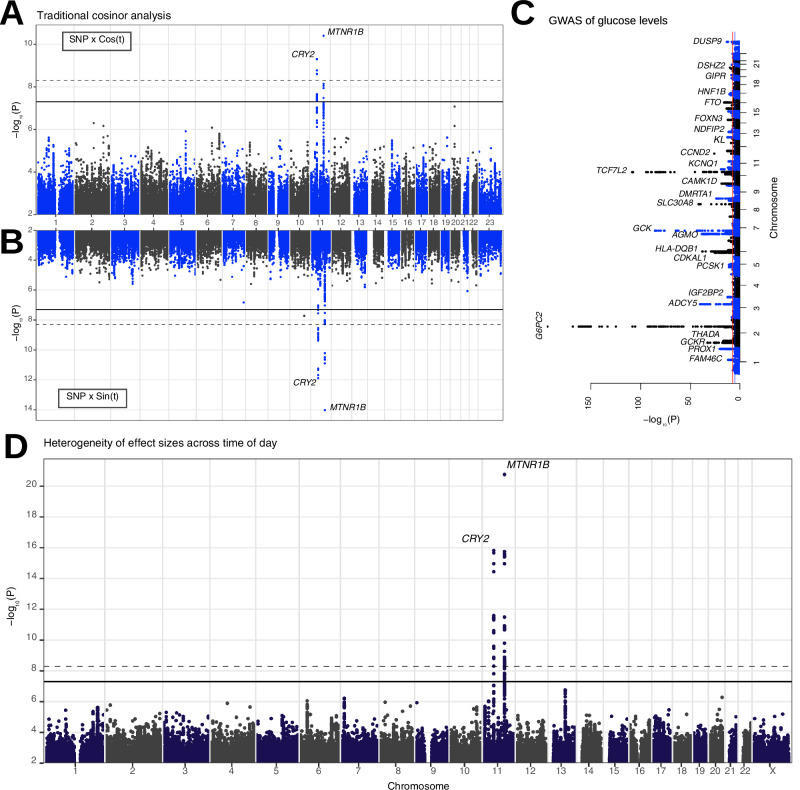
Diurnal association of MTNR1B and CRY2 loci with glucose levels. **A**, **B** Interaction after accounting for variant main effects shows genome-wide significant interactions over the day at MTNR1B and CRY2 loci in the classical circadian cosinor model. *P*-value represents the test for interaction of variant x cosine(time) (**A**) or variant x sine (**B**) (lower panel) from a regenie interaction model. **C** Manhattan plot of GWAS of glucose levels in UKB shows 66 independent associations. Most significant 25 loci and HLA lead signals are annotated to the closest gene. **D** Estimating heterogeneity of meta-analysis of glucose levels by each hour of the day shows significant heterogeneity at the MTNR1B and CRY2 loci. *P*-values represent the test for significant heterogeneity in effect size among GWASes performed in nonoverlapping 1 h bins over the day. In **A**, **B**, **D** the solid line represents the genome-wide significant threshold *P* = 5e-8, and the dashed line represents the *P* value corrected for multiple testing and the number of traits or bins in each analysis.

While earlier GWAS have revealed a substantial number of variants that contribute to both random and fasting glucose levels^15–17,20,21^, it is possible that the effect of genetic variants may also be time-dependent. To test a possible diurnal effect from genetic variants on glucose temporality, we performed cosinor analysis in glucose measurements and time of sample collection in the UK Biobank. Cosinor analysis is a traditional method for examining circadian or diurnal effects that assumes effects are cyclical and time-dependent. We fit sine and cosine models by assuming the time at midnight as time zero. The genome-wide cosinor analysis identified two loci, *MTNR1B* (rs10830963, *P* < 5e-8) and *CRY2* (rs12419690, *P* < 5e-8, Fig. 1A, B).

Curiously, while we discovered 66 genome-wide significant associations with normal glucose levels (Fig. 1C and Supplementary Data 1), there was no association at either *MTNR1B* or *CRY2* when adjusted for time of day of glucose draw (*MTNR1B* *P* = 0.27, *CRY2* rs12419690 *P* = 0.46 Supplementary Figs. 1 and 2). Thus, we hypothesized that time-dependent effects would also manifest as heterogeneity in association strength throughout the day. This is particularly important as cosinor models rarely fit or perfectly reflect the time variation of glucose levels in a population as a result of the daily fluctuation that is modified by meals and hormonal balance. In addition, most measurements obtained in most cohorts are collected during daytime hours. Using the UK Biobank, we performed GWAS of glucose levels in 1-h nonoverlapping intervals using measurement times from 9:00 am to 7:00 pm. Indeed, we detected high heterogeneity of effect sizes only at the *MTNR1B* (rs10830963, *P*(*I*^2^) = 1 × 10^−22^) and *CRY2* loci (rs12419690, *P*(*I*^2^) = 1 × 10^−16^, Fig. 1D).

### Diurnal variants have reversed associations in the morning versus evening

**Fig. 2:**
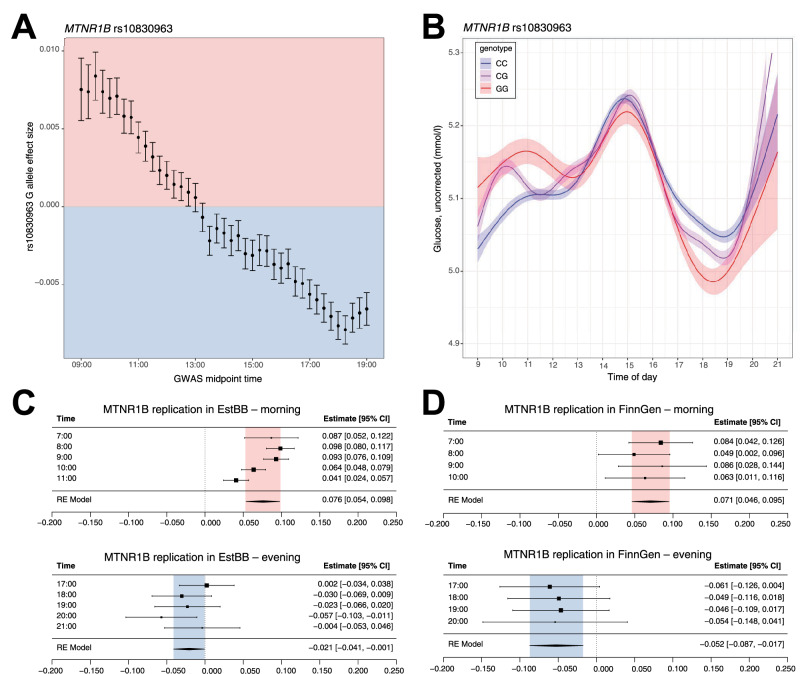
Effect sizes at the *MTNR1B* locus are heterogenous over the day. Effect sizes at the *MTNR1B* locus (**A**) are heterogeneous over the day from 7 AM to 7 PM, showing a decrease in effect that is flipped towards the afternoon and evening, with data presented as effect size and 95% confidence interval. Sample sizes for each time point are reported in the “Methods” section. **B** Analysis using generalized linear model (GAM) spline over the day with unadjusted glucose values shows a consistent and higher effect of risk allele rs10830963G in the morning than in the evening, with data presented as GAM-predicted values across the day with standard error ribbon. The association of the higher effect of risk allele rs10830963G is replicated in both the Estonian Biobank (*n*_morning_ = 110,739, *n*_evening_ = 20,149) (**C**) and in FinnGen (*n*_morning_ = 49,555, *n*_evening_ = 30,063) (**D**) in both the morning and in the evening, with data presented as effect size and 95% confidence interval. In **A**, **C**, **D** red shading highlights morning positive effect sizes, while blue shading highlights negative evening effect sizes.

Heterogeneity statistics, while useful for omnibus analysis of variation throughout the day, do not help us understand overall trends between associated time bins. Thus, we visualized the effect sizes used in the heterogeneity analysis to examine overall trends. For *MTNR1B*, the effect size for the allele associated with higher glucose levels in the morning showed association with lower glucose levels in the afternoon and evening, supporting a consistent diurnal genetic association with glucose levels (Fig. 2A). A similar trend was present for *CRY2* (Supplementary Fig. 3). Our finding of time-dependent genetic associations also suggests the possibility that trait heritability could vary over time of day. When we utilized these genome-wide association results throughout the day to examine heritability, we observed a change over the day. This suggests that time of day can affect the genetic contribution to trait heritability, in addition to single variant effects across the day (Supplementary Fig. 4).

Moreover, to formally test the time-dependent effect, we fit a spline and tested diurnal effects by genotype using a generalized additive model. We discovered, analogous to the effect size heterogeneity analysis, that risk increasing alleles of *MTNR1B* showed strong intra-day variation in levels, with maximum effect size that was reversed from the morning towards the evening (*P* = 1 × 10^−6^, Fig. 2B). These findings indicated that genetic variants could show opposite association direction on a phenotype, such as glucose depending on time of day when the phenotype was measured. Based on these analyses, we estimate that homozygous carriers of the risk alleles have 0.05 mmol/l (*MTNR1B)* and 0.035 mmol/l (*CRY2*) higher glucose levels in the morning.

### Association of *MTNR1B* and *CRY2* with diurnal glucose levels replicates in independent cohorts and with fasting glucose levels

Glucose levels are heavily influenced by environmental factors, most notably dietary intake. We therefore tested the total variance explained by different factors, including time of day, for glucose levels and compared it to other clinical biomarkers measured in the UK Biobank. Ten percent of variation in both fasting and non-fasting levels was explained by time of day, the highest variance explained by time of day across all measured biomarkers^27^.

Given the novel methodology we use in this study, we wanted to replicate our findings in independent cohorts. We therefore tested the associations in the Estonian Biobank (EstBB) in 100,000 individuals, in FinnGen in 500,000 individuals, in the Mass General Brigham Biobank (MGBB) in 52,000 individuals, and in the VA Million Veteran Program (MVP) among 115,096 individuals of European, 31,299 individuals of African American, and 14,680 individuals of Hispanic ancestry. Unlike in the UK Biobank, in these replication cohorts, the corresponding laboratory measures are drawn from patient electronic health records.

The heterogeneity of effect sizes across the day at *MTNR1B* (rs10830963) and CRY2 was significant in all replication cohorts (rs10830963, EstBB *P* = 8.2 × 10^−28^, FinnGen *P* = 2.6 × 10^−8^, MGB Biobank *p* = 1.6 × 10^−4^, MVP EUR *p* = 3.2 × 10^−12^, Supplementary Figs. 5 and 6). Furthermore, the *MTNR1B* (rs10830963) morning effect replicated in Mass General Brigham Biobank (*P* = 1.4 × 10^−8^), and MVP European (*p* = 1.2 × 10^−4^) and Hispanic (*p* = 2.08e-4) populations in the morning (Supplementary Fig. 7). In addition, we observed the opposite associations from morning to evening in the Estonian Biobank (morning *p* = 1.6 × 10^−11^, evening *p* = 0.0437, Fig. 2C and Supplementary Fig. 9) and FinnGen (morning *P* = 1.2 × 10^−8^, evening *p* = 0.003, Fig. 2D and Supplementary Fig. 9) for *MTNR1B* (rs10830963) although the evening time points had substantially fewer samples in all the EHR based cohorts and subsequently lower power.

Utilizing the large number of glucose measures available in FinnGen, we were also able to find a set of individuals who had blood glucose measurements in both the morning (7:00–11:00 am) and evening (5:00–9:00 pm) (*n* = 22,456). We then calculated the difference between morning and evening glucose measures and found that both risk variants were associated with the magnitude of diurnal glucose difference (*MTNR1B* rs10830963 beta = −0.087, *P* = 2.79 × 10^−3^, *CRY2* rs12419690 beta = 0.055, *P* = 0.046). Overall, the results support a robust association between particularly *MTNR1B* rs10830963 with diurnal glucose levels, with opposite effects in the morning versus evening, and across independent cohorts.

**Fig. 3:**
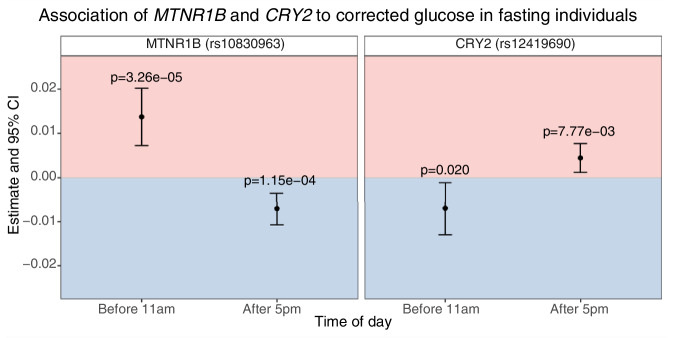
Association of *MTNR1B* and *CRY2* with glucose levels measured in individuals at fasting for at least 6 h in the UK Biobank. Association data for  *MTNR1B* rs10830963 (left), and for *CRY2* rs12419690 (right). The panels show association with glucose levels in individuals where sample collection individuals in the morning samples (prior to 11 AM, *n* = 6046) or in the evening samples (after 5 PM, *n* = 14,825). Data are presented as effect size and 95% confidence intervals of a linear regression of adjusted glucose with risk genotype at each time point, adjusted by sex and other relevant covariates (see “Methods”). Red shading highlights morning positive effect sizes, while blue shading highlights negative evening effect sizes.

As UKB is primarily a non-fasting population, it is possible that the temporal association of *MTNR1B* and *CRY2* with glucose levels would be mediated by eating. To test the possible effect of fasting, we examined the subset of UKB that reported fasting for at least 6, 8, or 12 h at the time of sample collection (Supplementary Fig. 10 and Supplementary Table 1). Due to substantially smaller sample size of fasting samples (*N* = 14,000 individuals with 8 h or longer fasting), we stratified the analysis by sample collected before 11 AM vs. sample collected after 5 PM in each fasting category and computed association statistics within the groups (Supplementary Fig. 3). Similar to earlier analysis in the full sample, the effect was in the opposite direction after 5 PM (*P* = 0.001, Fig. 3A). Similarly, *CRY2* rs12419690 A allele associated with lower morning glucose levels (*P* = 0.03) but with higher evening glucose levels (*P* = 0.001, Fig. 3B, and in fasting categories Supplementary Figs. 11 and 12). We observe an association of *MTNR1B* and *CRY2* in all the fasting categories in which we were sufficiently powered, where the *MTNR1B* rs10830963 risk allele was associated with higher morning glucose levels (*P* = 6.9 × 10^−5^).

Variants in *MTNR1B* have also been previously associated with elevated hemoglobin A1c (HbA1c), or glycosylated hemoglobin, which represents blood glucose levels over the previous 2–3 months^31^. We examined the effect of the *MTNR1B* rs10840963 variant on HbA1c levels in the UK Biobank sample, and found that while there is an effect of the allele on HbA1c, this effect was consistent throughout the day (Supplementary Fig. 13).

### Glucose regulation is connected with sleep and circadian traits

**Fig. 4:**
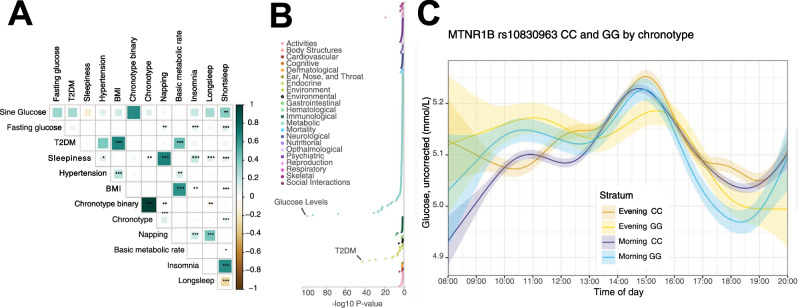
Relationship between sleep and glucose traits. **A** Genetic correlation analysis between sleep and circadian traits, and metabolic traits. **B** PheWAS of *MTNR1B* rs10830963 variant shows association with glucose levels and type-2 diabetes mellitus. **C** Glucose levels in individuals with morning versus evening chronotype for the *MTNR1B* rs10830963 variant show consistent effect size independent of chronotype, with data presented as values across the day predicted by GAM model with standard error ribbon.

Epidemiological studies and individual genetic associations suggest a tight connection between sleep and glucose traits. Therefore, we tested for a possible shared genetic architecture between glucose and sleep traits using genetic correlation analysis. We observed genetic correlation of fasting glucose levels with napping, insomnia and short sleep, which further connect sleep and circadian traits with glucose homeostasis (Fig. 4A). Furthermore, other metabolic traits, such as BMI, associated with insomnia and sleepiness, agreeing with earlier literature^32^.

We further hypothesized that circadian rhythm coordinates both morningness-eveningness (as measured by chronotype) and diurnal glucose levels. Furthermore, to clarify the phenotypic associations with *MTNR1B* (rs10830963), we performed single-variant-pheWAS, which showed association primarily with metabolic traits and surprisingly not with sleep traits (Fig. 4B). We therefore stratified diurnal glucose levels by chronotype and found that MTNR1B risk allele carriers had higher morning glucose levels independently of chronotype (Fig. 4C). To further understand this finding, we conducted a gene-level-pheWAS of *MTNR1B* and *CRY2*. Our additional analysis revealed independent SNPs at both loci that significantly associate with chronotype but are not in LD (*r*^2^ < 0.3) with the corresponding diurnal glucose-associated variants (Supplementary Fig. 14). Therefore, these two loci are known to have multiple regulators, and our analysis suggests that these regulators might independently act in the context of both circadian and diurnal control.

### Daytime sleepiness and napping disrupt the association of *MTNR1B* and *CRY2* with diurnal glucose levels

Existing literature suggests that sleep disturbance can contribute to higher glucose levels and decreased insulin sensitivity^33^. We therefore tested if chronotype, sleep duration, insomnia, ease of awakening, daytime sleeping or daytime napping modulated the association between diurnal glucose and both the *MTNR1B* and *CRY2* alleles. We observed that *MTNR1B* and *CRY2* variants showed a robust temporal association in individuals who reported insomnia, late chronotype, or short sleep (Supplementary Figs. 15–17). In contrast, individuals who reported daytime napping or sleepiness showed no association between *MTNR1B* or *CRY2* with glucose levels (Supplementary Fig. 18).

In addition, all times of day, we observed a significant epidemiological association between higher glucose levels and insomnia, daytime napping, and daytime sleep (Supplementary Figs. 19–21). We also found a significant association between longer sleep and increased glucose levels (Supplementary Fig. 22) and ease of awakening and lower glucose levels (Supplementary Fig. 23), but only when glucose was measured in the evening. However, we observed no significant interaction between *MTNR1B* and *CRY2* glucose risk variants and any sleep phenotypes (Supplementary Figs. 19–23), suggesting an independent role of the genetic associations and sleep traits on diurnal glucose variation.

### Melatonin supplementation is related to higher incidence of type-2 diabetes mellitus

**Fig. 5:**
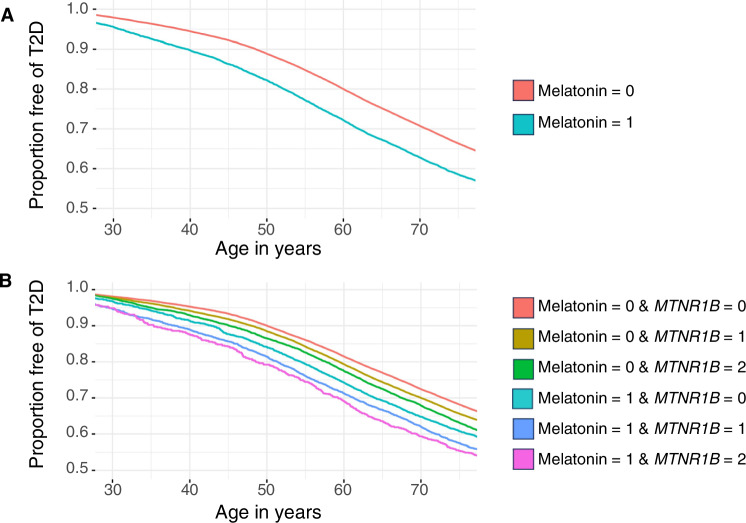
Incidence of T2DM and melatonin prescription. **A** Incidence of T2DM in individuals without (red) or with (blue) melatonin prescription shows earlier T2DM onset in individuals with melatonin usage. **B** Incidence of T2DM in individuals with or without melatonin prescription and by MTNR1B risk allele carrier status shows the highest incidence in individuals with melatonin prescription and MTNR1B risk allele.

Melatonin can influence glucose levels in two ways. First, indirectly through related sleep traits as described above. Second, by directly modulating insulin secretion and insulin sensitivity as suggested by earlier functional studies^34^. Furthermore, the direct effect on insulin sensitivity has been identified in short-term melatonin supplementation studies^30^. To understand if melatonin supplementation might have a long-term effect on metabolic health, we examined the incidence of T2DM in individuals with melatonin prescription and the identified risk variants in FinnGen. We observed an association between melatonin prescriptions and higher risk for T2DM (HR = 1.23, 95%CI = 1.18–1.28, *P* = 2.56 × 10^−21^ Fig. 5A). Furthermore, individuals with melatonin prescription had an earlier age of onset compared to controls (HR = 1.23, 95%CI = 1.18–1.28, *P* = 2.56 × 10^−21^). As expected, the *MTNR1B* risk allele is also associated with incident T2DM (rs10830963, HR = 1.08, 95%CI = 1.06–1.10, *P* = 2.31 × 10^−21^, Fig. 5B).

Additionally, we calculated whether we observe a similar effect for non-benzodiazepine medications commonly used for the treatment of insomnia (z-drugs). We discovered an increased risk between z-drugs and T2DM (HR = 1.21, 95% CI = 1.17–1.26, *P* = 6.10 × 10^−24^). In addition, adding the *MTNR1B* risk allele to the model, it was significantly associated with the incidence of T2DM (rs10830963, HR = 1.21, 95% CI = 1.17–1.26, *P* = 8.53 × 10^−13^), suggesting that sleep problems and medications, including melatonin and z-drugs, associate with T2DM.

## Discussion

Here, we show that genetic variants at two canonical sleep and circadian genes, *MTNR1B* and *CRY2*, are associated with time-dependent glucose levels in humans. In addition, the association was in opposite directions in the morning versus evening, connecting regulation of homeostasis across the day with the regulation of glucose metabolism and with specific genetic loci. The findings indicate that the timing of glucose levels is under genetic control. Further, the same genes that contribute to diurnal variation modulate pathology, such as T2DM.

MTNR1B is a G protein-coupled receptor (GPCR). Overall, GPCRs constitute the largest class of membrane receptors^35^ and the central role of GPCRs in human biology is highlighted by the fact that 36% of FDA-approved drugs act on GPCRs^36^. Such effects are also accentuated with MTNR1B, including the effects in the central nervous system in sleep and circadian rhythms^37^, role in glucose metabolism in the pancreas^38^ and effects on temperature regulation and cardiovascular system through vasodilation^39^. This underscores both the clinical importance and the vast biological and clinical effects of GPCRs, including the membrane-bound GPCR MTNR1B, which is already used as a pharmaceutical target.

One key finding in this paper was the robustness of temporal effects. We observed the association between genetic variants and the timing of glucose levels across three methods. A canonical cosinor model tests a sine and cosine of glucose levels by genotype and showed a genome-wide significant association with *MTNR1B* and *CRY2*. We employ multiple nonadditive statistical approaches—including classic cosinor analysis from the sleep literature, meta-analysis heterogeneity tests, and generalized additive models—to assess the environmental contribution of diurnal variation to genetic effects. This approach robustly identified two known regulators of circadian rhythm. By integrating alternative modeling strategies into classical GWAS studies, we uncover previously unknown biology and can identify additional traits of interest. Applying models, which include time-varying components, is critical to a comprehensive survey of the genetic architecture of complex traits. Furthermore, it informs how biological time and diurnal rhythms act in context to influence complex traits. Moreover, the benefit of the methods is first the feasibility of applying a cosinor model in GWAS, whereas GAM and heterogeneity analyses can be used in data with even more variable temporal structure, as they do not assume a perfect cyclical pattern across the day. These approaches can be used for other time-varying data as well, such as circannual variation.

Earlier GWAS on glucose levels have typically discovered *MTNR1B* and *CRY2* associations with fasting glucose levels^16–19^. Moreover, *MTNR1B* and *CRY2* associate with T2DM^16,17^, implicating their role in clinically relevant diseases. Our analysis of fasting samples suggests that one of the primary contributing factors for the association most likely is the time component and not the fasting status alone. Consequently, including time-dependent analysis in GWAS has the potential with larger data sets also, to clarify biology, bringing additional temporal components into the regulation of homeostasis of metabolites.

It is also intriguing to disentangle the possible mechanisms of how *MTNR1B* and *CRY2* variation modulate glucose levels. Earlier literature on melatonin suggests that even short-term melatonin supplementation can induce insulin resistance^30^. We also show in this paper that *MTNR1B* and *CRY2* modulated expression levels to opposite directions in the morning versus in the evening. Melatonin typically indicates biological night, whereas the effects of *CRY2* are potentially related to clock time versus biological time. Imbalance in clock time and biological time is pronounced in shift work or in jet lag—both of which also induce cardiometabolic changes and diseases. However, the role of melatonin in glucose metabolism has been shown either beneficial or harmful in earlier studies. While melatonin supplementation may increase diabetes risk or insulin insensitivity^30^, functional models have also shown that melatonin enhances insulin secretion in pancreatic beta cells^34^. Our findings align with both these observations, where melatonin supplementation associates with higher T2DM incidence. However, in our data individuals taking also other sleep medications (z-drugs), have a higher incidence of T2DM supporting an overall association between sleep medication and T2DM. Furthermore, individuals with *MTNR1B* or *CRY2* risk variants also have lower glucose levels in the afternoon.

Furthermore, we observe this association independent of insomnia, chronotype or sleep duration. However, sleep problems measured as insomnia or late chronotype had an additional, independent negative effect on glucose levels. Moreover, *CRY2* and *MTNR1B* did not show interaction with sleep traits on glucose levels. First, these findings implicate that sleep has an independent modulatory effect on glucose. Second, genetic variation, such as variation at *CRY2* and *MTNR1B,* has an independent and additional role of modulating glucose levels in a time-dependent fashion.

Our findings should be interpreted in the light of the following limitations. While the cohorts have extensive medical information, the available data types, such glucose-lowering medications or diabetes treatment was not available at similar resolution at all cohorts. This may influence the observed associations in the study. Furthermore, the current study uses population data with different individuals at different time points. Therefore, we cannot directly assess individual glucose rhythm differences between individuals, except in the context of genetic variation. Finally, prescription data does not capture compliance with medication usage, which may reduce power in observing associations in the current study.

Overall, the findings indicate that homeostatic glucose levels are modulated by environmental factors, such as sleep, as shown here and in previous work. Moreover, inherent genetic factors control the magnitude of overall fasting glucose levels. Finally, genetic factors modulate the timing of glucose levels as well. This is likely true in other settings known to regulate these genes, such as the regulation of gestational diabetes in pregnancy by *MTNR1B* and applying time-varying models in these unique biological settings will likely reveal additional mechanisms of environment-dependent regulation. Our findings underscore the diurnal control exerted by *MTNR1B* and *CRY2* on glucose homeostasis, shedding light on the intricate interplay between diurnal rhythms, biological time, metabolism, and fundamental physiological processes.

## Methods

### Cohorts

The UK Biobank is a long-term ongoing study investigating genetic and environmental risk factors that lead to disease. The study contains data on a UK population-based cohort of 503,325 participants aged between 40 and 70. Recruited between 2006 and 2010, participants were invited by means of an invitation letter (UK Biobank Resource 100253) to attend an appointment at one of 22 recruitment centers across the UK. At this baseline visit, participants were asked a series of questions about a number of sociodemographic factors, their lifestyle and their medical history^40^. In addition, physical measurements were taken, cognitive tests performed, and biological samples provided (blood, urine and saliva). The 503,325 consenting participants were recruited from ~9.2 million individuals initially invited: a response rate of 5.47%^41^. Due to ascertainment bias, UK Biobank participants were more likely to be of higher socioeconomic status (SES) and healthier than the population average in the UK^42^.

The Estonian Biobank is a population-based biobank with 212,955 participants in the current data freeze (2023v1). All biobank participants have signed a broad informed consent form, and their EHR information, including ICD-10 codes, is obtained via regular linking with the national health insurance fund and other relevant databases, with the majority of the electronic health records having been collected since 2004^43^.

The Mass General Brigham (MGB) Biobank is a healthcare enterprise clinical cohort with 64,639 patients from the MGB healthcare network in Massachusetts, USA, that links health records with genetic and lifestyle data since 2009. Written informed consent was obtained from all patients upon enrollment. The present study protocol was approved by the MGB Institutional Review Board (#2018P002276).

FinnGen is a public-private research project launched in 2017 that combines digital healthcare data and genome data on approximately 500,000 Finns and aims to provide novel insight into human disease. All study subjects provided informed consent for biobank research, based on the Finnish Biobank Act, or consented to project-specific consents before the Finnish Biobank Act came into consent (September 2013). The Coordinating Ethics Committee of the Hospital District of Helsinki and Uusimaa (HUS) statement number for the FinnGen study is Nr HUS/990/2017.

The MVP is a mega-biobank of veterans who accessed healthcare in the Department of Veterans Affairs (VA) healthcare system, which comprises more than 1200 hospitals, medical centers, and community outpatient clinics nationwide^44^. All care is recorded in a central data repository and includes demographics, outpatient and inpatient encounters, diagnoses, pharmacy dispensing records, vital signs, laboratory measures, and death information. The MVP received approval from the Central Veterans Affairs Institutional Review Board (IRB) and site-specific IRBs. All MVP study participants provided written informed consent, a blood sample for genotyping, and allowed access to their EHR for research purposes. Subjects were recruited from VA Medical Centers participating in the Million Veteran Program. Veterans were identified from VA databases and recruited via invitational and appointment mailings. In addition, Veterans were recruited at selected VA Medical Centers. More details on recruitment are previously provided^44^.

The design and conduct for this study complies with all relevant regulations regarding the use of human study participants and was conducted in accordance with the criteria set by the Declaration of Helsinki.

### Ethical statements

#### Ethics statement Estonian Biobank

The activities of the EstBB are regulated by the Human Genes Research Act, which was adopted in 2000 specifically for the operations of EstBB. Individual-level data analysis in EstBB was carried out under ethical approval 1.1-12/624 from the Estonian Committee on Bioethics and Human Research (Estonian Ministry of Social Affairs), using data according to release application 6-7/GI/10108 from the Estonian Biobank.

#### Ethics statement UK Biobank

The UK Biobank has received approval as a Research Tissue Bank from the North West Multi-center Research Ethics Committee (MREC) under MREC permits 11/NW/0382 (2011–2016), 16/NW/0274 (2016–2021), and 21/NW/0157 (2021–2026). Researchers with approved applications are covered by these permits and are not required to seek additional approval, except in specific cases (see section B7 of the UK Biobank Access Procedures document: https://www.ukbiobank.ac.uk/media/omtl1ie4/access-procedures-2011-1.pdf). All participants of the UK Biobank study provided consent, at the baseline visit, for their personal data and biological samples to be collected and stored for research purposes. Participants are given the option to withdraw their consent at any time; any samples that have withdrawn their consent at the time of analysis were excluded from this study. A print version of the electronic consent form is stored as UK Biobank Resource 100252.

#### Ethics statement FinnGen

Study subjects in FinnGen provided informed consent for biobank research, based on the Finnish Biobank Act. Alternatively, separate research cohorts, collected prior the Finnish Biobank Act came into effect (in September 2013) and the start of FinnGen (August 2017), were collected based on study-specific consents and later transferred to the Finnish biobanks after approval by Fimea (Finnish Medicines Agency), the National Supervisory Authority for Welfare and Health. Recruitment protocols followed the biobank protocols approved by Fimea. The Coordinating Ethics Committee of the Hospital District of Helsinki and Uusimaa (HUS) statement number for the FinnGen study is Nr HUS/990/2017.

The FinnGen study is approved by Finnish Institute for Health and Welfare (permit numbers: THL/2031/6.02.00/2017, THL/1101/5.05.00/2017, THL/341/6.02.00/2018, THL/2222/6.02.00/2018, THL/283/6.02.00/2019, THL/1721/5.05.00/2019 and THL/1524/5.05.00/2020), digital and population data service agency (permit numbers: VRK43431/2017-3, VRK/6909/2018-3, VRK/4415/2019-3), the Social Insurance Institution (permit numbers: KELA 58/522/2017, KELA 131/522/2018, KELA 70/522/2019, KELA 98/522/2019, KELA 134/522/2019, KELA 138/522/2019, KELA 2/522/2020, KELA 16/522/2020), Findata permit numbers THL/2364/14.02/2020, THL/4055/14.06.00/2020, THL/3433/14.06.00/2020, THL/4432/14.06/2020, THL/5189/14.06/2020, THL/5894/14.06.00/2020, THL/6619/14.06.00/2020, THL/209/14.06.00/2021, THL/688/14.06.00/2021, THL/1284/14.06.00/2021, THL/1965/14.06.00/2021, THL/5546/14.02.00/2020, THL/2658/14.06.00/2021, THL/4235/14.06.00/2021, Statistics Finland (permit numbers: TK-53-1041-17 and TK/143/07.03.00/2020 (earlier TK-53-90-20) TK/1735/07.03.00/2021, TK/3112/07.03.00/2021) and Finnish Registry for Kidney Diseases permission/extract from the meeting minutes on 4th July 2019.

The Biobank Access Decisions for FinnGen samples and data utilized in FinnGen Data Freeze 12 include: THL Biobank BB2017_55, BB2017_111, BB2018_19, BB_2018_34, BB_2018_67, BB2018_71, BB2019_7, BB2019_8, BB2019_26, BB2020_1, BB2021_65, Finnish Red Cross Blood Service Biobank 7.12.2017, Helsinki Biobank HUS/359/2017, HUS/248/2020, HUS/430/2021 §28, §29, HUS/150/2022 §12, §13, §14, §15, §16, §17, §18, §23, §58, §59, HUS/128/2023 §18, Auria Biobank AB17-5154 and amendment #1 (August 17 2020) and amendments BB_2021-0140, BB_2021-0156 (August 26 2021, Feb 2 2022), BB_2021-0169, BB_2021-0179, BB_2021-0161, AB20-5926 and amendment #1 (April 23 2020) and it´s modifications (September 22 2021), BB_2022-0262, BB_2022-0256, Biobank Borealis of Northern Finland_2017_1013, 2021_5010, 2021_5010 amendment, 2021_5018, 2021_5018 amendment, 2021_5015, 2021_5015 amendment, 2021_5015 amendment_2, 2021_5023, 2021_5023 amendment, 2021_5023 amendment_2, 2021_5017, 2021_5017 amendment, 2022_6001, 2022_6001 amendment, 2022_6006 amendment, 2022_6006 amendment, 2022_6006 amendment_2, BB22-0067, 2022_0262, 2022_0262 amendment, Biobank of Eastern Finland 1186/2018 and amendment 22§/2020, 53§/2021, 13§/2022, 14§/2022, 15§/2022, 27§/2022, 28§/2022, 29§/2022, 33§/2022, 35§/2022, 36§/2022, 37§/2022, 39§/2022, 7§/2023, 32§/2023, 33§/2023, 34§/2023, 35§/2023, 36§/2023, 37§/2023, 38§/2023, 39§/2023, 40§/2023, 41§/2023, Finnish Clinical Biobank Tampere MH0004 and amendments (21.02.2020 and 06.10.2020), BB2021-0140 8§/2021, 9§/2021, §9/2022, §10/2022, §12/2022, 13§/2022, §20/2022, §21/2022, §22/2022, §23/2022, 28§/2022, 29§/2022, 30§/2022, 31§/2022, 32§/2022, 38§/2022, 40§/2022, 42§/2022, 1§/2023, Central Finland Biobank 1-2017, BB_2021-0161, BB_2021-0169, BB_2021-0179, BB_2021-0170, BB_2022-0256, BB_2022-0262, BB22-0067, decision allowing to continue data processing until 31st August 2024 for projects: BB_2021-0179, BB22-0067,BB_2022-0262, BB_2021-0170, BB_2021-0164, BB_2021-0161, and BB_2021-0169, and Terveystalo Biobank STB 2018001 and amendment 25th August 2020, Finnish Hematological Registry and Clinical Biobank decision 18th June 2021, Arctic biobank P0844: ARC_2021_1001.

#### Ethics statement MGBB

The MGB Biobank has obtained a Certificate of Confidentiality. In addition, the MGB Biobank works in close collaboration with the Partners Human Research Committee (PHRC) (the Institutional Review Board). This collaboration has ensured that the Biobank’s actions and procedures meet the ethical standards for research with human subjects. Biobank patients are recruited from inpatient stays, emergency department settings, outpatient visits, and electronically through a secure online portal for patients. Recruitment and consent materials are fully translated in Spanish to promote patient inclusion. The systematic enrollment of patients across the MGB network and the active inclusion of patients from diverse backgrounds contribute to a Biobank reflective of the overall demographic of the population receiving care within the MGB network. Recruitment for the Biobank launched in 2009 and is ongoing through both in-person recruitment at participating clinics and electronically through the patient portal. The recruitment strategy has been described previously^45^. All recruited patients provided written consent upon enrollment, and are offered an option to refuse consent.

#### Ethics statement MVP

The VA Office of Research and Development Central IRB approved the MVP003 and MVP028 study protocols. For all other studies with summary statistic level data, the participants provided written informed consent in IRB- approved protocols.

### Blood and urine sampling

Participants recruited into the UK Biobank study consented to providing a blood sample at their initial (baseline) visit, and approximately 480,000 participants gave ~45 ml of blood at the assessment center. Samples were not asked to fast prior to their visit, but self-report fasting time (data field 74) was recorded. Each participant’s sample tube contained a unique barcode, which was scanned on the computerized system, typically a few seconds after sample collection. The timestamp of this barcode scan (data field 3166) was used to determine time of day and date of sample collection, used for deriving the phenotypes and covariates (see below). Part of the blood sample was collected in a serum separator tube, which was then left to clot for 30 min at room temperature and then refrigerated before being transported to the central UK Biobank processing facility. Once separated out, the serum was frozen at −80 °C for later processing (see UK Biobank Resource 5636; https://biobank.ctsu.ox.ac.uk/crystal/crystal/docs/biomarker_issues.pdf) for 29 serum-based markers.

### Glucose measurements

Blood glucose (mmol/L; data field 30740) was measured (along with other blood biochemistry markers) using the serum samples that were processed at a later date (data field 30741) using the Beckman Coulter AU5800 platform (see resource 1227; https://biobank.ctsu.ox.ac.uk/crystal/crystal/docs/serum_biochemistry.pdf), and extensive quality control and validation was performed to minimize sources of systematic and random variation^46^. We used glucose data from the initial visit only. For the replication cohorts FinnGen, Estonian Biobank and MGB Biobank, we obtained glucose data from the electronic health record data.

### Genotype data

#### UK Biobank

DNA extracted from blood samples provided by UK Biobank participants was genotyped in 106 batches of approximately 4700 samples per batch. The first 11 batches (*N* = 49,950) were genotyped using a custom Affymetrix genotyping array referred to as the UK BiLEVE^47^. The remaining samples (*N* = 438,427) were genotyped using the very similar (95% overlap) UK Biobank Axiom array, also by Affymetrix (now part of Thermo Fisher Scientific). The arrays contained up to 850,000 probes with ~650,000 variants used as a dense scaffold for imputation of uncaptured variants, ~125,000 probes for rare and coding variants, ~47,000 probes within so-called regions of interest and ~45,000 markers previously linked to specific phenotypes. Further details of the contents of these arrays can be found at http://tools.thermofisher.com/content/sfs/brochures/uk_axiom_biobank_contentsummary_brochure.pdf. For imputation, markers were included if they had <5% missingness and minor allele frequency (MAF) > 0.0001, and all samples were included except for those with excessive heterozygosity and high missingness. Genotypes were phased with SHAPEIT3 (with 1000 Genomes as a reference panel) and then imputed using both the Haplotype Reference Consortium reference panel^48^ and a combined panel of UK10K and 1000 Genomes phase 3^49^. The two imputed datasets were combined to provide approximately 93 million autosomal variants and nearly 4 million X chromosome variants^50^.

#### Estonian Biobank

All EstBB participants have been genotyped at the Core Genotyping Lab of the Institute of Genomics, University of Tartu, using Illumina Global Screening Array v3.0_EST. Samples were genotyped, and PLINK format files were created using Illumina GenomeStudio v2.0.4. Individuals were excluded from the analysis if their call-rate was <95%, if they were outliers of the absolute value of heterozygosity (>3 SD from the mean) or if sex defined based on heterozygosity of the X chromosome did not match sex in phenotype data^51^. Before imputation, variants were filtered by call-rate <95%, HWE *p* < 1e-4 (autosomal variants only), and minor allele frequency <1%. Genotyped variant positions were in build 37 and were lifted over to build 38 using Picard. Phasing was performed using the Beagle v5.4 software^52^. Imputation was performed with Beagle v5.4 software (beagle.22Jul22.46e.jar) and default settings. Dataset was split into batches 5000. A population-specific reference panel consisting of 2695 WGS samples was utilized for imputation, and standard Beagle hg38 recombination maps were used^51^. Based on principal component analysis, samples who were not of European ancestry were removed. Duplicate and monozygous twin detection was performed with KING 2.2.7^53^, and one sample was removed out of the pair of duplicates.

#### Million Veteran Program

Genotyping for MVP has been performed and released in batches as sample recruitment is ongoing. The analyses here were conducted with MVP Release 4 data. Genotyping was conducted using a custom Affymetrix Axiom Biobank Array. The MVP Genomics working group performed quality control and imputation^54^. Variants were removed if they deviated from the expected allele frequency or had a low call rate, and individuals were removed if they had a missing call rate >2.5% or excessive heterozygosity. Phasing and imputation were performed with EAGLE v2^55^ and Minimac4^56^ using the trans-omics for precision medicine (TOPMed) (version r2) reference panel^57^. Genetically inferred ancestry was used to define ancestry groups (AA, EA, and HA)^58^.

#### MGB Biobank

Among the enrolled patients, 64,639 patients had provided blood samples available for genotyping. DNA from samples was genotyped using the Infinium Global Screening Array-24 version 2.0 (Illumina). Imputation was performed using the Michigan Imputation server with the TOPMed (version r2) reference panel, and haplotype phasing was performed using Eagle version 2.3^55^. As previously described, the genetic data were quality controlled, excluding low-quality genetic markers and samples^59^. Pairs of related individuals (kinship > 0.0625) were identified, and one sample from each related pair was excluded. Using TRACE and the Human Genome Diversity Project, principal components of ancestry were computed to correct for the population substructure. Participants of non-European ancestry (24.9% of the cohort) were excluded from the analysis to limit genetic heterogeneity in the present study.

#### FinnGen

The FinnGen sample set consists of prospective samples and legacy samples. Prospective samples were collected during routine diagnostic sampling in hospitals (hospital biobank or Terverystalo Biobank collections), in conjunction with blood donation (Blood Service Biobank), or in conjunction with the collection of research samples (THL Biobank). Legacy samples are older cohorts collected for a specific project, before the Finnish Biobank Act came into effect (September 2013).

Prospective samples were genotyped with the FinnGen ThermoFisher Axiom custom array at the ThermoFisher genotyping service in San Diego, CA, USA, while legacy samples were genotyped using several different Illumina and Affymetrix GWAS arrays. Detailed information about genotyping methods can be found in the FinnGen flagship paper^60^. Genotype data was imputed with a Finnish population-specific Sisu reference panel (SisuV4)^61^. Genetic data were quality controlled, excluding low-quality samples and genetic markers. Detailed information on genotype and sample QC can be found in the FinnGen flagship paper^60^.

### Phenotype derivation

#### UK Biobank

In the UK Biobank cohort, blood glucose levels were taken from data field 30740, which was available in a total of 445,216 participants. To correct the phenotype for sociodemographic and environmental factors, a similar procedure to a previous genetic study of UK Biobank blood traits^27^. To summarize, the raw glucose measure was first log-transformed before the effect of covariates were removed by linear regression. The covariates adjusted for were

- Sample dilution factor (factor variable) calculated by quantiling (*n* = 20) data field 30897
- Self-report fasting time (factor, data field 74) with those reporting 0 and 1 combined into a single category, and those reporting 18 or more combined into another single category
- Genotype batch (factor, data field 22000)
- Ethnicity (factor, data field 21000) with values 4, 2, 1, 3, and −1 reassigned as 4003, 2004, 1003, 3004, and 6, respectively
- Age indicator (factor) set to 50 if age of assessment center attendance (data field 21003) is 50 or below, set to 78 if age attendance is 78 or above and otherwise set to age at attendance
- Age bin (factor, field 21003) set as quintiles of age attended assessment center (data field 21003)
- BMI indicator (factor) calculated by quantiling (*n* = 50) the result of participant’s weight at attendance in kilograms (data field 21002) divided by the squared height (m) at attendance (data field 50)
- waist-hip ratio corrected for BMI (“WHRadjBMI”, factor) calculated by quantiling (*n* = 50) the residuals from a linear regression of log-scaled hip/waist circumference (i.e. data field 49 divided by data field 48) against BMI (calculated as participant’s weight at attendance in kilograms [data field 21002] divided by squared height (m) at attendance [data field 50])
- Center of attendance (factor, data field 54)
- Sex (factor, data field 31)
- Month-year indicator (factor) representing a different category for each month and year of attendance (identified from data field 53), with all months of 2006 grouped as a single factor and August to October 2010 as another single factor
- Sex–age indicator interaction (using age indicator as defined above)
- Sex–fasting time interaction (using fasting time as defined above)
- Sex–ethnicity interaction (using ethnicity as defined above)
- Sex–BMI indicator interaction (using BMI indicator as defined above)
- Sex–WHRadjBMI interaction (using WHRadjBMI as defined above)
- Age bin–fasting time interaction (using fasting time as defined above)
- Glucose assay date (factor, data field 30741)
- Glucose aliquot (factor, data field 30742)

The residuals from this large regression were used as the adjusted (log-scale) glucose measurement. The inverse-normalized phenotype was generated by rank-normalizing these residuals in European-ancestry participants only.

Sleep questions:

**Chronotype**

Do you consider yourself to be?

1. Definitely a “morning” person,
2. More a “morning” than “evening” person,
3. “More an “evening” than “morning” person’,
4. Definitely an “evening” person’.

**Sleep duration**

About how many hours sleep do you get in every 24 h? (include naps).

**Insomnia symptoms**

Do you have trouble falling asleep at night or do you wake up in the middle of the night?

1. Never/rarely,
2. Sometimes,
3. Usually.

**Daytime dozing**

How likely are you to doze off or fall asleep during the daytime when you don’t mean to? (e.g., when working, reading or driving)

1. Never/rarely,
2. Sometimes,
3. Often,
4. All of the time.

**Ease of awakening**

On an average day, how easy do you find getting up in the morning?

1. Not at all easy
2. Not very easy
3. Fairly easy
4. Very easy
5. Prefer not to answer.

**Napping**

1. Do you have a nap during the day?
2. Never/rarely,
3. Sometimes,
4. Usually,
5. Prefer not to answer.

**Estonian Biobank**

In the Estonian Biobank cohort, the glucose measurement values and timepoints were obtained from electronic health records (LOINC laboratory test code 14749-6 “glucose in serum or plasma”). For each participant, we opted to use the earliest possible measurement. Participants with glucose levels below <1 and above >15 mmol/l were excluded from the study.

#### Million Veteran Program

In MVP, the glucose measurement values and timepoints were obtained from a curated LabWAS dataset. For each participant, we opted to use the first recorded random glucose measurement that was available in their record. The inverse-normalized glucose phenotype was generated by rank-normalizing glucose values in participants of European, African American, and Hispanic ancestry.

#### MGB Biobank

In the Mass General Brigham Biobank, glucose measurement values were time-stamped and retrieved from electronic health records. From the EHR data, diabetes status was determined from relevant ICD-9 and -10 codes and individuals with diabetic status were removed from analysis. General linear models for untransformed glucose test results were performed on four glucose test types (plasma glucose (Test:mcsq-pglu), glucose (Test:bcpglu), GLUCOSE blood (Test:el:5200010610), GLUCOSE (Test:bc1-4)) using PLINK v2.00a6LM (4 May 2024)^62^. Models were run using the ‘omit-ref’ modifier to use alt alleles for rs10830963 and rs12419690. Covariates for the analysis were 10 principle components using a previously derived random forest classifier model for European ancestry, as well as age, sex, and the closest stored BMI value in the MGB biobank for each individual. The covariates were also standardized using PLINK2’s --covar-variance-standardize parameter.

#### FinnGen

In FinnGen, glucose measurement values were obtained from a nationwide database of laboratory values (“Kanta”) matched to FinnGen participant IDs. Laboratory-specific codes were harmonized to Observational Medical Outcomes Partnership (OMOP) Common Data Model (CDM) concepts^63^. OMOP data concept 3013826 (glucose [moles/volume] in serum or plasma) was obtained for all FinnGen participants. Extreme values (>500 mmol/L or <1 mmol/L) were excluded, as were measurements taken at times with unusually large numbers of samples at that time point (7:00–7:05 am), likely reflecting a dummy time stamp and not the true sampling time for the individual. Type 2 diabetes, type 1 diabetes, and gestational diabetes status for each participant was obtained from FinnGen core endpoints extracted from electronic health records, and glucose measures from any individual with one or more diabetic diagnoses were excluded from analysis (final *n* = 298,677). After the removal of diabetic individuals, all glucose measures were below 7 mmol/L in all individuals.

### Genome-wide association analyses

To assess whether genetic variants have a time-dependent effect on glucose levels, we performed two types of genome-wide association analyses in the UK Biobank, which utilized the blood sample collection time.

#### Sinusoidal analysis

For primary analyses, we calculated genetic associations by analysing the residualised glucose phenotype using REGENIE v3.1.1^64^ with a sinusoidal GxE interaction term (REGENIE step 2 “--interaction” flag), where the interaction term was defined as either $\sin (2\pi t)$ or $\cos (2\pi t)$ where $t$ is the proportion of the day passed (since midnight) at blood draw time, calculated as

$$t=[({hh}\,{\times}\,3600)+({mm}\,{\times}\,60)+{ss}]/(24\,{\times}\,3600)$$

and with hh:mm:ss being the timestamp for blood sample collection, extracted from data field 3166. Ideally, both sine and cosine interaction terms would be included in the same REGENIE association test model, as they capture effects that are out of phase with one another and so together can capture time-of-day-interaction effects across the entire day. REGENIE only allows a single interaction effect, so the sine and cosine interaction terms were each assessed in separate REGENIE runs. For these analyses, we report the “ADD-INT_SNPxVAR” test results output by REGENIE, which represent the additive effect of the variant on the phenotype as a function of the interaction term, corrected for the variant’s additive effect independent of the interaction term.

In both the sine and cosine interaction analyses, we adjusted for the categorical covariates sex (data field 31), genotyping array (3 categories derived from data field 22000; UKBiLEVE [batches −11 to −1], UKB Axiom initial release [batches 1–22] and UKB Axiom full release [batches 23–95]), center of attendance (data field 54), self-report fasting time (derived from data field 74 as described above) and continuous covariates age at assessment (data field 21003) and genetic principal components 1–10 (data field 22009).

To visualize the changing effect of the *MTNR1B* rs10830963 variant over the day, we also performed a genome-wide association study for 1 h sliding windows of 15 min. In other words, each GWAS consisted of individuals whose blood draw occurred in that one hour window, incremented by 15 min (e.g., all individuals with a blood draw between 9:00–10:00, 9:15–10:15, etc). The sample sizes for these GWASes are as follows: 9:00–10:00 26,431, 9:15–10:15 30,996, 9:30–10:30 33,671, 9:45–10:45 35,820, 10:00–11:00 37,930, 10:15–11:15 40,522, 10:30–11:30 43,429, 10:45–11:45 45,533, 11:00–12:00 47,063, 11:15–12:15 47,127, 11:30–12:30 46,084, 11:45–12:45 45,165, 12:00–13:00 44,198, 12:15–13:15 43,993, 12:30–13:30 44,340, 12:45–13:45 44,587, 13:00–14:00 45,149, 13:15–14:15 45,779, 13:30–14:30 46,605, 13:45–14:45 47,670, 14:00–15:00 48,259, 14:15–15:15 48,037, 14:30–15:30 47,294, 14:45–15:45 46,569, 15:00–16:00 46,323, 15:15–16:15 46,757, 15:30–16:30 47,484, 15:45–16:45 47,616, 16:00–17:00 46,820, 16:15–17:15 45,610, 16:30–17:30 44,139, 16:45–17:45 42,963, 17:00–18:00 4228, 17:15–18:15 41,731, 17:30–18:30 41,465, 17:45–18:45 41,453, 18:00–19:00 40,958. Besides the filter to individuals with a blood draw in that sliding window, all GWAS were performed as above.

These analyses primarily identify time-dependent additive associations, which best capture the effect of variants whose effect allele increases the phenotype when it is higher than the mean level, reduces the phenotype when it is lower than the mean level and has little-to-no effect when the phenotype is near average levels.

#### Goodness-of-fit of the cosinor model

To test the goodness-of-fit of the cosinor model for residualized glucose, we ran a regression replicating the GWAS association run with REGENIE, which does not report goodness-of-fit statistics.

We ran a linear model in R with outcome variable as inverse-normalized adjusted glucose (see “phenotype derivation”, above) and predictors as sine or cosine time, with covariates sex, genotyping array, center of attendance, self-report fasting time, age at assessment, and genetic principal components 1–10. As with the REGENIE analysis, we ran separate models for sine and cosine time. Additionally, we used ANOVA to compare a model containing sine or cosine time with a model containing just covariates.

We found that for the cosinor model including sine time had an *r*^2^ of 0.0098, adjusted *r*^2^ of 0.0097, and F statistic of 140.20. The model including cosine time had an *r*^2^ of 0.010, an adjusted *r*^2^ of 0.0099, and F-statistic of 143.92. When we compared these models to null models including only covariates, however, we found that there was a significant increase in predictive power when including sine time (*p* = 2.026 × 10^−12^) or cosine time (*p* = 2.96 × 10^−34^).

#### Windowed analysis

As a sensitivity analysis, we used an independent method to validate findings from the sinusoidal interaction genetic analyses. To do this, we split individuals into 6 groups based on their time of day of blood draw: [08:00,10:00), [10:00,12:00), [12:00, 14:00), [14:00, 16:00), [16:00, 18:00) and [18:00, 20:00). We then performed a genetic association analysis of the residualised glucose levels for each group independently using REGENIE v3.1.1 and correcting for the same covariates as in the sine and cosine analyses. We then meta-analysed these results.

#### Heritability analysis

We estimated glucose heritability using genome-wide association summary statistics of individuals in per-hour overlapping bins at 15 min intervals using LD Score regression v1.0.1 (LDSC). The precalculated LD Scores for Europeans were used as the LD reference to estimate heritabilities.

#### Analysis of the effect of MTRN1B rs10830963 on HbA1c

Linear regression was performed in R using UK Biobank participant HBA1C levels, from field p30750_i0, and binned into 1 h intervals with 15 min overlapping windows (i.e., 9:00–10:00, 9:15–10:15) using the first recorded blood extraction value, from field p3166_i0 (arrays 0–6) (*n* = 25,995–45,728, depending on bin). Genotype values for rs10830963 and rs12419690 were extracted using PLINK v2.0.0-a.7LM 64-bitIntel. Covariates included were age, sex, genotyping_array, and ten principle components for within-EUR analysis that were derived from a random forest model analysis on ancestry definition in the UKB.

### Replication analysis

#### Estonian Biobank

For the replication analyses, we divided the Estonian Biobank measurements into 24 groups according to the measurement time point hours. Association analyses in Estonian Biobank were carried out for all variants with an INFO score > 0.4 using the additive model as implemented in REGENIE v3.2 with standard quantitative trait settings^64^. Linear regression was carried out with adjustment for current age, age², sex, and 10 PCs as covariates, analyzing only variants with a minimum minor allele count of 2.

#### Million Veteran Program

For the replication analyses, we divided the MVP measurements into 24 groups according to the measurement time point hours. Association analyses in MVP were carried out for all variants that passed quality control using R^64^. Linear regression was carried out separately for each ancestry group (e.g., European, African American, and Hispanic) with adjustment for age, age², sex, and 10 PCs as covariates.

#### MGB Biobank

We divided the MGB Biobank measurements for each hour of the day. Association analysis was carried for all variants passing QC using the additive model as implemented in REGENIE v3.2^64^. We excluded individuals with diagnosis of type 2 diabetes mellitus and adjusted the analysis for age, sex, and population structure.

#### FinnGen

For each hour from 7:00 am to 21:00 (9:00 pm), we separated measures into groups for those sampled during that time point. We then performed a linear regression on glucose measures for the risk variants rs12419690 (*CRY2*) and rs10830963 (*MTNR1B*), adjusting for age at sampling, sex, BMI, genotyping chip, genotyping batch, and the first ten principal components of genetic variation. For the time point analysis, for participants with more than one glucose measure, the sample furthest from 12:00 (noon) was selected for analysis.

For paired analysis, individuals with both a morning sample (7:05–11:00 am) and an evening sample (17:00–20:30, 5:00 pm–8:30 pm) were selected (*n* = 22,456). If an individual had more than one sample in the morning and evening, one sample was randomly chosen from that time point. We then calculated for each sample the difference between morning and evening glucose values (evening–morning) and performed a linear regression on difference in diurnal glucose values for the risk variants rs12419690 (CRY2) and rs10830963 (MTNR1B), adjusting for age death or end of followup, sex, BMI, genotyping chip, genotyping batch, and the first ten principal components of genetic variation. For both time bin analysis and paired analysis, individuals with type 1, type 2, or gestational diabetes were removed.

### Analysis of risk variants in fasting individuals in the UK Biobank

To investigate the role of food consumption mediating the association between *MTNR1B* and *CRY2* risk variants and glucose levels, we subset the individuals in the UKB with glucose measurements to those with reported fasting times of 6, 8, or 12 h at the time of sample collection. Due to substantially smaller sample size of fasting samples, we grouped individuals whose samples were collected before 11 am and those with sample collection after 5 pm. For each time point (before 11 am, after 5 pm) and risk variant (*MTNR1B, CRY2*), we fit a linear regression for adjusted glucose (derived as described in methods, phenotype derivation) with risk genotype and sex, age at baseline, array, center, and the first ten principal components of genetic variance as covariates.

### Analysis of sleep and circadian traits and risk variants

To assess the role of sleep phenotypes in interaction with the risk variants at *MTNR1B* and *CRY2* loci, we assessed questionnaire data from the UK biobank for sleep duration (field ID 1160), insomnia (field ID 1200), chronotype (field ID 1180), napping during the day (field ID 1190), daytime dozing or sleeping (field ID 1220), and ease of awakening in the morning (field ID 1170). For each time point (before 11 am, after 5 pm) and risk variant (*MTNR1B, CRY2*), we fit a linear regression for adjusted glucose (previously described) with risk variant, sleep phenotype, interaction between sleep phenotype and risk variant, and sex, age at baseline, array, center, and first ten principal components of genetic variance as covariates.

### Epidemiological analyses

We examined the associations between melatonin and incident T2DM events using Cox proportional hazard models, with age at onset of melatonin usage (ATC code N05CH01) as a time-dependent covariate and age as the timescale. The cohort included 320,175 participants; 66,464 used melatonin, among whom 4554 (6.9%) developed T2DM, while 15,198 nonusers (6.0%) developed T2DM. We also fitted a Cox model for z-drugs (ATC codes N05CF01, N05CF02, N05CF03, N05CF04), where 82,066 participants were users, of whom 5359 (6.5%) developed T2DM compared with 14,393 nonusers (6.0%). All analyses were adjusted for sex, BMI, and the first ten principal components. Prevalent cases of T2DM were excluded from the Cox regression analyses.

### Reporting summary

Further information on research design is available in the Nature Portfolio Reporting Summary linked to this article.

## Supplementary information

Supplementary Information

Description of Additional Supplementary Files

Supplementary Data 1

Reporting Summary

Transparent Peer Review File

## Data Availability

Accessing individual-level sensitive health data requires authorization from each specific research project for each listed, approved researcher. This study utilizes data from several large biobanks and research projects, we, the authors, are not in a position to grant individual-level data to others.  Generated summary statistics are available in the GWAS Catalog under accession numbers GCST90838682 and GCST90838683.
