# Supplementary material for: Genetic variants affect diurnal glucose levels throughout the day
Source: Nat Commun. 2026 May 22;17:6717. doi: 10.1038/s41467-026-72432-6 (PMC13385737; doi:10.1038/s41467-026-72432-6)
Supplement: Supplementary file 1 — Supplementary Information [file 41467_2026_72432_MOESM1_ESM.pdf]

Supplementary Materials for

Genetic variants affect diurnal glucose levels throughout the day

Nasa Sinnott-Armstrong, Satu Strausz, Lea Urpa, Erik Abner, Josephine P Johnson, Jesse Valliere, Teele Palumaa, FinnGen, Estonian Biobank Research Team, VA Million Veteran Program, Priit Palta, Hassan S Dashti, Kyong-Mi Chang, Marijana Vujkovic, Mark Daly, Jonathan K Pritchard, Richa Saxena, Samuel E Jones & Hanna M Ollila

Corresponding authors: nasa@fredhutch.org; samuel.jones@helsinki.fi; hanna.m.ollila@helsinki.fi

The PDF file includes:

Supplementary Figures 1-23
Supplementary Table 1
List of Consortia

Other Supplementary Materials for this manuscript include the following:

Supplementary Data 1

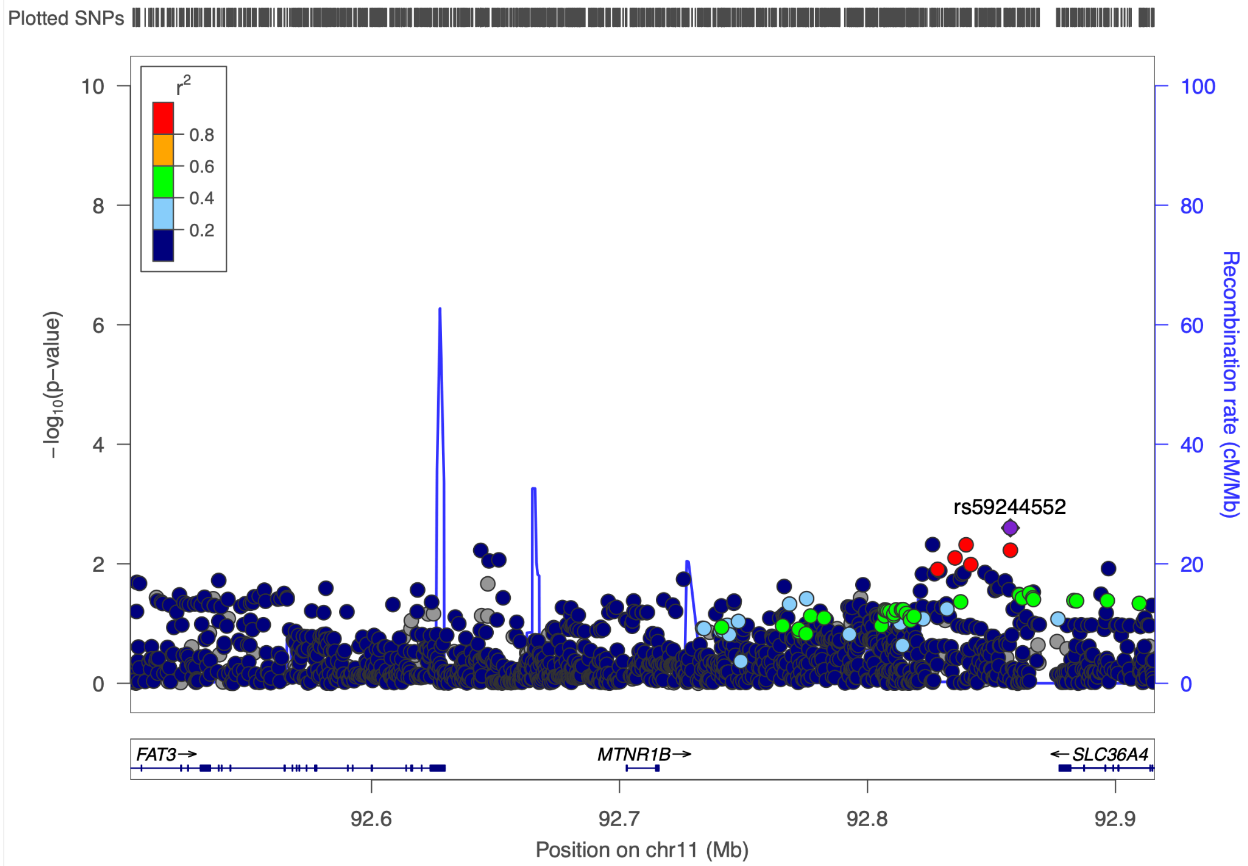

Supplementary Figure 1. Association of variants at the *MTNR1B* locus with glucose levels. We computed genome-wide association statistics for glucose levels (field id = 30740) in the UKBB. The regional analysis did not show significant association with the *MTNR1B* variant (rs10830963 P = 0.27) or other variants at the locus. P-values represent Regenie association summary statistics (main variant effect).

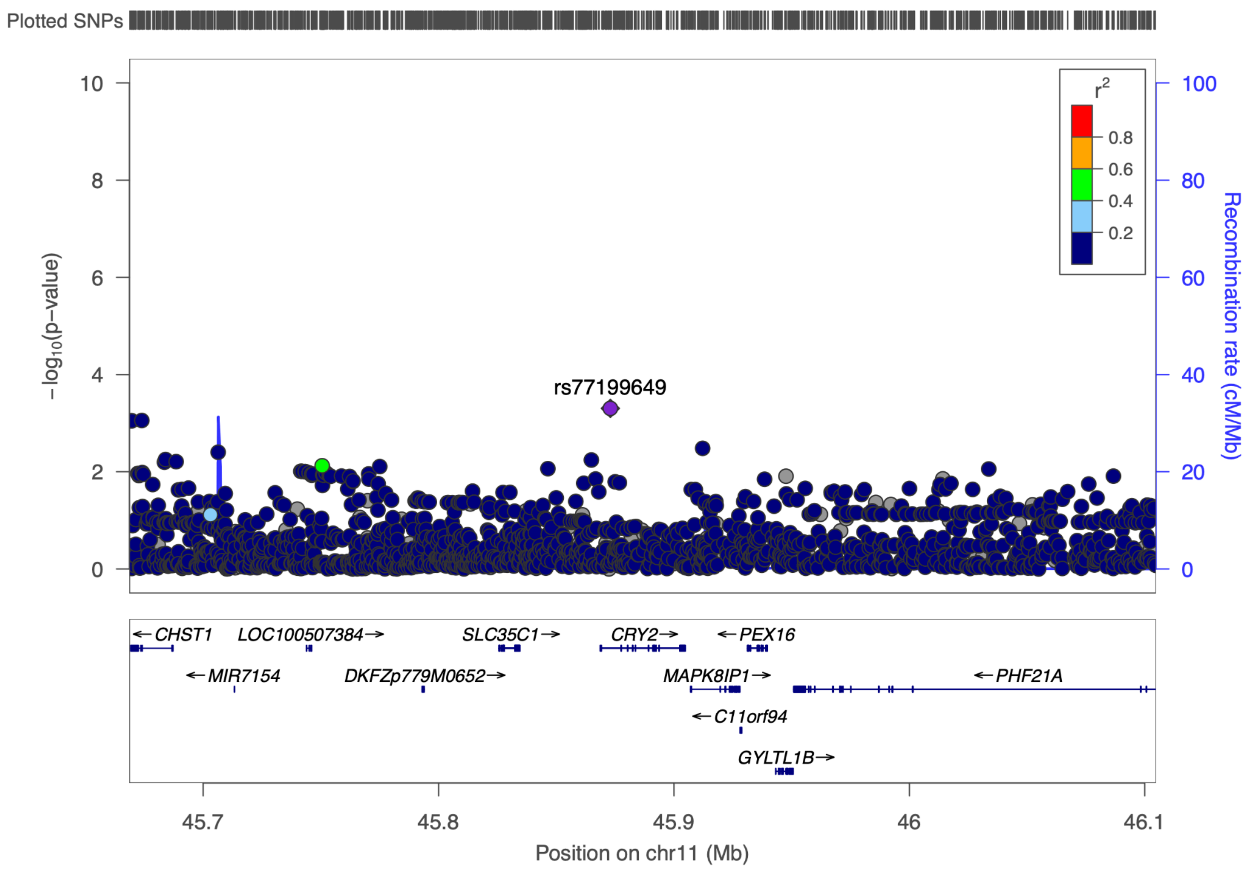

Supplementary Figure 2. Association of variants at the *CRY2* locus with glucose levels. We computed genome-wide association statistics for glucose levels (field id = 30740) in the UKBB. The regional analysis did not show significant association with the *CRY2* variant (rs12419690 P = 0.46) or other variants at the locus. P-values represent Regenie association summary statistics (main variant effect).

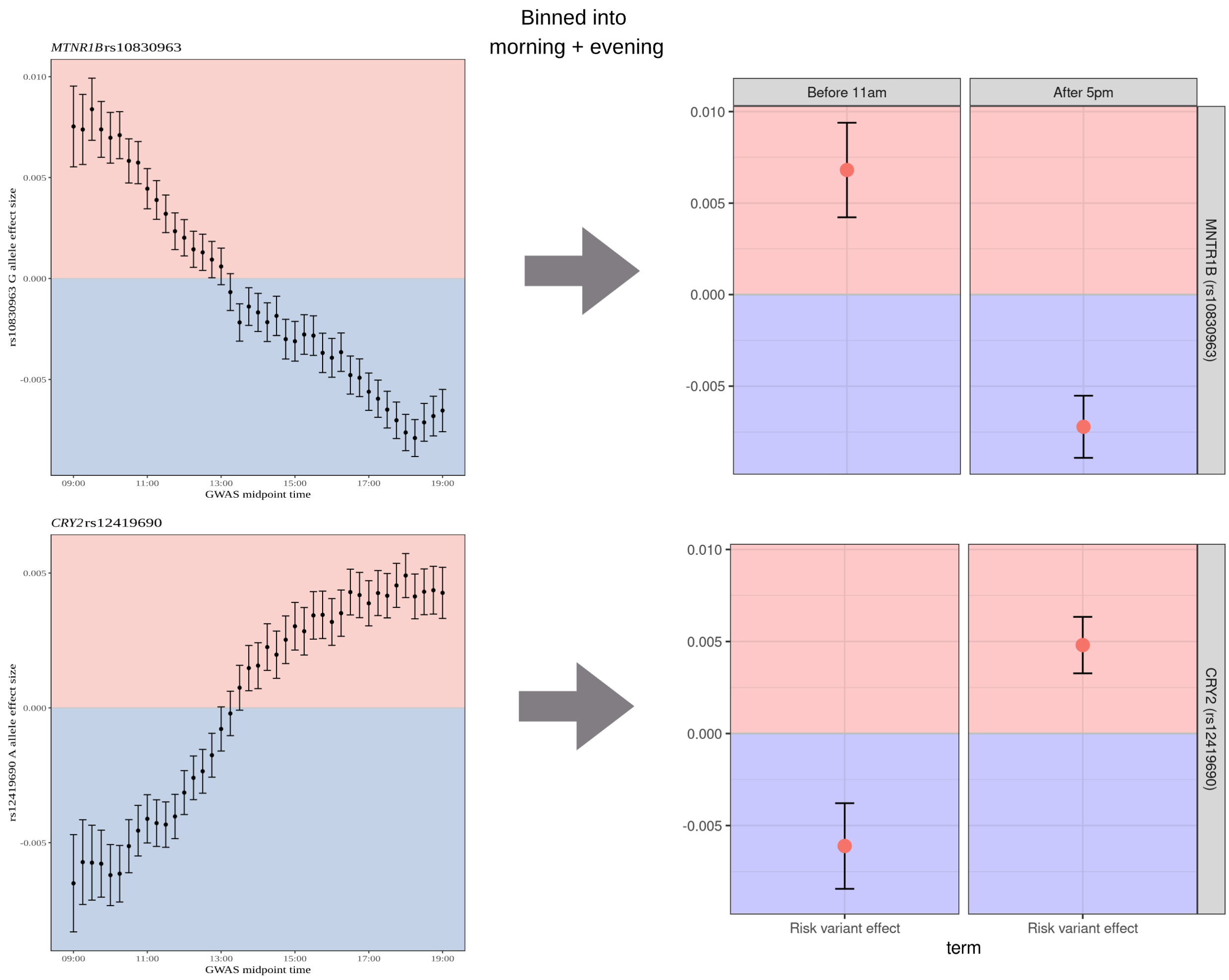

Supplementary Figure 3. Binning process for morning and evening stratification in the UK Biobank. Due to the substantially smaller sample size of individuals fasting in UKBB, we stratified the analysis by sample collected before 11am vs sample collected after 5pm. On left panel, data is presented as GWAS effect size and 95% confidence interval. Sample sizes for each time point are reported in the Methods section.

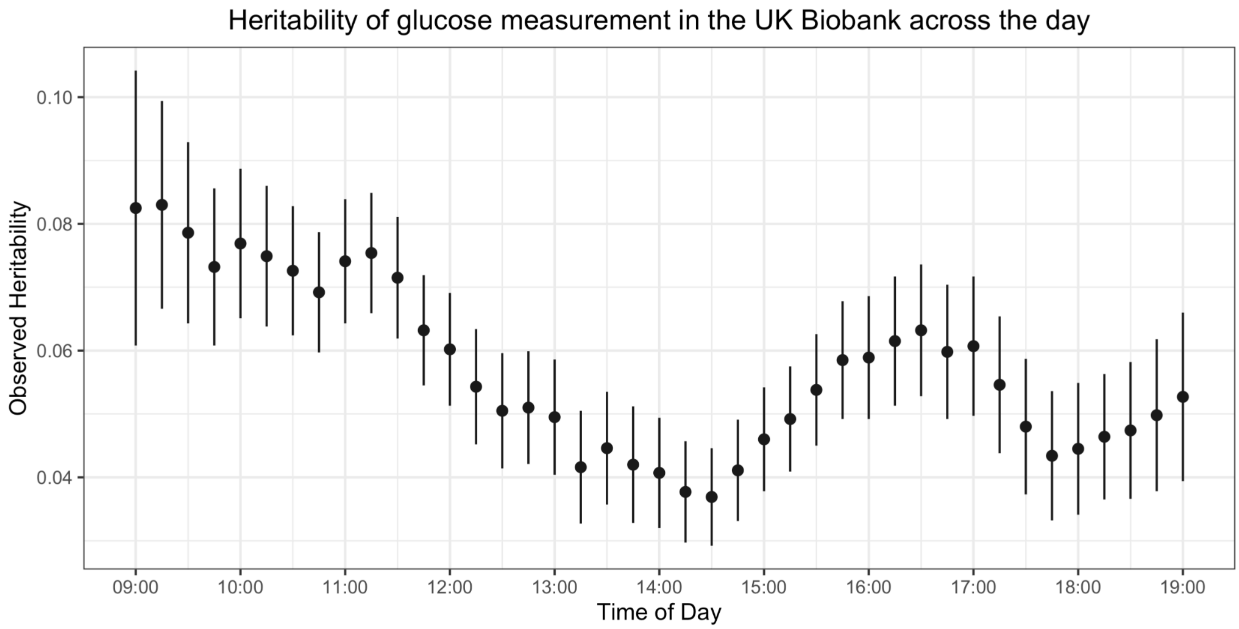

Supplementary Figure 4. Heritability of glucose in UK Biobank throughout the day. Observed heritability from LD score regression, calculated from GWAS of overlapping 1 hour bins throughout the day. Points represent estimated genetic correlation with 95% confidence intervals. Sample size at each bin is presented in Methods.

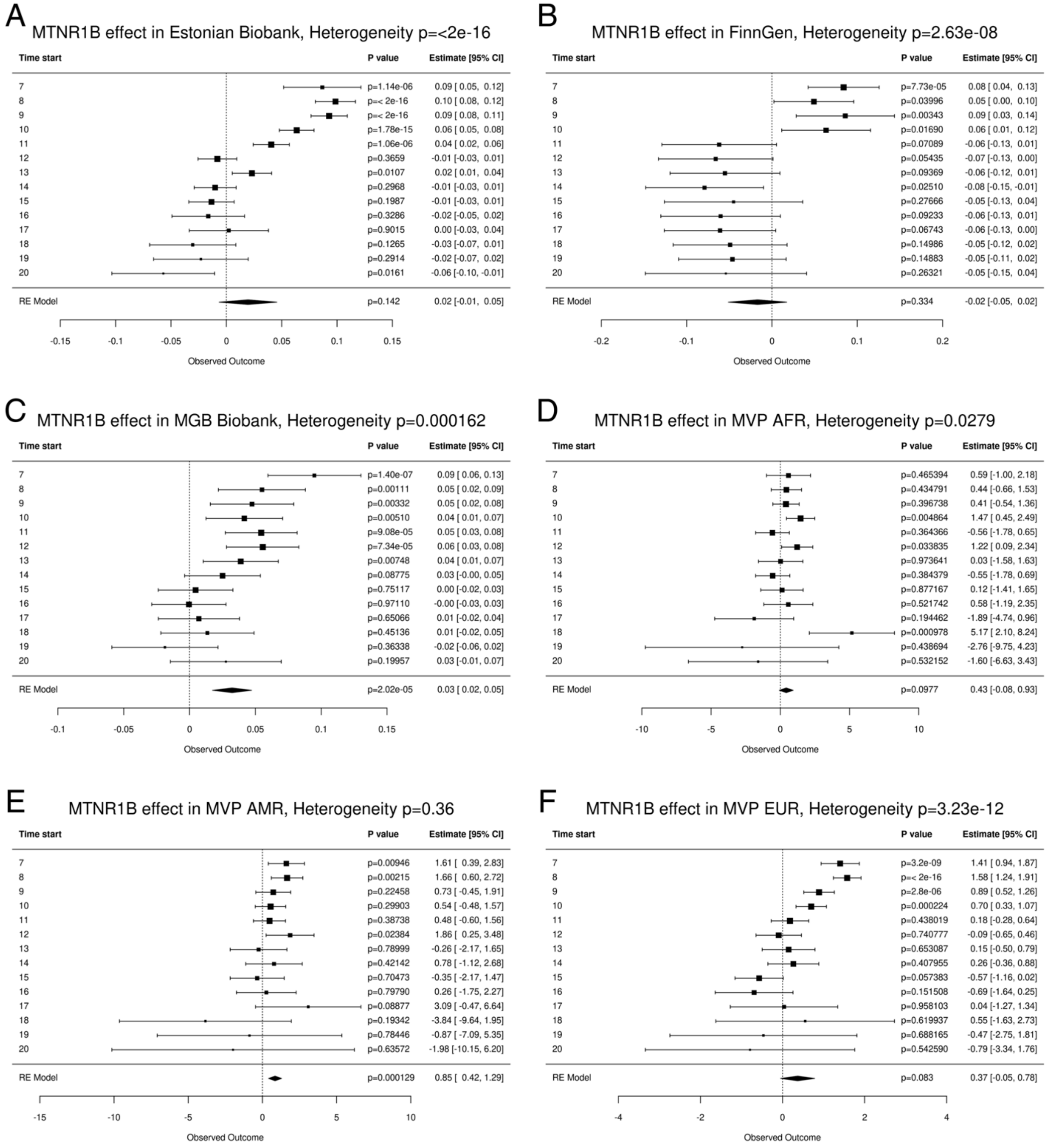

Supplementary Figure 5. Effect of *MTNR1B* risk allele across the day in replication cohorts. Effect of *MTNR1B* risk allele on glucose levels throughout the day in A) Estonian Biobank (n=219,048), B) FinnGen (n=109,024), C) MGB Biobank (n=127,490), and D-F) the VA Million Veteran Program. AFR = African ancestry individuals (n=31,229), AMR = Hispanic ancestry individuals (n=14,680), EUR = European ancestry individuals (n=115,096). In each panel, data is presented as effect size and 95% confidence interval.

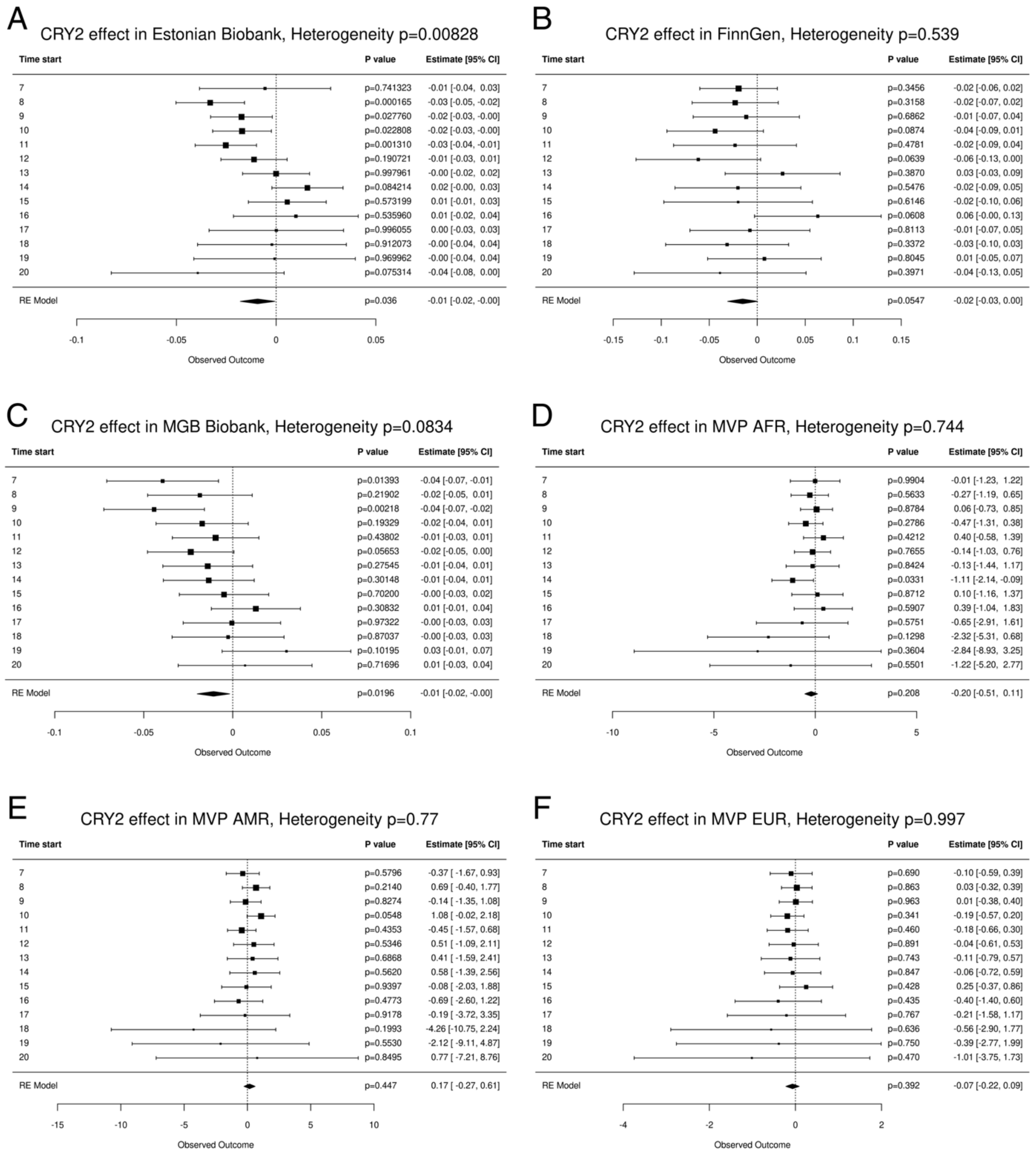

Supplementary Figure 6. Effect of *CRY2* risk allele across the day in replication cohorts. Effect of *CRY2* risk allele on glucose levels throughout the day in A) Estonian Biobank (n=219,048), B) FinnGen (n=109,024), C) MGB Biobank (n=127,490), and D-F) the VA Million Veteran Program. AFR = African ancestry individuals (n=31,229), AMR = Hispanic ancestry individuals (n=14,680), EUR = European ancestry individuals (n=115,096). In each panel, data is presented as effect size and 95% confidence interval.

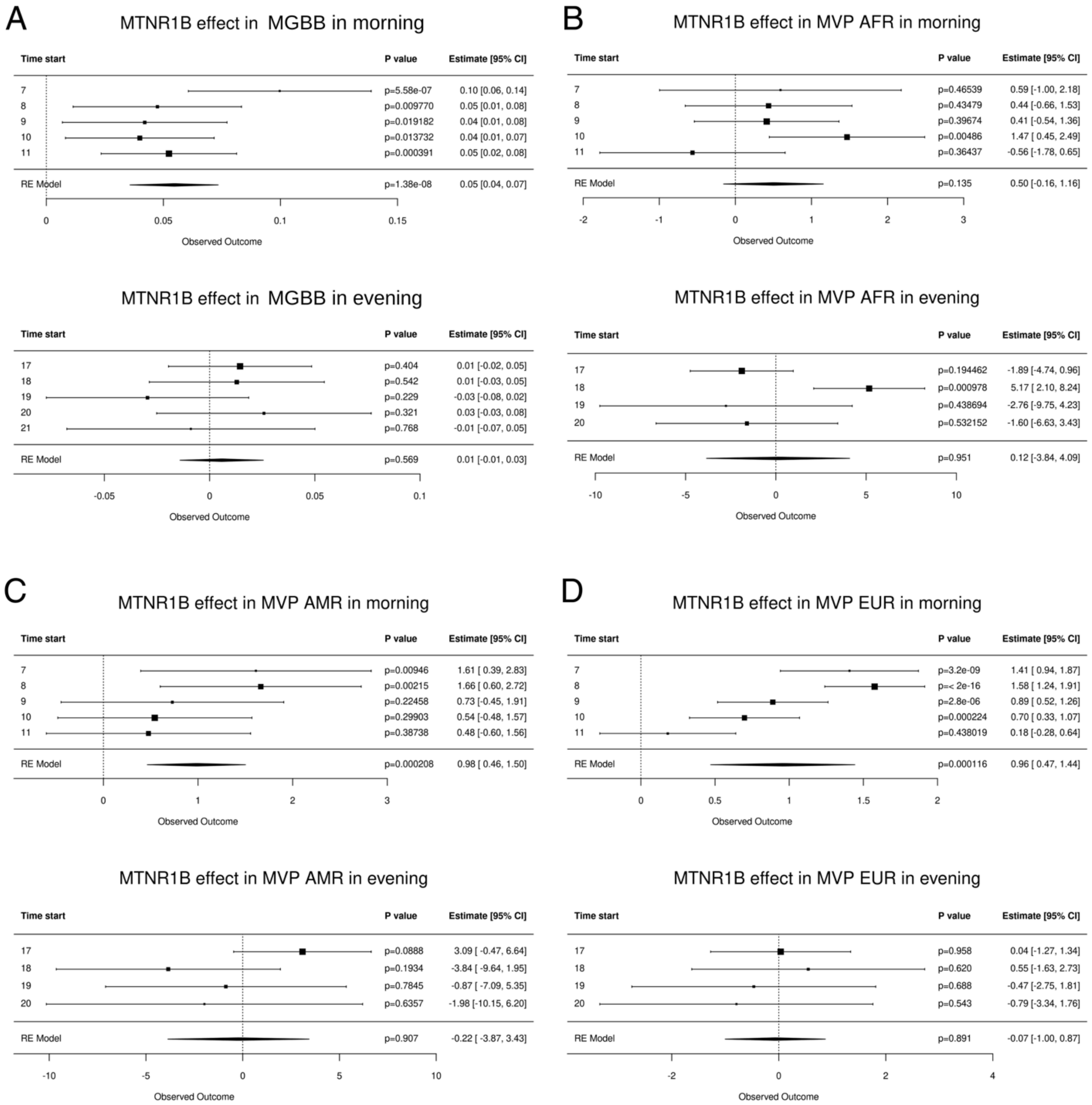

Supplementary Figure 7. Effect of *MTNR1B* risk allele in morning and evening in replication cohorts. Effect of *MTNR1B* risk allele on glucose levels in the morning and evening in A) MGB Biobank (n morning = 43,398, n evening = 36,408) and B-D) the VA Million Veteran Program. AFR = African ancestry individuals (n morning = 18,029, n evening = 1,455), AMR = Hispanic ancestry individuals (n morning = 9,436, n evening = 555), EUR = European ancestry individuals (n morning = 76,120, n evening 3,456). In each panel, data is presented as effect size and 95% confidence interval.

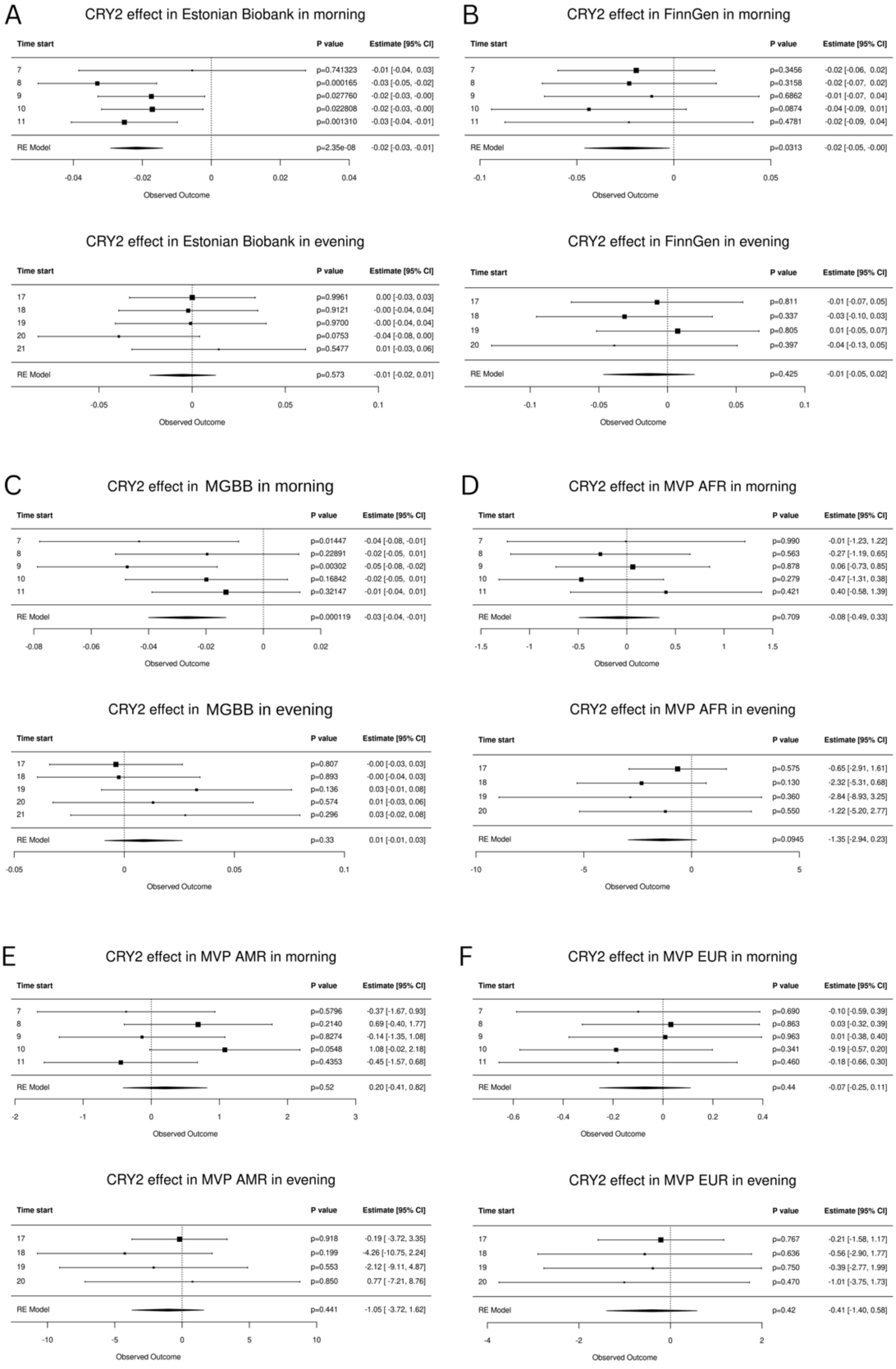
A
CRY2 effect in Estonian Biobank in morning
Time start
P value
Estimate [95% CI]
7 p=0.741323 -0.01 [-0.04, 0.03]
8 p=0.000165 -0.03 [-0.05, -0.02]
9 p=0.027760 -0.02 [-0.03, -0.00]
10 p=0.022808 -0.02 [-0.03, -0.00]
11 p=0.001310 -0.03 [-0.04, -0.01]
RE Model p=2.35e-08 -0.02 [-0.03, -0.01]
-0.04 -0.02 0 0.02 0.04
Observed Outcome
CRY2 effect in Estonian Biobank in evening
Time start
P value
Estimate [95% CI]
17 p=0.9961 0.00 [-0.03, 0.03]
18 p=0.9121 -0.00 [-0.04, 0.04]
19 p=0.9700 -0.00 [-0.04, 0.04]
20 p=0.0753 -0.04 [-0.08, 0.00]
21 p=0.5477 0.01 [-0.03, 0.06]
RE Model p=0.573 -0.01 [-0.02, 0.01]
-0.05 0 0.05 0.1
Observed Outcome
B
CRY2 effect in FinnGen in morning
Time start
P value
Estimate [95% CI]
7 p=0.3456 -0.02 [-0.06, 0.02]
8 p=0.3158 -0.02 [-0.07, 0.02]
9 p=0.6862 -0.01 [-0.07, 0.04]
10 p=0.0874 -0.04 [-0.09, 0.01]
11 p=0.4781 -0.02 [-0.09, 0.04]
RE Model p=0.0313 -0.02 [-0.05, -0.00]
-0.1 -0.05 0 0.05
Observed Outcome
CRY2 effect in FinnGen in evening
Time start
P value
Estimate [95% CI]
17 p=0.811 -0.01 [-0.07, 0.05]
18 p=0.337 -0.03 [-0.10, 0.03]
19 p=0.805 0.01 [-0.05, 0.07]
20 p=0.397 -0.04 [-0.13, 0.05]
RE Model p=0.425 -0.01 [-0.05, 0.02]
-0.1 -0.05 0 0.05 0.1
Observed Outcome
C
CRY2 effect in MGBB in morning
Time start
P value
Estimate [95% CI]
7 p=0.01447 -0.04 [-0.08, -0.01]
8 p=0.22891 -0.02 [-0.05, 0.01]
9 p=0.00302 -0.05 [-0.08, -0.02]
10 p=0.16842 -0.02 [-0.05, 0.01]
11 p=0.32147 -0.01 [-0.04, 0.01]
RE Model p=0.000119 -0.03 [-0.04, -0.01]
-0.08 -0.06 -0.04 -0.02 0 0.02
Observed Outcome
CRY2 effect in MGBB in evening
Time start
P value
Estimate [95% CI]
17 p=0.807 -0.00 [-0.03, 0.03]
18 p=0.893 -0.00 [-0.04, 0.03]
19 p=0.136 0.03 [-0.01, 0.08]
20 p=0.574 0.01 [-0.03, 0.06]
21 p=0.296 0.03 [-0.02, 0.08]
RE Model p=0.33 0.01 [-0.01, 0.03]
-0.05 0 0.05 0.1
Observed Outcome
D
CRY2 effect in MVP AFR in morning
Time start
P value
Estimate [95% CI]
7 p=0.990 -0.01 [-1.23, 1.22]
8 p=0.563 -0.27 [-1.19, 0.65]
9 p=0.878 0.06 [-0.73, 0.85]
10 p=0.279 -0.47 [-1.31, 0.38]
11 p=0.421 0.40 [-0.58, 1.39]
RE Model p=0.709 -0.08 [-0.49, 0.33]
-1.5 -1 -0.5 0 0.5 1 1.5
Observed Outcome
CRY2 effect in MVP AFR in evening
Time start
P value
Estimate [95% CI]
17 p=0.575 -0.65 [-2.91, 1.61]
18 p=0.130 -2.32 [-5.31, 0.68]
19 p=0.360 -2.84 [-8.93, 3.25]
20 p=0.550 -1.22 [-5.20, 2.77]
RE Model p=0.0945 -1.35 [-2.94, 0.23]
-10 -5 0 5
Observed Outcome
E
CRY2 effect in MVP AMR in morning
Time start
P value
Estimate [95% CI]
7 p=0.5796 -0.37 [-1.67, 0.93]
8 p=0.2140 0.69 [-0.40, 1.77]
9 p=0.8274 -0.14 [-1.35, 1.08]
10 p=0.0548 1.08 [-0.02, 2.18]
11 p=0.4353 -0.45 [-1.57, 0.68]
RE Model p=0.52 0.20 [-0.41, 0.82]
-2 -1 0 1 2 3
Observed Outcome
CRY2 effect in MVP AMR in evening
Time start
P value
Estimate [95% CI]
17 p=0.918 -0.19 [-3.72, 3.35]
18 p=0.199 -4.26 [-10.75, 2.24]
19 p=0.553 -2.12 [-9.11, 4.87]
20 p=0.850 0.77 [-7.21, 8.76]
RE Model p=0.441 -1.05 [-3.72, 1.62]
-10 -5 0 5 10
Observed Outcome
F
CRY2 effect in MVP EUR in morning
Time start
P value
Estimate [95% CI]
7 p=0.690 -0.10 [-0.59, 0.39]
8 p=0.863 0.03 [-0.32, 0.39]
9 p=0.963 0.01 [-0.38, 0.40]
10 p=0.341 -0.19 [-0.57, 0.20]
11 p=0.460 -0.18 [-0.66, 0.30]
RE Model p=0.44 -0.07 [-0.25, 0.11]
-0.6 -0.4 -0.2 0 0.2 0.4
Observed Outcome
CRY2 effect in MVP EUR in evening
Time start
P value
Estimate [95% CI]
17 p=0.767 -0.21 [-1.58, 1.17]
18 p=0.636 -0.56 [-2.90, 1.77]
19 p=0.750 -0.39 [-2.77, 1.99]
20 p=0.470 -1.01 [-3.75, 1.73]
RE Model p=0.42 -0.41 [-1.40, 0.58]
-4 -2 0 2
Observed Outcome

Supplementary Figure 8. Effect of *CRY2* risk allele in morning and evening in replication cohorts. Effect of *CRY2* risk allele on glucose levels in the morning and evening A) Estonian Biobank (n morning = 110,739, n evening = 20,149), B) FinnGen (n morning = 49,555, n evening = 30,063), C) MGB Biobank (n morning = 43,398, n evening = 36,408), and D-F) the VA Million Veteran Program. AFR = African ancestry individuals (n morning = 18,029, n evening = 1,455), AMR = Hispanic ancestry individuals (n morning = 9,436, n evening = 555), EUR = European ancestry individuals (n morning = 76,120, n evening 3,456). In each panel, data is presented as effect size and 95% confidence interval.

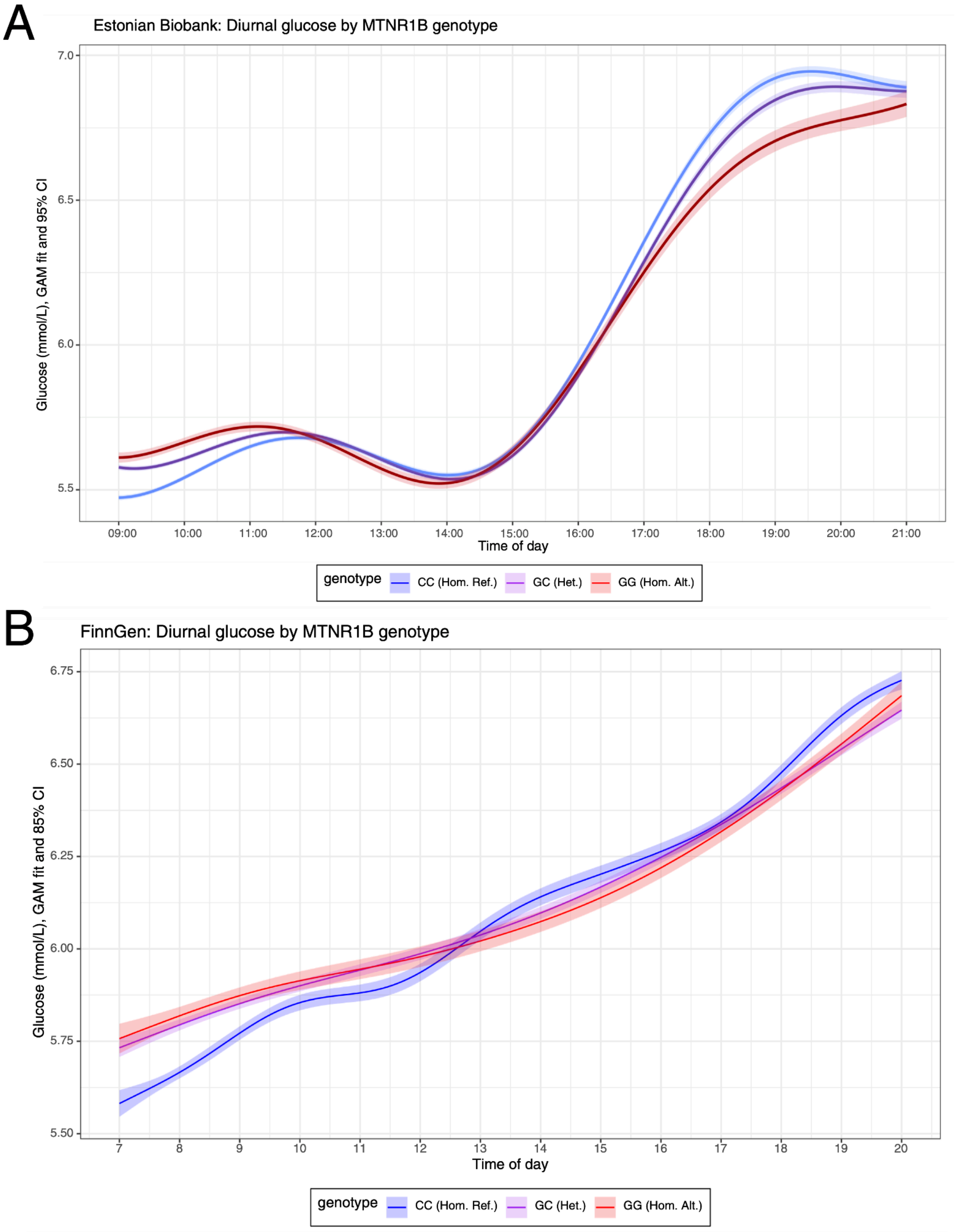

Supplementary Figure 9. Effect of *CRY2* risk allele in morning and evening in replication cohorts. Analysis using generalized linear model (GAM) spline over the day with unadjusted glucose values shows consistent and a higher effect of risk allele rs10830963G in the morning than in the evening in (A) the Estonian Biobank (n=219,048) and (B) FinnGen (n=298,677). Data is presented as values across the day predicted by GAM model with standard error ribbon.

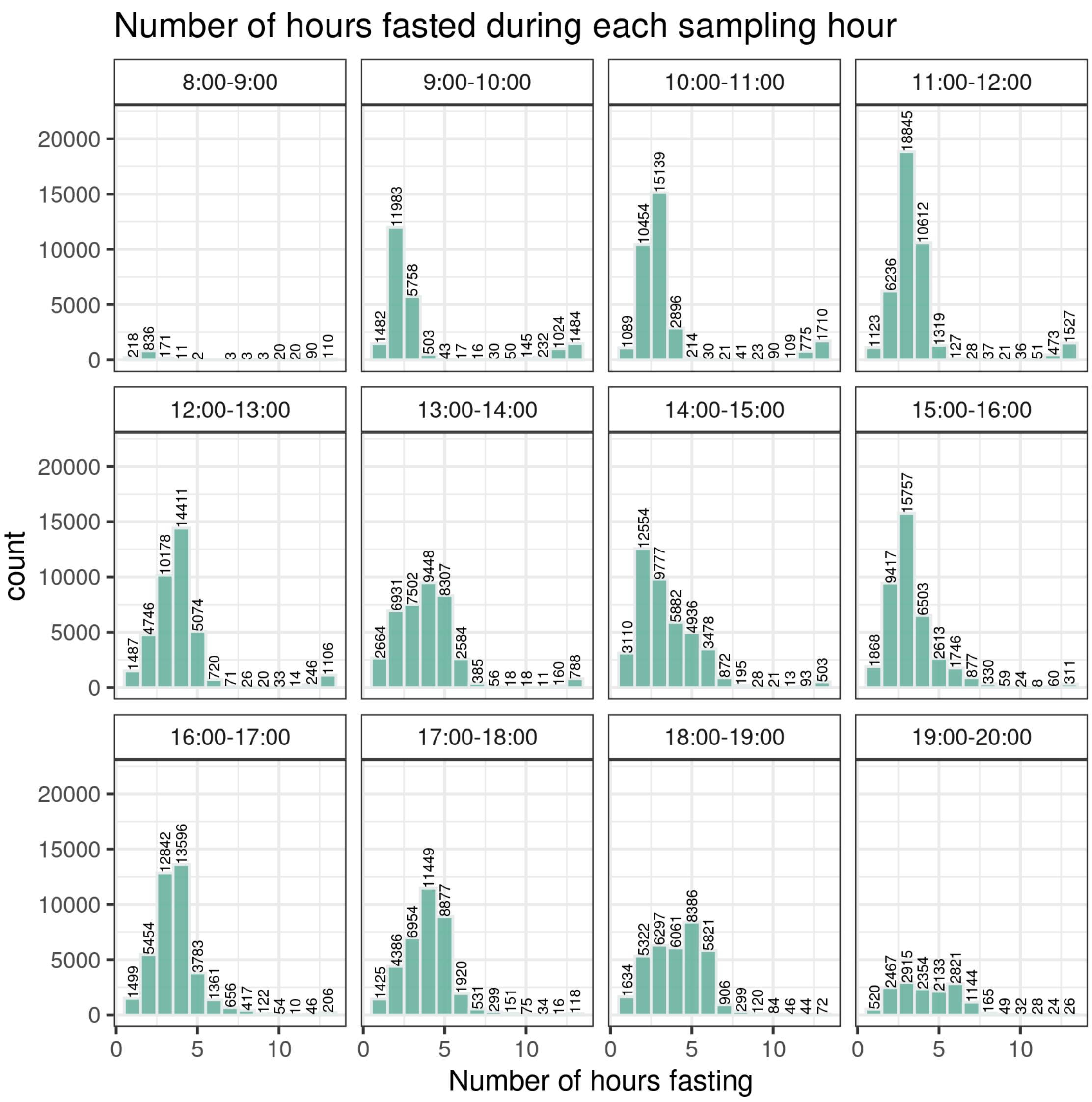

Supplementary Figure 10. **Fasting time in the UK Biobank stratified by time of sample collection.** We binned glucose draw data from the UK Biobank by time of measurement from 8AM to 8PM, and by number of hours fasting. We then computed the number of individuals in each fasting bin by hours of fasting.

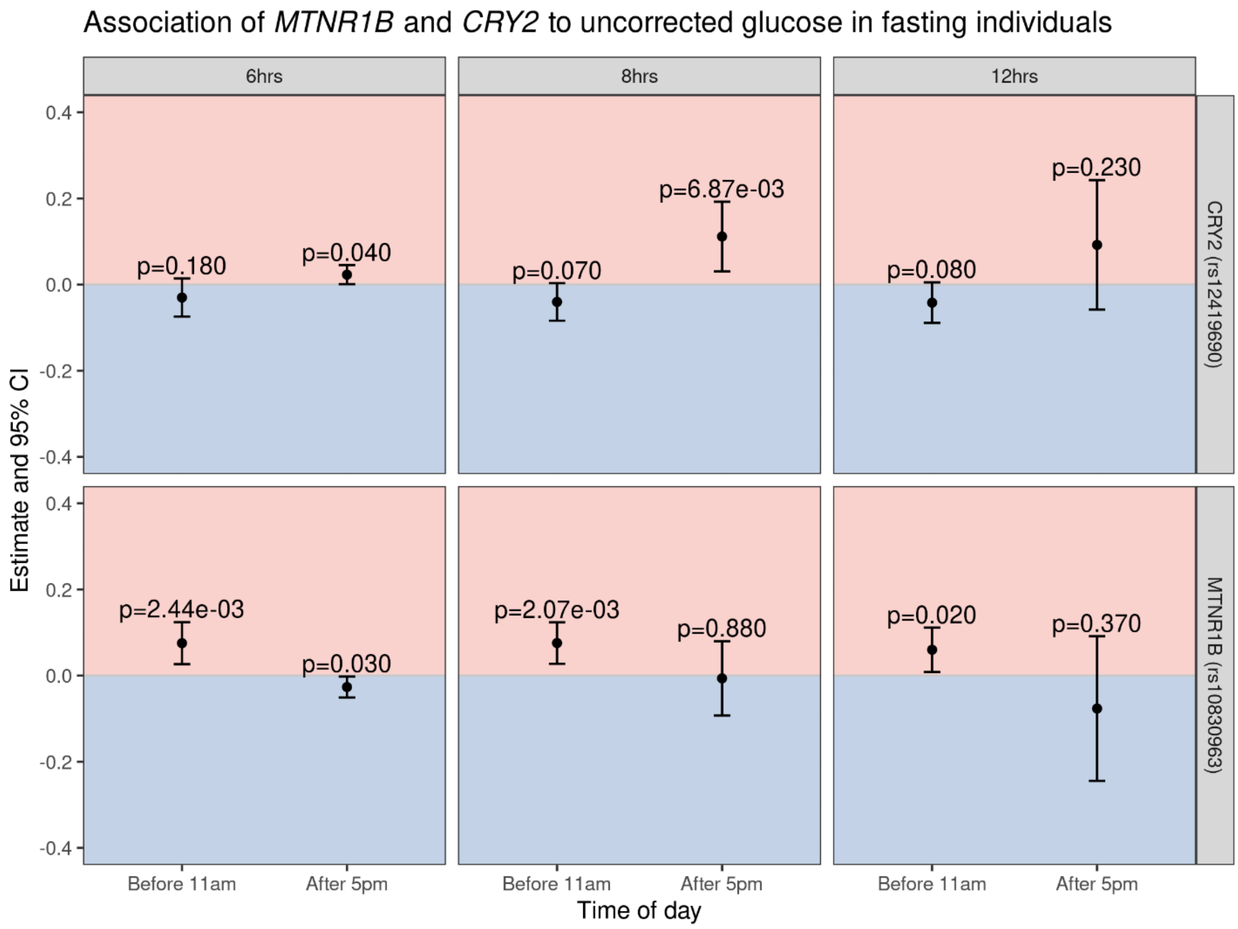

Supplementary Figure 11. Effect of risk alleles in fasting individuals on uncorrected glucose levels in the morning and evening. Effect of risk alleles in fasting individuals with sample collection time before 11AM or after 5PM on unadjusted glucose levels in the UK Biobank. Data is presented as additive genotype effect size and 95% confidence intervals of a linear regression of adjusted glucose with risk genotype at each time point, adjusted by sex and other relevant covariates (see Methods). Red shading highlights morning positive effect sizes, while blue shading highlights negative evening effect sizes.

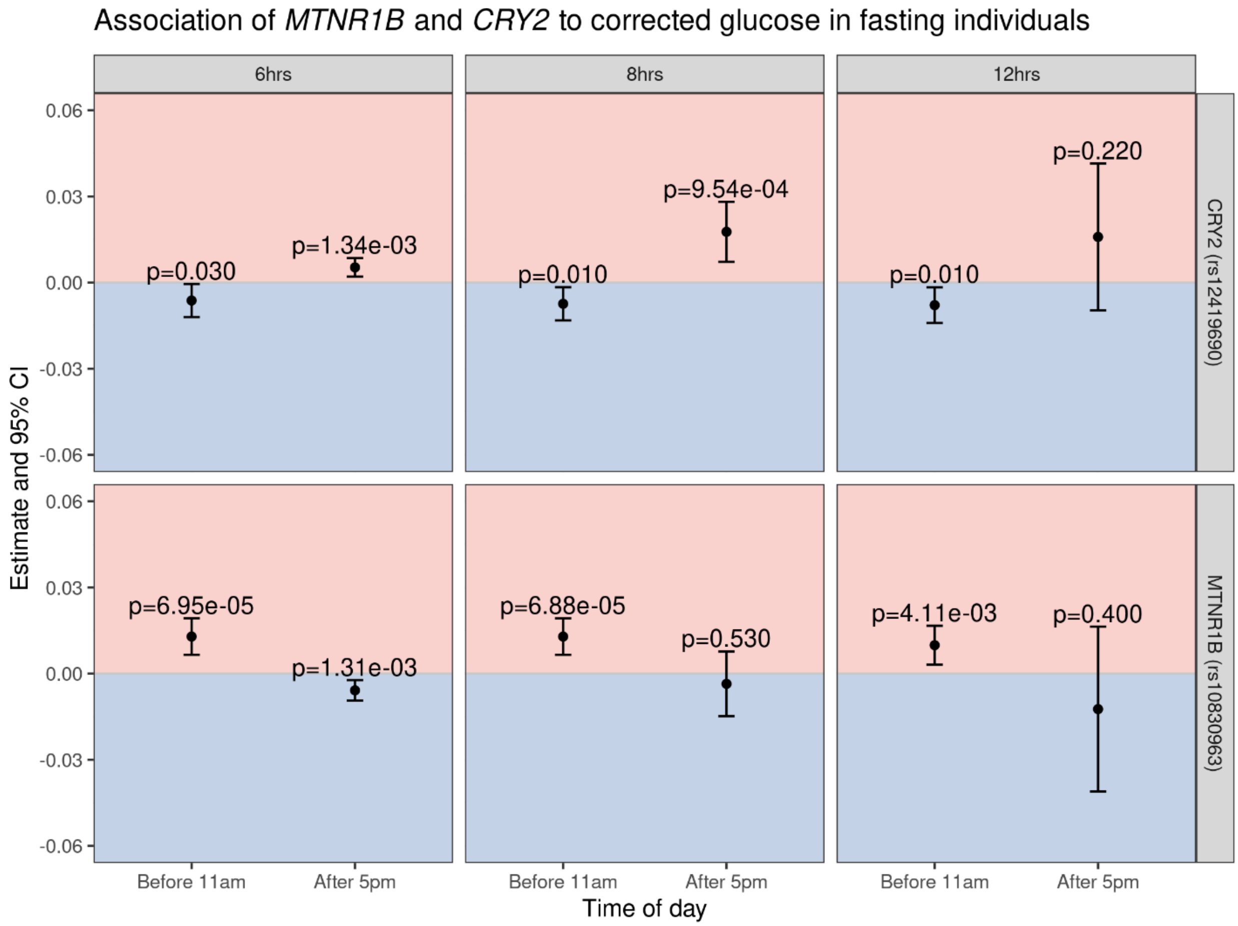

Supplementary Figure 12. Effect of risk alleles in fasting individuals on residual glucose levels in the morning and evening. Effect of risk alleles in fasting individuals with sample collection time before 11AM or after 5PM on residual glucose levels in the UK Biobank (see methods). Data is presented as additive genotype effect size and 95% confidence intervals of a linear regression of adjusted glucose with risk genotype at each time point, adjusted by sex and other relevant covariates (see Methods). Red shading highlights morning positive effect sizes, while blue shading highlights negative evening effect sizes.

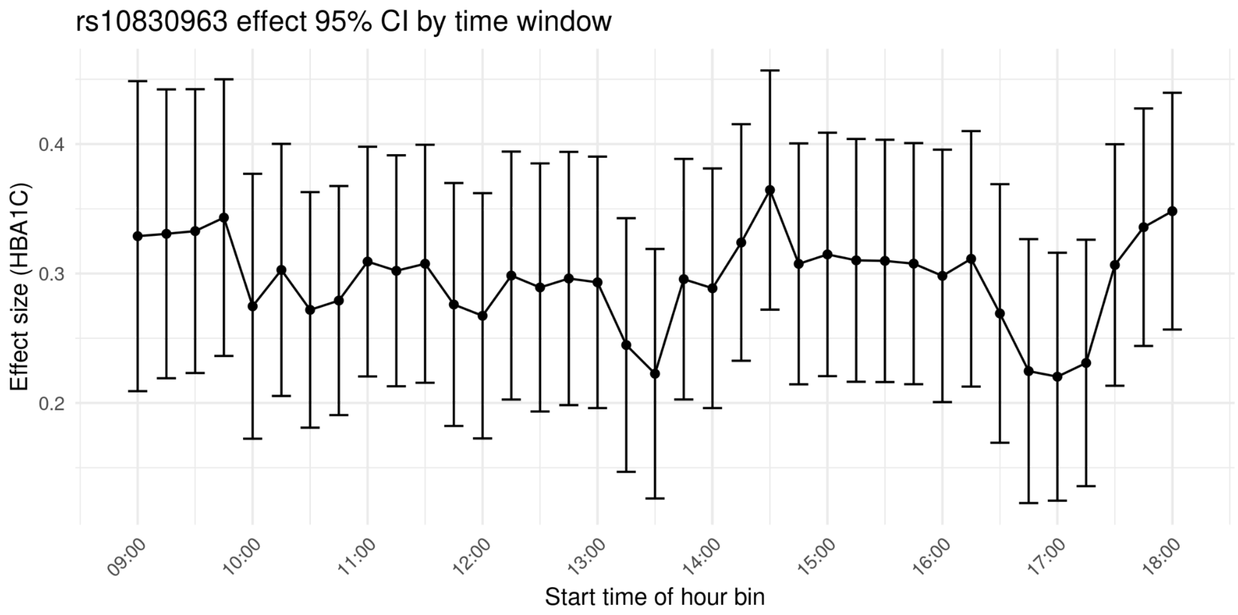

Supplementary Figure 13. Effect of *MTNR1B* rs10830963 on HbA1c throughout the day. Effect of MTNR1B variant on HBA1c in the UK Biobank. Data is presented as additive genotype effect size and 95% confidence intervals.

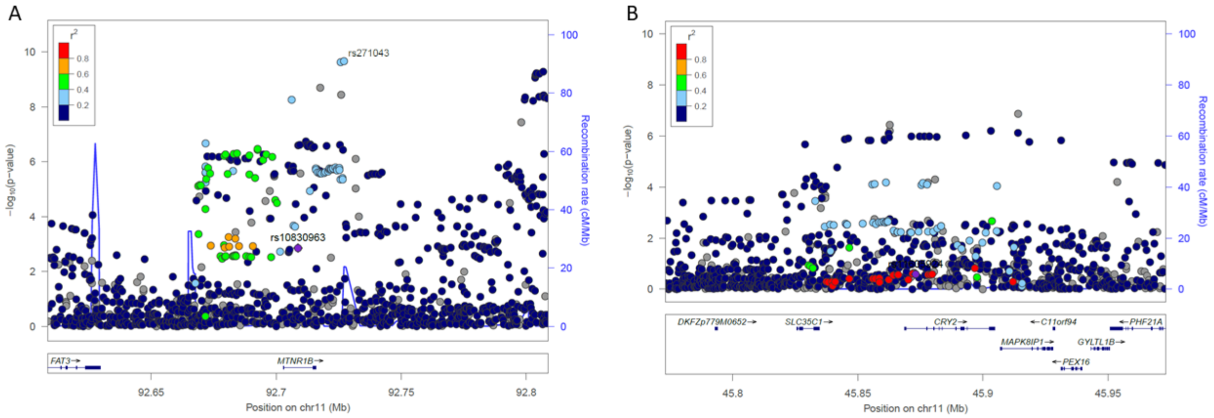

Supplementary Figure 14. Association of *MTNR1B* and *CRY2* loci with chronotype. Regional association plot for chronotype at the A) *MTNR1B* and B) *CRY2*. We observed association both at the *MTNR1B* and at the *CRY2* locus with chronotype. However, the variants that associate with chronotype are not in strong LD (color scale) with the variants that associate with glucose levels.

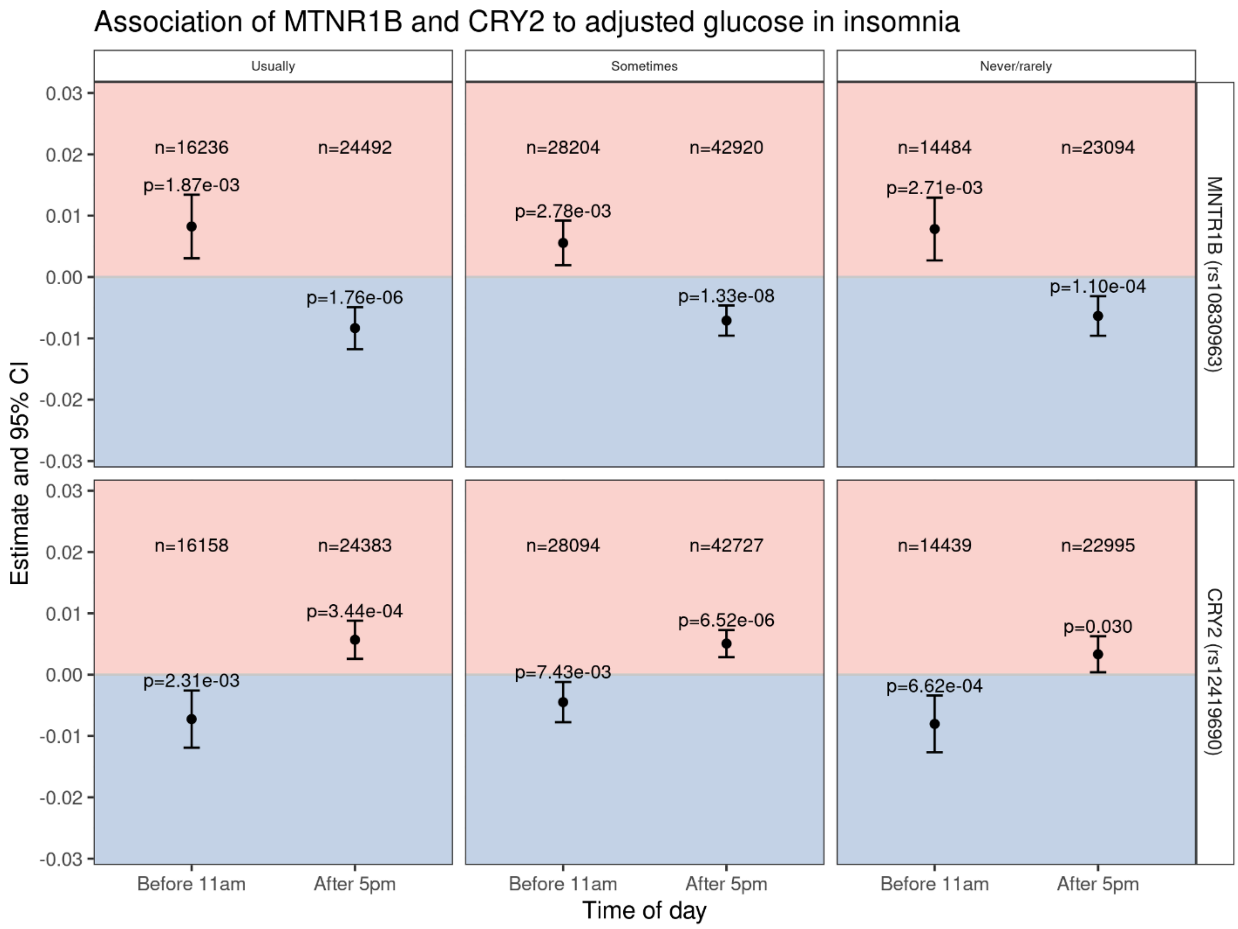

Supplementary Figure 15. Comparison of the association of residual glucose levels in the morning and evening stratified by insomnia. We computed the effect size for *CRY2* and *MTNR1B* risk alleles, stratified by time of day of sampling and insomnia status (as given in UKBB questionnaire data). Data is presented as additive genotype effect size and 95% confidence intervals.

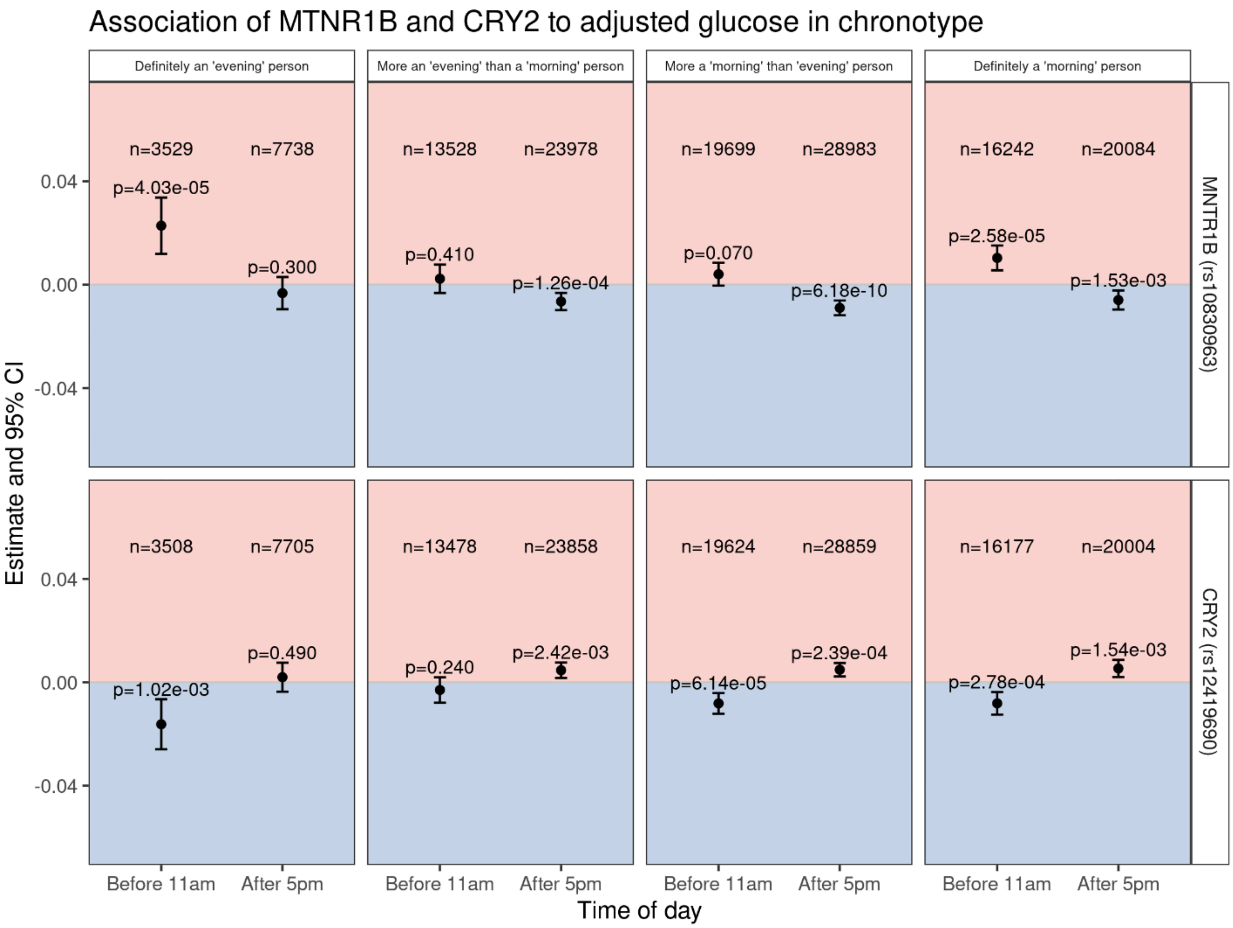

Supplementary Figure 16. Comparison of the association of residual glucose levels in the morning and evening stratified by chronotype. We computed the effect size for *CRY2* and *MTNR1B* risk alleles, stratified by time of day of sampling and chronotype (as given in UKBB questionnaire data). Data is presented as additive genotype effect size and 95% confidence intervals.

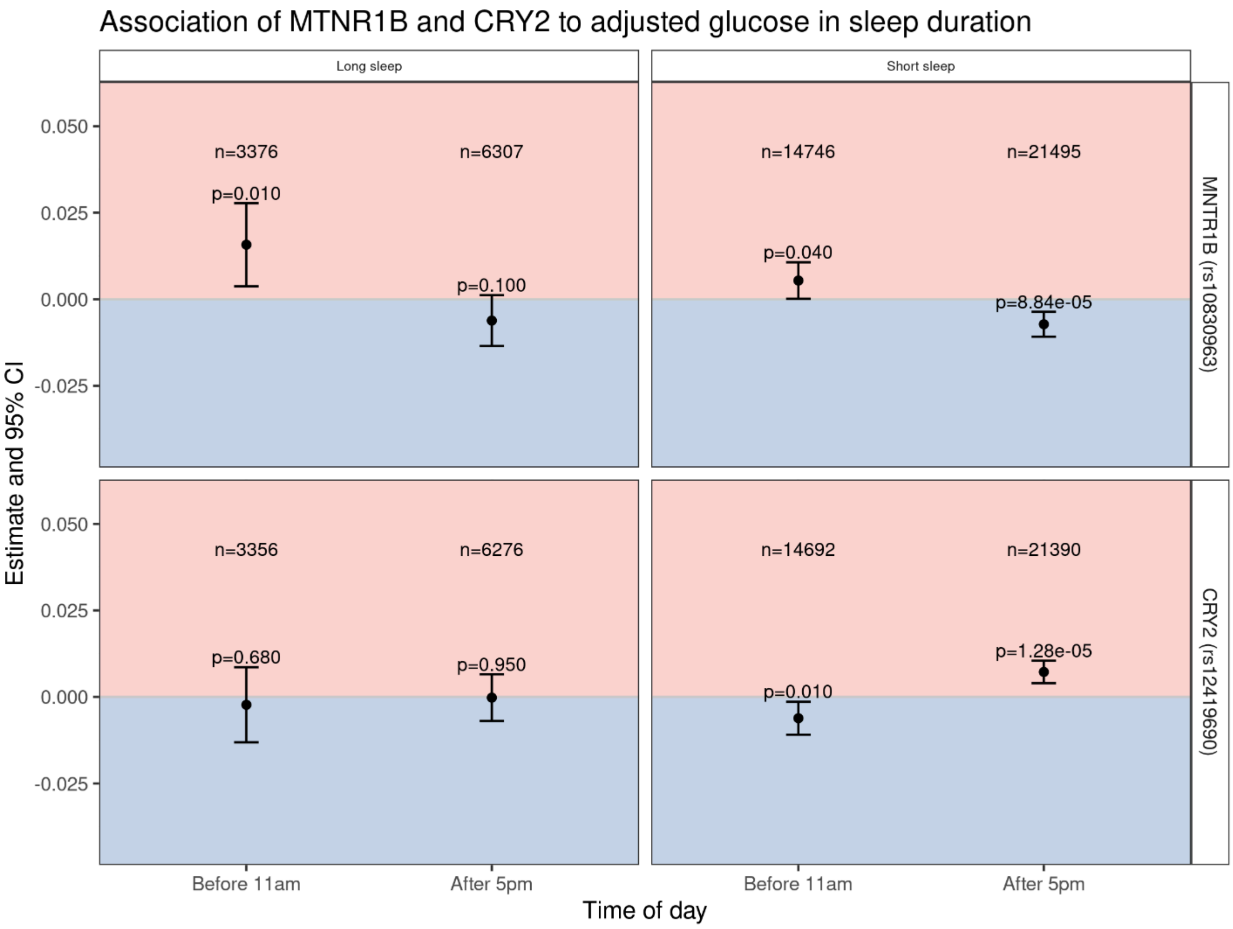

Supplementary Figure 17. Comparison of the association of residual glucose levels in the morning and evening stratified by sleep duration. We computed the effect size for *CRY2* and *MTNR1B* risk alleles, stratified by time of day of sampling and sleep duration (as given in UKBB questionnaire data). Short sleep was defined as 6 hours or less, and long sleep 9 hours or more. Data is presented as additive genotype effect size and 95% confidence intervals.

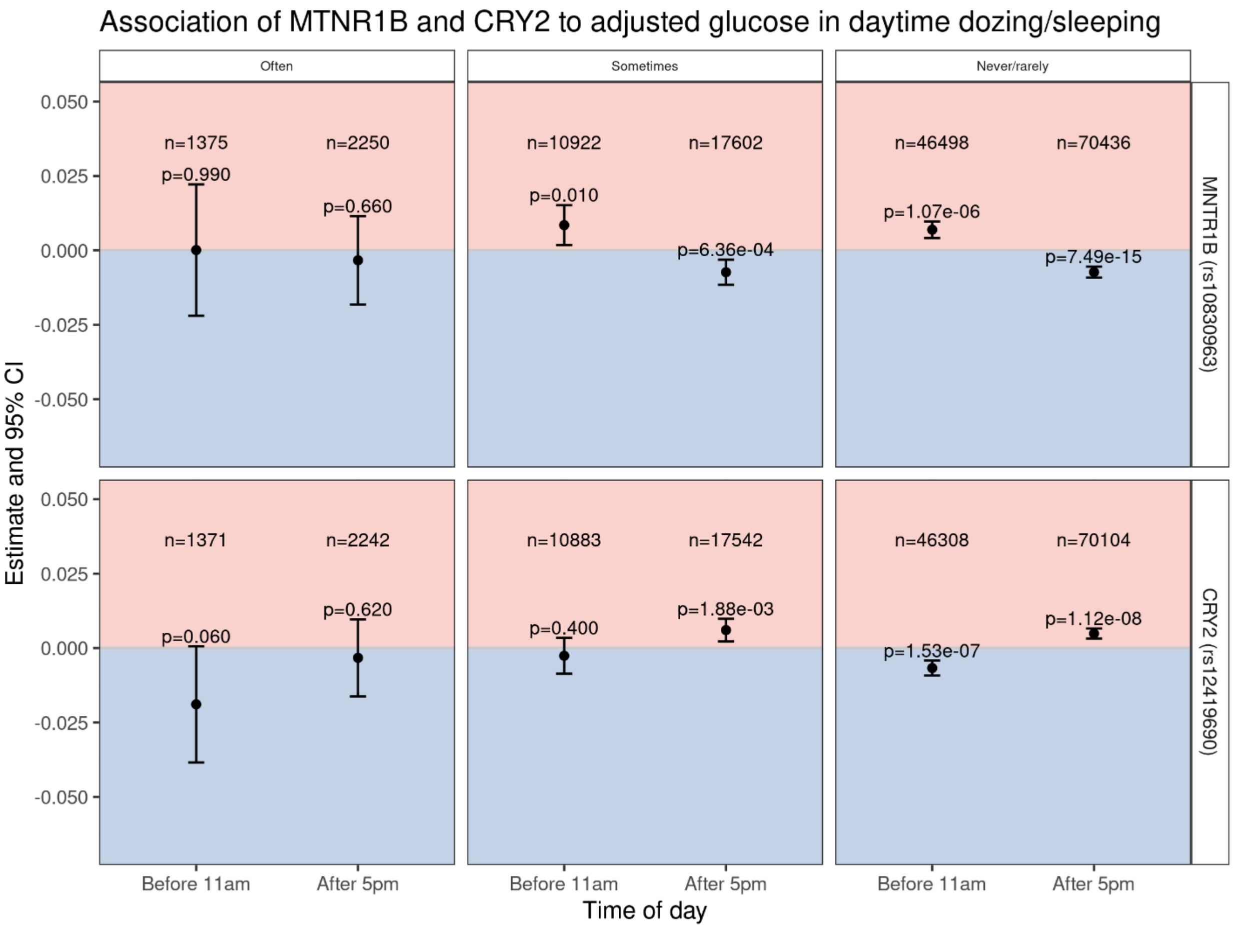

Supplementary Figure 18. Comparison of the association of residual glucose levels in the morning and evening stratified by daytime dozing/sleeping. We computed the effect size for *CRY2* and *MTNR1B* risk alleles, stratified by time of day of sampling and daytime dozing/sleeping (as given in UKBB questionnaire data). Data is presented as additive genotype effect size and 95% confidence intervals.

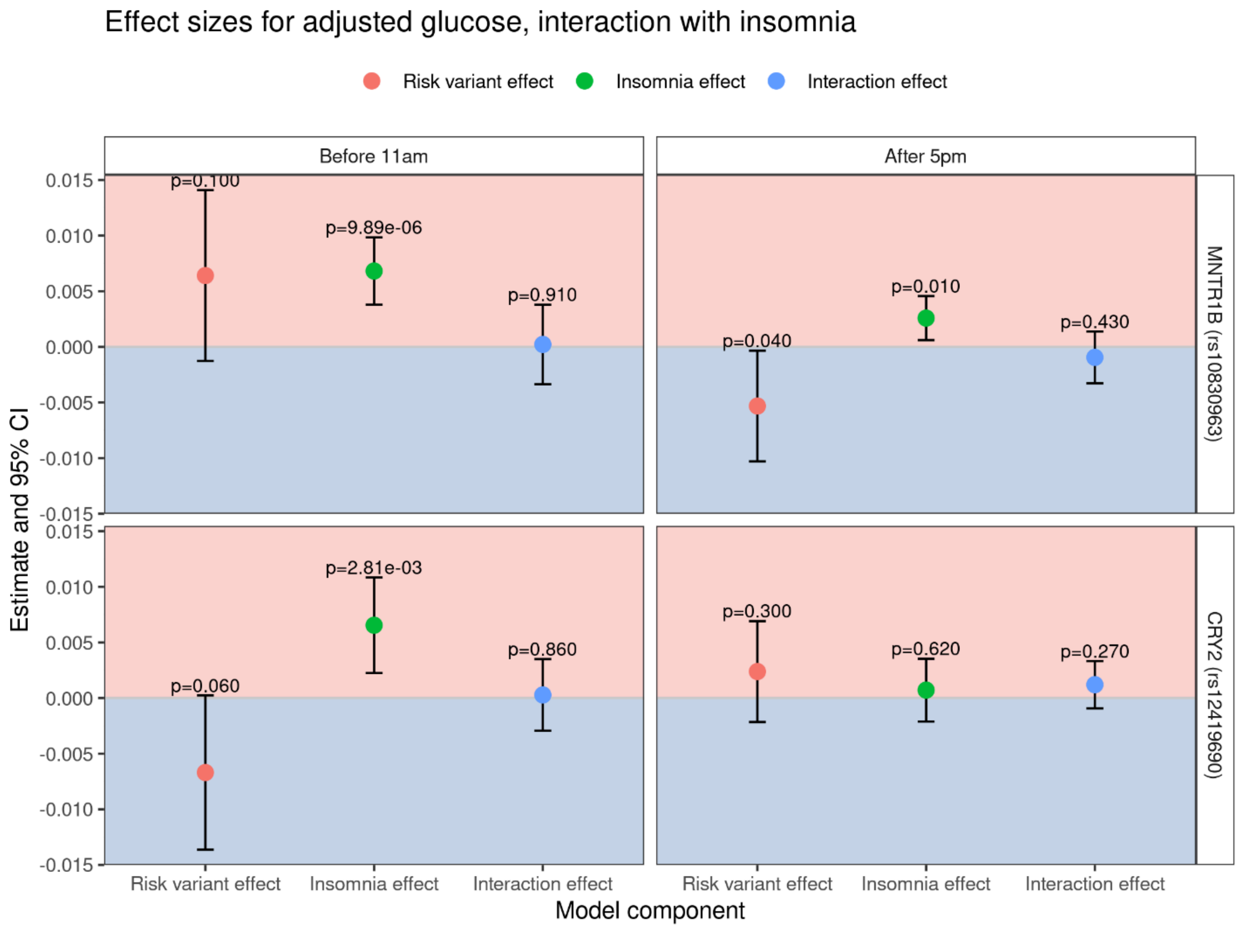

Supplementary Figure 19. Effect of insomnia, risk genotype and interaction between insomnia and genotype on glucose levels. We computed the association of insomnia (green), risk variant (red) and interaction effect (blue) on glucose levels. This shows an effect of insomnia on glucose both in the morning and evening but no interaction between the genotype and insomnia effect. Data is presented as effect size and 95% confidence intervals.

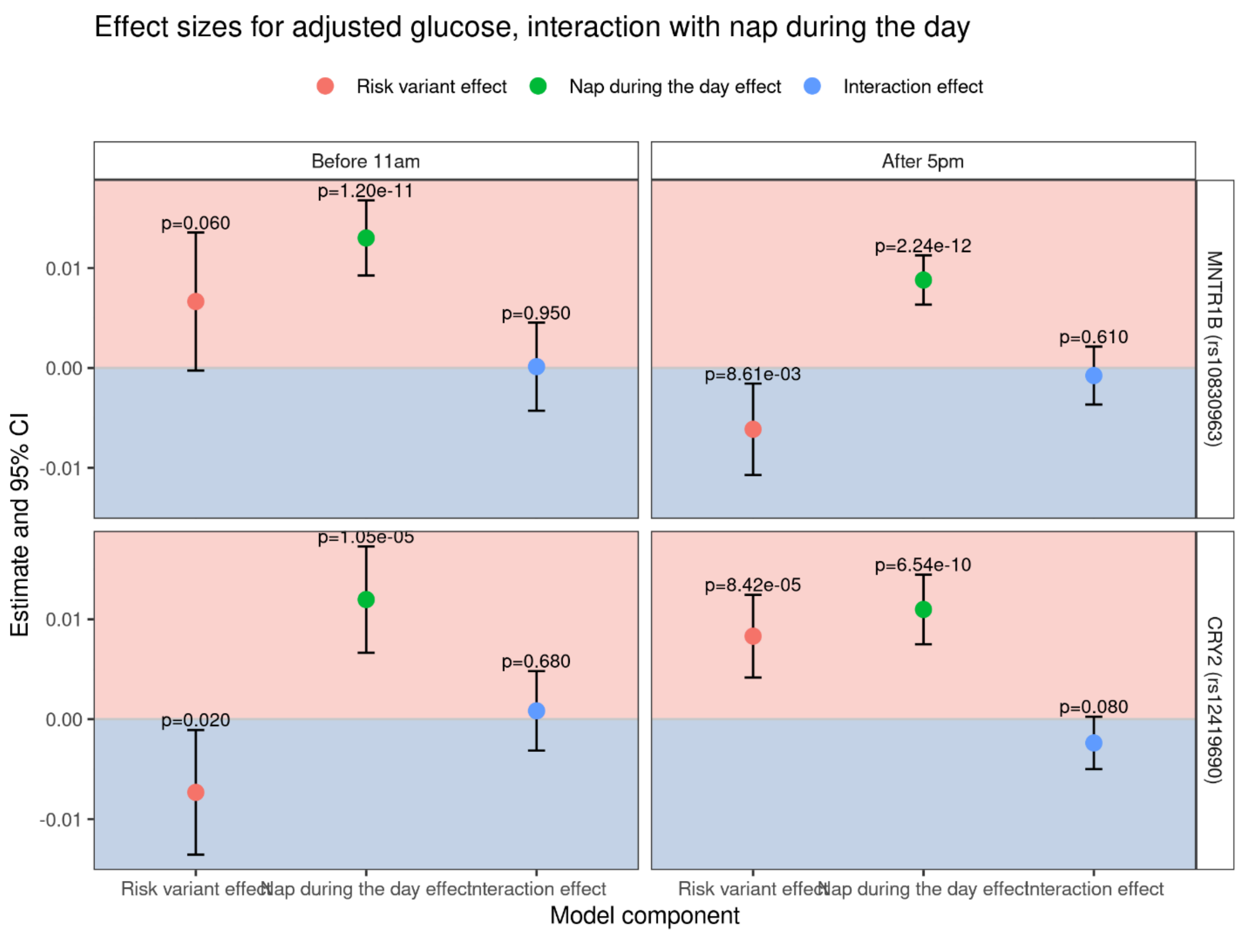

Supplementary Figure 20. Effect of napping, risk genotype and interaction between napping and genotype on glucose levels. We computed the association of nap during the day (green), risk variant (red) and interaction effect (blue) on glucose levels. This shows an effect of napping on glucose both in the morning and evening but no interaction between the genotype and napping effect. Data is presented as effect size and 95% confidence intervals.

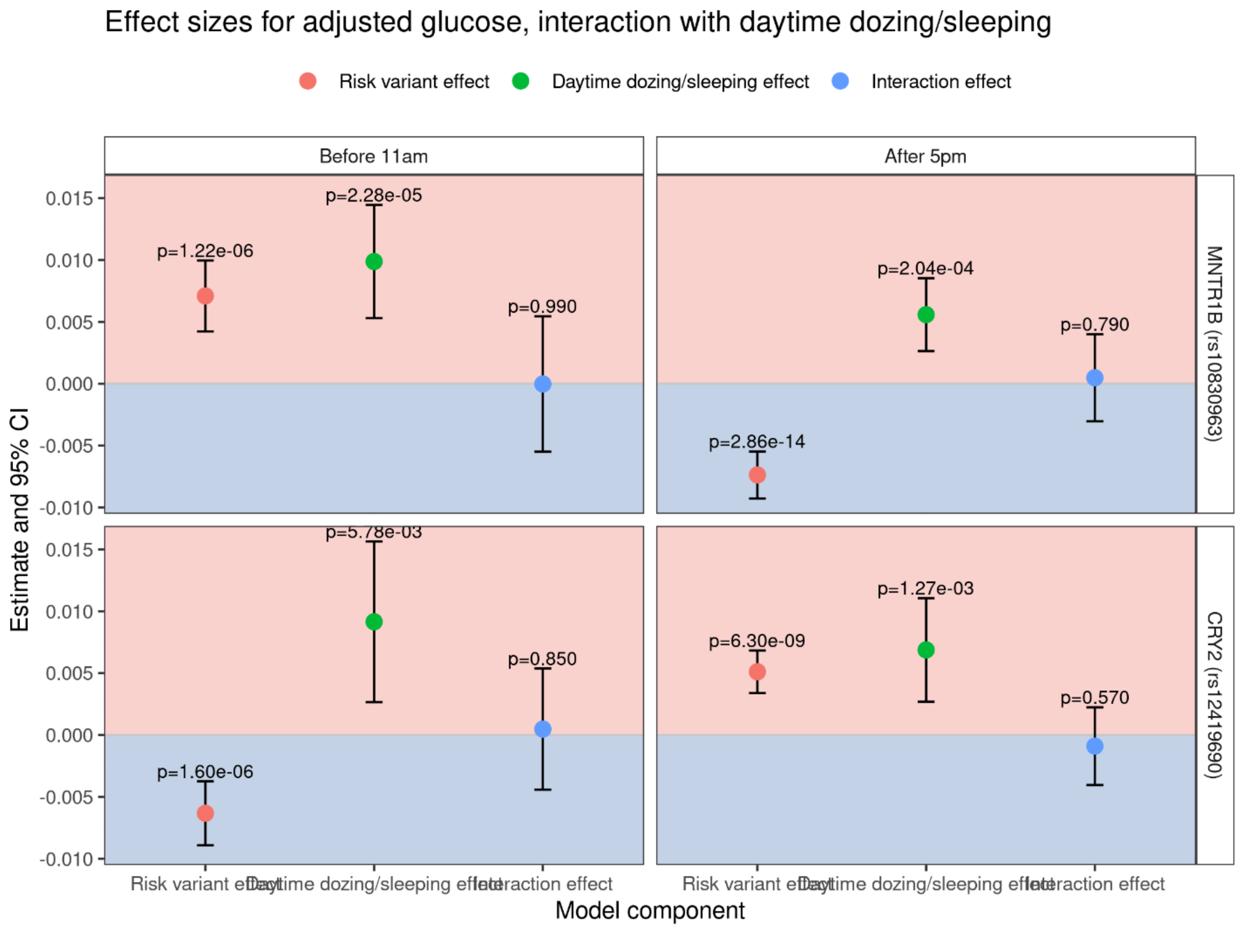

Supplementary Figure 21. Effect of daytime dozing, risk genotype and interaction between daytime dozing and genotype on glucose levels. We computed the association of sleeping/dozing during the day (green), risk variant (red) and interaction effect (blue) on glucose levels. This shows an effect of dozing on glucose both in the morning and evening but no interaction between the genotype and dozing effect. Data is presented as effect size and 95% confidence intervals.

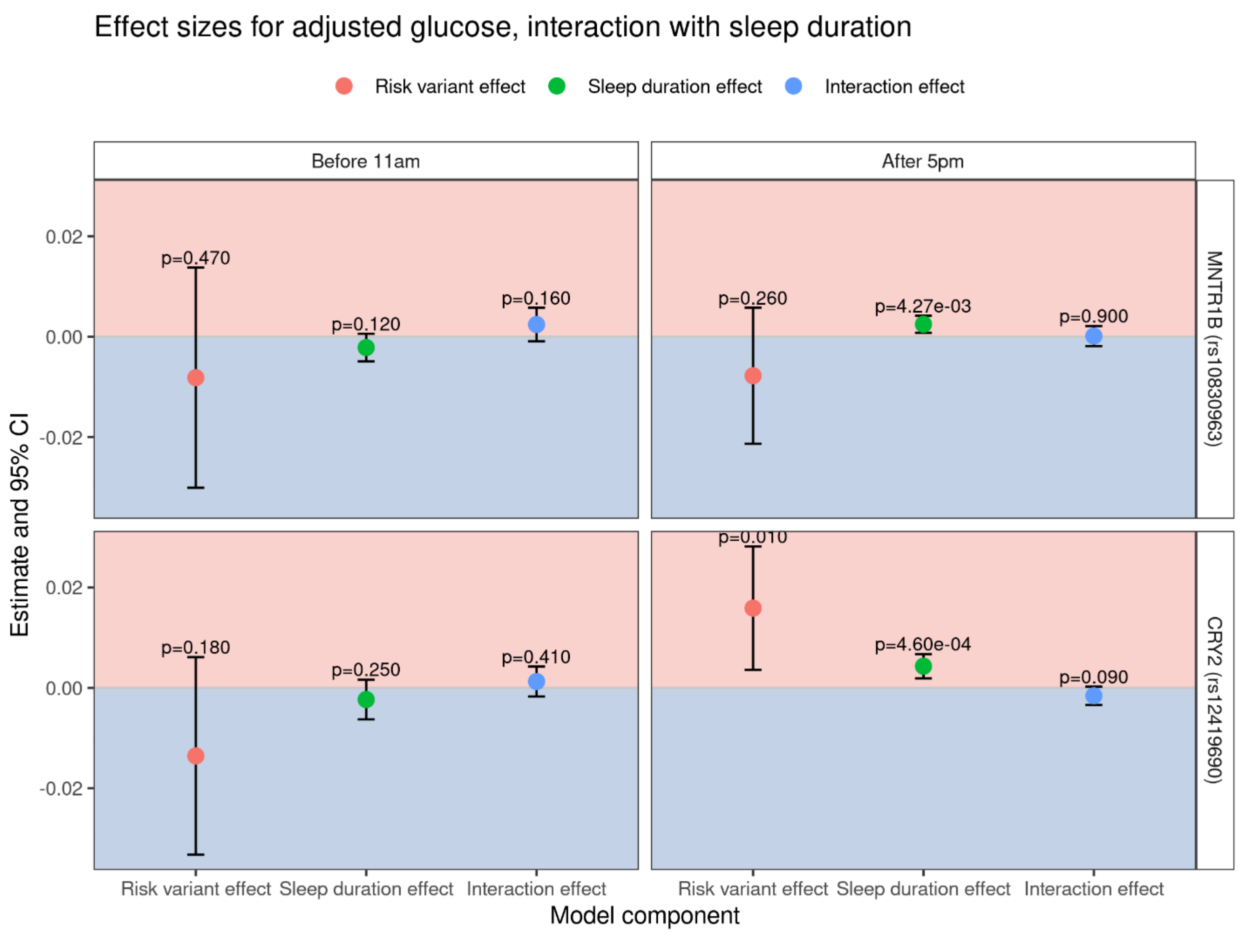

Supplementary Figure 22. Effect of sleep duration, risk genotype and interaction between sleep duration and genotype on glucose levels. We computed the association of sleep duration (green), risk variant (red) and interaction effect (blue) on glucose levels. This shows an effect of sleep duration on glucose in the evening, but no interaction between genotype and sleep duration. Data is presented as effect size and 95% confidence intervals.

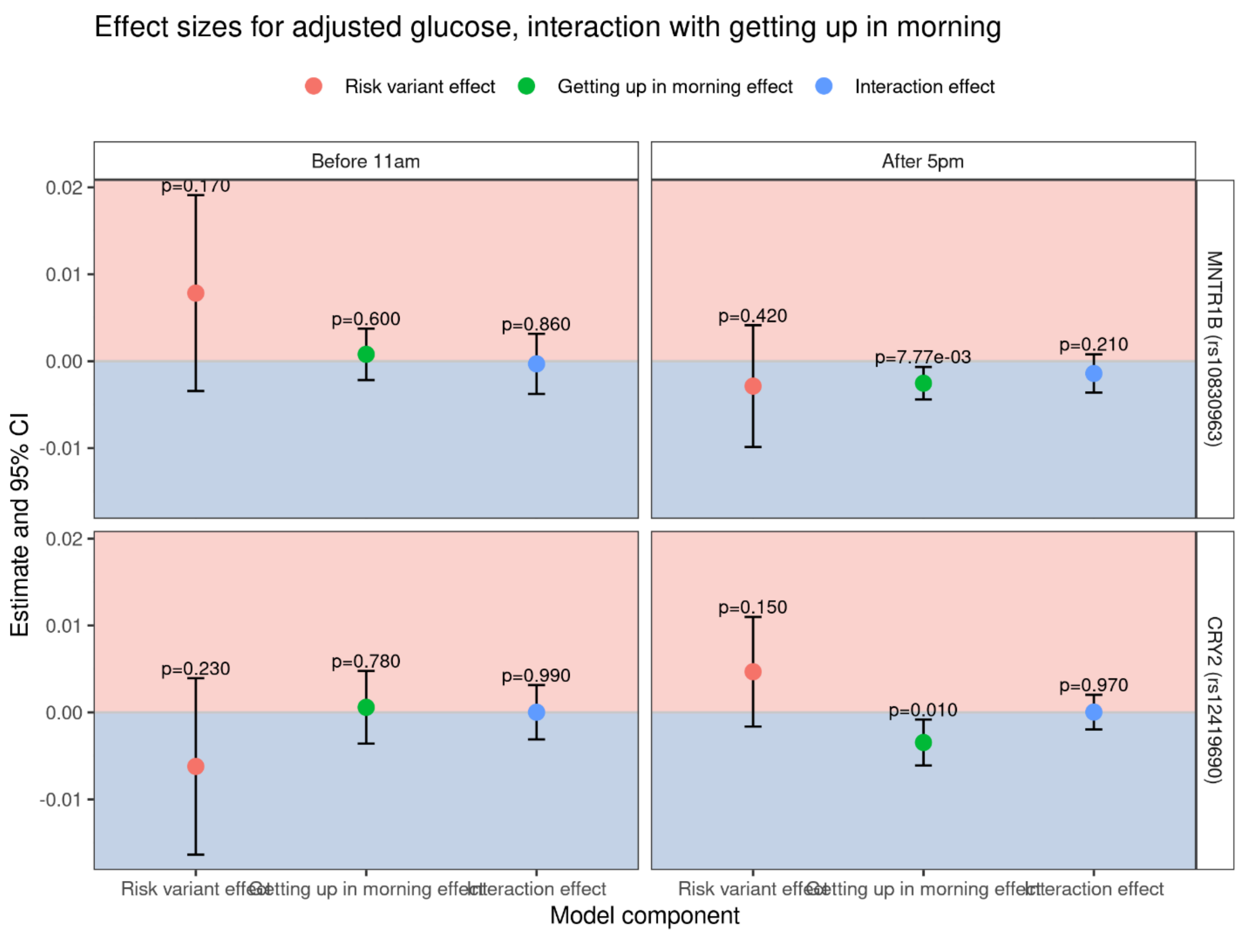

Supplementary Figure 23. Effect of ease of awakening, risk genotype and interaction between ease of awakening and genotype on glucose levels. We computed the association of ease of awakening (green), risk variant (red) and interaction effect (blue) on glucose levels. This shows an effect of ease of awakening on glucose in the evening, but no interaction between the genotype and ease of awakening effect. Data is presented as effect size and 95% confidence intervals.

Supplementary Data 1. (separate file)

Genome-wide association summary statistics for glucose levels in the UK Biobank. P-values represent typical Regenie association summary statistics (main variant effect) for specified glucose phenotypes in the UK Biobank.

Fasting time	Blood collection time	n samples
6 hours or more	Before 11am	12,309
6 hours or more	After 5pm	31,441
8 hours or more	Before 11am	12,133
8 hours or more	After 5pm	3,539
12 hours or more	Before 11am	10,573
12 hours or more	After 5pm	607

Supplementary Table 1.
Number of fasting samples at each blood collection time and fasting time in the UK Biobank.

List of Consortia Members

Estonian Biobank Research Team

Andres Metspalu, Lili Milani, Reedik Mägi, Mari Nelis, Georgi Hudjashov, Tõnu Esko

FinnGen

Full Name	Affiliation	Role 1	Role 2
Aarno Palotie	Institute for Molecular Medicine Finland (FIMM), HiLIFE, University of Helsinki, Helsinki, Finland; Broad Institute of MIT and Harvard; Massachusetts General Hospital	Steering Committee	Steering Committee
Mark Daly	Institute for Molecular Medicine Finland (FIMM), HiLIFE, University of Helsinki, Helsinki, Finland; Broad Institute of MIT and Harvard; Massachusetts General Hospital	Steering Committee	Steering Committee
Bridget Riley-Gills	Abbvie, Chicago, IL, United States	Steering Committee	Pharmaceutical companies
Howard Jacob	Abbvie, Chicago, IL, United States	Steering Committee	Pharmaceutical companies
Neelroop Parikshak	Alnylam Pharmaceuticals, Cambridge, MA, United States	Steering Committee	Pharmaceutical companies
Luke Ward	Alnylam Pharmaceuticals, Cambridge, MA, United States	Steering Committee	Pharmaceutical companies
Coralie Viollet	Astra Zeneca, Cambridge, United Kingdom	Steering Committee	Pharmaceutical companies
Slavé Petrovski	Astra Zeneca, Cambridge, United Kingdom	Steering Committee	Pharmaceutical companies
Alix Berton	Bayer AG, Leverkusen, Germany	Steering Committee	Pharmaceutical companies
Santha Ramakrishnan	Bayer AG, Leverkusen, Germany	Steering Committee	Pharmaceutical companies
Ellen Tsai	Biogen, Cambridge, MA, United States	Steering Committee	Pharmaceutical companies
Zhihao Ding	Boehringer Ingelheim, Ingelheim am Rhein, Germany	Steering Committee	Pharmaceutical companies
Emily Holzinger	Bristol Myers Squibb, New York, NY, United States	Steering Committee	Pharmaceutical companies

Robert Plenge	Bristol Myers Squibb, New York, NY, United States	Steering Committee	Pharmaceutical companies
Joseph Maranville	Bristol Myers Squibb, New York, NY, United States	Steering Committee	Pharmaceutical companies
Mark McCarthy	Genentech, San Francisco, CA, United States	Steering Committee	Pharmaceutical companies
Rion Pendergrass	Genentech, San Francisco, CA, United States	Steering Committee	Pharmaceutical companies
Jonathan Davitte	GlaxoSmithKline, Collegeville, PA, United States	Steering Committee	Pharmaceutical companies
Chia-Yen Chen	Merck, Kenilworth, NJ, United States	Steering Committee	Pharmaceutical companies
Melis Atalar Aksit	Pfizer, New York, NY, United States	Steering Committee	Pharmaceutical companies
Anna Vlahiotis	Pfizer, New York, NY, United States	Steering Committee	Pharmaceutical companies
Katherine Klinger	Translational Sciences, Sanofi R&D, Framingham, MA, USA	Steering Committee	Pharmaceutical companies
Clement Chatelain	Translational Sciences, Sanofi R&D, Framingham, MA, USA	Steering Committee	Pharmaceutical companies
Jorg Blankenstein	Translational Sciences, Sanofi R&D, Framingham, MA, USA	Steering Committee	Pharmaceutical companies
Karol Estrada	Maze Therapeutics, San Francisco, CA, United States	Steering Committee	Pharmaceutical companies
Robert Graham	Maze Therapeutics, San Francisco, CA, United States	Steering Committee	Pharmaceutical companies
Dawn Waterworth	Johnson & Johnson Innovative Medicine, Spring House, PA, United States	Steering Committee	Pharmaceutical companies
Chris O´Donnell	Novartis Institutes for BioMedical Research, Cambridge, MA, United States	Steering Committee	Pharmaceutical companies
Nicole Renaud	Novartis Institutes for BioMedical Research, Cambridge, MA, United States	Steering Committee	Pharmaceutical companies
Tomi P. Mäkelä	HiLIFE, University of Helsinki, Finland, Finland	Steering Committee	University of Helsinki & Biobanks
Jaakko Kaprio	Institute for Molecular Medicine Finland (FIMM), HiLIFE, University of Helsinki, Helsinki, Finland	Steering Committee	University of Helsinki & Biobanks

Minna Ruddock	Arctic biobank / University of Oulu	Steering Committee	University of Helsinki & Biobanks
Lila Kallio	Auria Biobank / University of Turku / Wellbeing Services County of Southwest Finland, Turku, Finland	Steering Committee	University of Helsinki & Biobanks
Antti Hakanen	Auria Biobank / University of Turku / Wellbeing Services County of Southwest Finland, Turku, Finland	Steering Committee	University of Helsinki & Biobanks
Terhi Kilpi	THL Biobank / Finnish Institute for Health and Welfare (THL), Helsinki, Finland	Steering Committee	University of Helsinki & Biobanks
Markus Perola	THL Biobank / Finnish Institute for Health and Welfare (THL), Helsinki, Finland	Steering Committee	University of Helsinki & Biobanks
Jukka Partanen	Finnish Red Cross Blood Service / Finnish Hematology Registry and Clinical Biobank, Helsinki, Finland	Steering Committee	University of Helsinki & Biobanks
Taneli Raivio	Helsinki Biobank / Helsinki University and Hospital District of Helsinki and Uusimaa, Helsinki	Steering Committee	University of Helsinki & Biobanks
Eero Punkka	Helsinki Biobank / Helsinki University and Hospital District of Helsinki and Uusimaa, Helsinki	Steering Committee	University of Helsinki & Biobanks
Teija Kekonen	Northern Finland Biobank Borealis / University of Oulu / Wellbeing services county of North Ostrobothnia, Oulu, Finland	Steering Committee	University of Helsinki & Biobanks
Raisa Serpi	Northern Finland Biobank Borealis / University of Oulu / Wellbeing services county of North Ostrobothnia, Oulu, Finland	Steering Committee	University of Helsinki & Biobanks
Kati Kristiansson	Finnish Clinical Biobank Tampere / University of Tampere / Wellbeing Services County of Pirkanmaa, Tampere, Finland	Steering Committee	University of Helsinki & Biobanks
Sanna Siltanen	Finnish Clinical Biobank Tampere / University of Tampere / Wellbeing Services County of Pirkanmaa, Tampere, Finland	Steering Committee	University of Helsinki & Biobanks
Veli-Matti Kosma	Biobank of Eastern Finland / University of Eastern Finland / Wellbeing services county of North Savo, Kuopio, Finland	Steering Committee	University of Helsinki & Biobanks

Arto Mannermaa	Biobank of Eastern Finland / University of Eastern Finland / Wellbeing services county of North Savo, Kuopio, Finland	Steering Committee	University of Helsinki & Biobanks
Jari Laukkanen	Central Finland Biobank / University of Jyväskylä / Wellbeing Services County of Central Finland, Jyväskylä, Finland	Steering Committee	University of Helsinki & Biobanks
Tiina Jokela	Central Finland Biobank / University of Jyväskylä / Wellbeing Services County of Central Finland, Jyväskylä, Finland	Steering Committee	University of Helsinki & Biobanks
Mervi Ahlroth	Finnish Biobank Cooperative - FINBB	Steering Committee	University of Helsinki & Biobanks
Johanna Mäkelä	Finnish Biobank Cooperative - FINBB	Steering Committee	University of Helsinki & Biobanks
Outi Tuovila	Business Finland, Helsinki, Finland	Steering Committee	Other Experts/ Non-Voting Members
Jeffrey Waring	Abbvie, Chicago, IL, United States	Scientific Committee	Pharmaceutical companies
Bridget Riley-Gillis	Abbvie, Chicago, IL, United States	Scientific Committee	Pharmaceutical companies
Fedik Rahimov	Abbvie, Chicago, IL, United States	Scientific Committee	Pharmaceutical companies
Neelroop Parikshak	Alnylam Pharmaceuticals, Cambridge, MA, United States	Scientific Committee	Pharmaceutical companies
Luke Ward	Alnylam Pharmaceuticals, Cambridge, MA, United States	Scientific Committee	Pharmaceutical companies
Ioanna Tachmazidou	Astra Zeneca, Cambridge, United Kingdom	Scientific Committee	Pharmaceutical companies
Slavé Petrovski	Astra Zeneca, Cambridge, United Kingdom	Scientific Committee	Pharmaceutical companies
Alix Berton	Bayer AG, Leverkusen, Germany	Scientific Committee	Pharmaceutical companies
Santha Ramakrishnan	Bayer AG, Leverkusen, Germany	Scientific Committee	Pharmaceutical companies
Ellen Tsai	Biogen, Cambridge, MA, United States	Scientific Committee	Pharmaceutical companies

Zhihao Ding	Boehringer Ingelheim, Ingelheim am Rhein, Germany	Scientific Committee	Pharmaceutical companies
Marc Jung	Boehringer Ingelheim, Ingelheim am Rhein, Germany	Scientific Committee	Pharmaceutical companies
Hanati Tuoken	Boehringer Ingelheim, Ingelheim am Rhein, Germany	Scientific Committee	Pharmaceutical companies
Shameek Biswas	Bristol Myers Squibb, New York, NY, United States	Scientific Committee	Pharmaceutical companies
Benjamin Sun	Bristol Myers Squibb, New York, NY, United States	Scientific Committee	Pharmaceutical companies
Rion Pendergrass	Genentech, San Francisco, CA, United States	Scientific Committee	Pharmaceutical companies
Jonathan Davitte	GlaxoSmithKline, Collegeville, PA, United States	Scientific Committee	Pharmaceutical companies
Neha Raghavan	Merck, Kenilworth, NJ, United States	Scientific Committee	Pharmaceutical companies
Jae-Hoon Sul	Merck, Kenilworth, NJ, United States	Scientific Committee	Pharmaceutical companies
Melis Atalar Aksit	Pfizer, New York, NY, United States	Scientific Committee	Pharmaceutical companies
Xinli Hu	Pfizer, New York, NY, United States	Scientific Committee	Pharmaceutical companies
Katherine Klinger	Translational Sciences, Sanofi R&D, Framingham, MA, USA	Scientific Committee	Pharmaceutical companies
Robert Graham	Maze Therapeutics, San Francisco, CA, United States	Scientific Committee	Pharmaceutical companies
Dawn Waterworth	Johnson & Johnson Innovative Medicine, Spring House, PA, United States	Scientific Committee	Pharmaceutical companies
Nicole Renaud	Novartis Institutes for BioMedical Research, Cambridge, MA, United States	Scientific Committee	Pharmaceutical companies
Ma´en Obeidat	Novartis Institutes for BioMedical Research, Cambridge, MA, United States	Scientific Committee	Pharmaceutical companies
Jonathan Chung	Novartis Institutes for BioMedical Research, Cambridge, MA, United States	Scientific Committee	Pharmaceutical companies
Jonas Zierer	Novartis Institutes for BioMedical Research, Cambridge, MA, United States	Scientific Committee	Pharmaceutical companies

Mari Niemi	Novartis Institutes for BioMedical Research, Cambridge, MA, United States	Scientific Committee	Pharmaceutical companies
Samuli Ripatti	Institute for Molecular Medicine Finland (FIMM), HiLIFE, University of Helsinki, Helsinki, Finland	Scientific Committee	University of Helsinki & Biobanks
Johanna Schleutker	Auria Biobank / University of Turku / Wellbeing Services County of Southwest Finland, Turku, Finland	Scientific Committee	University of Helsinki & Biobanks
Markus Perola	THL Biobank / Finnish Institute for Health and Welfare (THL), Helsinki, Finland	Scientific Committee	University of Helsinki & Biobanks
Tiina Wahlfors	THL Biobank / Finnish Institute for Health and Welfare (THL), Helsinki, Finland	Scientific Committee	University of Helsinki & Biobanks
Mikko Arvas	Finnish Red Cross Blood Service / Finnish Hematology Registry and Clinical Biobank, Helsinki, Finland	Scientific Committee	University of Helsinki & Biobanks
Olli Carpén	Helsinki Biobank / Helsinki University and Hospital District of Helsinki and Uusimaa, Helsinki	Scientific Committee	University of Helsinki & Biobanks
Reetta Hinttala	Northern Finland Biobank Borealis / University of Oulu / Wellbeing services county of North Ostrobothnia, Oulu, Finland	Scientific Committee	University of Helsinki & Biobanks
Johannes Kettunen	Northern Finland Biobank Borealis / University of Oulu / Wellbeing services county of North Ostrobothnia, Oulu, Finland	Scientific Committee	University of Helsinki & Biobanks
Arto Mannermaa	Biobank of Eastern Finland / University of Eastern Finland / Wellbeing services county of North Savo, Kuopio, Finland	Scientific Committee	University of Helsinki & Biobanks
Katriina Aalto-Setälä	Faculty of Medicine and Health Technology, Tampere University, Tampere, Finland	Scientific Committee	University of Helsinki & Biobanks
Mika Kähönen	Finnish Clinical Biobank Tampere / University of Tampere / Wellbeing Services County of Pirkanmaa, Tampere, Finland	Scientific Committee	University of Helsinki & Biobanks
Jari Laukkanen	Central Finland Biobank / University of Jyväskylä / Wellbeing Services County of Central Finland, Jyväskylä, Finland	Scientific Committee	University of Helsinki & Biobanks

Johanna Mäkelä	FINBB - Finnish biobank cooperative	Scientific Committee	University of Helsinki & Biobanks
Hanna Kujala	Biobank of Eastern Finland / University of Eastern Finland / Wellbeing services county of North Savo, Kuopio, Finland	Clinical Group / Task Force	
Triin Laisk	Estonian biobank, Tartu, Estonia	Clinical Group / Task Force	
Natalia Pujol	Estonian biobank, Tartu, Estonia	Clinical Group / Task Force	
Mika Kähönen	Finnish Clinical Biobank Tampere / University of Tampere / Wellbeing Services County of Pirkanmaa, Tampere, Finland	Clinical Group / Task Force	
Veikko Salomaa	Finnish Institute for Health and Welfare (THL), Helsinki, Finland	Clinical Group / Task Force	
Jaana Suvisaari	Finnish Institute for Health and Welfare (THL), Helsinki, Finland	Clinical Group / Task Force	
Satu Koskela	Finnish Red Cross Blood Service / Finnish Hematology Registry and Clinical Biobank, Helsinki, Finland	Clinical Group / Task Force	
Jouni Lauronen	Finnish Red Cross Blood Service / Finnish Hematology Registry and Clinical Biobank, Helsinki, Finland	Clinical Group / Task Force	
Kristiina Aittomäki	Helsinki University Central Hospital, Helsinki, Finland	Clinical Group / Task Force	
Pirkko Pussinen	Helsinki University Hospital and University of Helsinki, Helsinki / University of Eastern Finland. Kuopio, Finland	Clinical Group / Task Force	
Tuomo Meretoja	Helsinki University Hospital and University of Helsinki, Helsinki, Finland	Clinical Group / Task Force	
Heikki Joensuu	Helsinki University Hospital and University of Helsinki, Helsinki, Finland	Clinical Group / Task Force	

Peeter Karihtala	Helsinki University Hospital and University of Helsinki, Helsinki, Finland	Clinical Group / Task Force	
Emma Juuri	Helsinki University Hospital and University of Helsinki, Helsinki, Finland	Clinical Group / Task Force	
Aino Salminen	Helsinki University Hospital and University of Helsinki, Helsinki, Finland	Clinical Group / Task Force	
Tuula Salo	Helsinki University Hospital and University of Helsinki, Helsinki, Finland	Clinical Group / Task Force	
David Rice	Helsinki University Hospital and University of Helsinki, Helsinki, Finland	Clinical Group / Task Force	
Pekka Nieminen	Helsinki University Hospital and University of Helsinki, Helsinki, Finland	Clinical Group / Task Force	
Ulla Palotie	Helsinki University Hospital and University of Helsinki, Helsinki, Finland	Clinical Group / Task Force	
Fredrik Åberg	Helsinki University Hospital and University of Helsinki, Helsinki, Finland	Clinical Group / Task Force	
Daniel Gordin	Helsinki University Hospital and University of Helsinki, Helsinki, Finland	Clinical Group / Task Force	
Patrik Finne	Helsinki University Hospital and University of Helsinki, Helsinki, Finland	Clinical Group / Task Force	
Joni A Turunen	Helsinki University Hospital and University of Helsinki, Helsinki, Finland; Folkhälsan Research Center, Helsinki, Finland	Clinical Group / Task Force	
Minna Raivio	Hospital District of Helsinki and Uusimaa, Helsinki, Finland	Clinical Group / Task Force	
Pentti Tienari	Hospital District of Helsinki and Uusimaa, Helsinki, Finland	Clinical Group / Task Force	

Martti Färkkilä	Hospital District of Helsinki and Uusimaa, Helsinki, Finland	Clinical Group / Task Force	
Jukka Koskela	Hospital District of Helsinki and Uusimaa, Helsinki, Finland	Clinical Group / Task Force	
Sampsa Pikkarainen	Hospital District of Helsinki and Uusimaa, Helsinki, Finland	Clinical Group / Task Force	
Kari Eklund	Hospital District of Helsinki and Uusimaa, Helsinki, Finland	Clinical Group / Task Force	
Paula Kauppi	Hospital District of Helsinki and Uusimaa, Helsinki, Finland	Clinical Group / Task Force	
Daniel Gordin	Hospital District of Helsinki and Uusimaa, Helsinki, Finland	Clinical Group / Task Force	
Juha Sinisalo	Hospital District of Helsinki and Uusimaa, Helsinki, Finland	Clinical Group / Task Force	
Marja-Riitta Taskinen	Hospital District of Helsinki and Uusimaa, Helsinki, Finland	Clinical Group / Task Force	
Tiinamaija Tuomi	Hospital District of Helsinki and Uusimaa, Helsinki, Finland	Clinical Group / Task Force	
Timo Hiltunen	Hospital District of Helsinki and Uusimaa, Helsinki, Finland	Clinical Group / Task Force	
Johanna Mattson	Hospital District of Helsinki and Uusimaa, Helsinki, Finland	Clinical Group / Task Force	
Eveliina Salminen	Hospital District of Helsinki and Uusimaa, Helsinki, Finland	Clinical Group / Task Force	
Terhi Ollila	Hospital District of Helsinki and Uusimaa, Helsinki, Finland	Clinical Group / Task Force	

Katariina Hannula-Jouppi	Hospital District of Helsinki and Uusimaa, Helsinki, Finland	Clinical Group / Task Force	
Oskari Heikinheimo	Hospital District of Helsinki and Uusimaa, Helsinki, Finland	Clinical Group / Task Force	
Ilkka Kalliala	Hospital District of Helsinki and Uusimaa, Helsinki, Finland	Clinical Group / Task Force	
Lauri Aaltonen	Hospital District of Helsinki and Uusimaa, Helsinki, Finland	Clinical Group / Task Force	
Erkki Isometsä	Hospital District of Helsinki and Uusimaa, Helsinki, Finland	Clinical Group / Task Force	
Antti Aarnisalo	Hospital District of Helsinki and Uusimaa, Helsinki, Finland	Clinical Group / Task Force	
Ilkka Immonen	Hospital District of Helsinki and Uusimaa, Helsinki, Finland	Clinical Group / Task Force	
Salla Ranta	Hospital District of Helsinki and Uusimaa, Helsinki, Finland	Clinical Group / Task Force	
Filip Scheperjans	Hospital District of Helsinki and Uusimaa, Helsinki, Finland	Clinical Group / Task Force	
Felix Vaura	Institute for Molecular Medicine Finland (FIMM), HiLIFE, University of Helsinki, Helsinki, Finland	Clinical Group / Task Force	
Nina Mars	Institute for Molecular Medicine Finland (FIMM), HiLIFE, University of Helsinki, Helsinki, Finland	Clinical Group / Task Force	
Esa Pitkänen	Institute for Molecular Medicine Finland (FIMM), HiLIFE, University of Helsinki, Helsinki, Finland	Clinical Group / Task Force	
Hannele Laivuori	Institute for Molecular Medicine Finland (FIMM), HiLIFE, University of Helsinki, Helsinki, Finland	Clinical Group / Task Force	

Katja Kivinen	Institute for Molecular Medicine Finland (FIMM), HiLIFE, University of Helsinki, Helsinki, Finland	Clinical Group / Task Force	
Elisabeth Widen	Institute for Molecular Medicine Finland (FIMM), HiLIFE, University of Helsinki, Helsinki, Finland	Clinical Group / Task Force	
Taru Tukiainen	Institute for Molecular Medicine Finland (FIMM), HiLIFE, University of Helsinki, Helsinki, Finland	Clinical Group / Task Force	
Hanna Ollila	Institute for Molecular Medicine Finland (FIMM), HiLIFE, University of Helsinki, Helsinki, Finland	Clinical Group / Task Force	
Elmo Saarentaus	Institute for Molecular Medicine Finland (FIMM), HiLIFE, University of Helsinki, Helsinki, Finland	Clinical Group / Task Force	
Anne Kerola	Institute for Molecular Medicine Finland (FIMM), HiLIFE, University of Helsinki, Helsinki, Finland	Clinical Group / Task Force	
Eero Vuoksimaa	Institute for Molecular Medicine Finland (FIMM), HiLIFE, University of Helsinki, Helsinki, Finland	Clinical Group / Task Force	
Joni Lindbohm	Institute for Molecular Medicine Finland (FIMM), HiLIFE, University of Helsinki, Helsinki, Finland	Clinical Group / Task Force	
Zhiyu Yang	Institute for Molecular Medicine Finland (FIMM), HiLIFE, University of Helsinki, Helsinki, Finland	Clinical Group / Task Force	
Matthew Sampson	Institute for Molecular Medicine Finland (FIMM), HiLIFE, University of Helsinki, Helsinki, Finland; Broad Institute & Harvard Medical School, Cambridge, United States	Clinical Group / Task Force	
Adrian Banerji	Institute for Molecular Medicine Finland (FIMM), HiLIFE, University of Helsinki, Helsinki, Finland; Broad Institute & Harvard Medical School, Cambridge, United States	Clinical Group / Task Force	
Michelle McNulty	Institute for Molecular Medicine Finland (FIMM), HiLIFE, University of Helsinki, Helsinki, Finland; Broad Institute & Harvard Medical School, Cambridge, United States	Clinical Group / Task Force	

Aoxing Liu	Institute for Molecular Medicine Finland (FIMM), HiLIFE, University of Helsinki, Helsinki, Finland; Broad Institute, Cambridge, MA, United States	Clinical Group / Task Force	
Joel Rämö	Institute for Molecular Medicine Finland (FIMM), HiLIFE, University of Helsinki, Helsinki, Finland; Broad Institute, Cambridge, MA, United States	Clinical Group / Task Force	
Austin Argentieri	Institute for Molecular Medicine Finland (FIMM), HiLIFE, University of Helsinki, Helsinki, Finland; Broad Institute, Cambridge, MA, United States	Clinical Group / Task Force	
Amanda Elliott	Institute for Molecular Medicine Finland (FIMM), HiLIFE, University of Helsinki, Helsinki, Finland; Broad Institute, Cambridge, MA, USA and Massachusetts General Hospital, Boston, MA, USA	Clinical Group / Task Force	
Elisa Rahikkala	Northern Ostrobothnia Hospital District, Oulu, Finland	Clinical Group / Task Force	
Kirsi Sipilä	Oulu University Hospital and University of Oulu, Oulu, Finland	Clinical Group / Task Force	
Valtteri Julkunen	University of Eastern Finland and Kuopio University Hospital, Kuopio, Finland	Clinical Group / Task Force	
Ville Leinonen	University of Eastern Finland and Kuopio University Hospital, Kuopio, Finland	Clinical Group / Task Force	
Sanna Toppila-Salmi	University of Eastern Finland and Kuopio University Hospital, Kuopio, Finland; Helsinki University Hospital and University of Helsinki, Finland	Clinical Group / Task Force	
Mikko Hiltunen	University of Eastern Finland, Kuopio, Finland	Clinical Group / Task Force	
Eino Solje	University of Eastern Finland, Kuopio, Finland	Clinical Group / Task Force	
Hannu Kankaanranta	University of Gothenburg, Gothenburg, Sweden/ Seinäjoki Central Hospital, Seinäjoki, Finland/ Tampere University, Tampere, Finland	Clinical Group / Task Force	

Antti Mäkitie	University of Helsinki and Helsinki University Hospital, Helsinki, Finland	Clinical Group / Task Force	
Iiris Hovatta	University of Helsinki, Helsinki, Finland	Clinical Group / Task Force	
Niko Välimäki	University of Helsinki, Helsinki, Finland	Clinical Group / Task Force	
Minttu Marttila	University of Helsinki, Helsinki, Finland	Clinical Group / Task Force	
Anne Portaankorva	University of Helsinki, Helsinki, Finland	Clinical Group / Task Force	
Eija Laakkonen	University of Jyväskylä, Jyväskylä, Finland	Clinical Group / Task Force	
Heidi Silven	University of Oulu, Oulu, Finland	Clinical Group / Task Force	
Eeva Sliz	University of Oulu, Oulu, Finland	Clinical Group / Task Force	
Minna Karjalainen	University of Oulu, Oulu, Finland	Clinical Group / Task Force	
Riikka Arffman	University of Oulu, Oulu, Finland	Clinical Group / Task Force	
Susanna Savukoski	University of Oulu, Oulu, Finland	Clinical Group / Task Force	
Riitta Kaarteenaho	University of Oulu, Oulu, Finland	Clinical Group / Task Force	
Jaakko Tyrmi	University of Oulu, Oulu, Finland / University of Tampere, Tampere, Finland	Clinical Group / Task Force	

Laura Kuusalo	University of Turku, Turku, Finland	Clinical Group / Task Force	
Laura Pirilä	University of Turku, Turku, Finland	Clinical Group / Task Force	
Tapio Hellman	University of Turku, Turku, Finland	Clinical Group / Task Force	
Matti Vuori	University of Turku, Turku, Finland	Clinical Group / Task Force	
Teemu Niiranen	University of Turku, Turku, Finland; Finnish Institute for Health and Welfare (THL), Helsinki, Finland	Clinical Group / Task Force	
Timo Blomster	Wellbeing services county of North Ostrobothnia, Oulu, Finland	Clinical Group / Task Force	
Johanna Huhtakangas	Wellbeing services county of North Ostrobothnia, Oulu, Finland	Clinical Group / Task Force	
Terttu Harju	Wellbeing services county of North Ostrobothnia, Oulu, Finland	Clinical Group / Task Force	
Kaisa Tasanen	Wellbeing services county of North Ostrobothnia, Oulu, Finland	Clinical Group / Task Force	
Laura Huilaja	Wellbeing services county of North Ostrobothnia, Oulu, Finland	Clinical Group / Task Force	
Vuokko Anttonen	Wellbeing services county of North Ostrobothnia, Oulu, Finland	Clinical Group / Task Force	
Marja Vääräsmäki	Wellbeing services county of North Ostrobothnia, Oulu, Finland	Clinical Group / Task Force	
Outi Uimari	Wellbeing services county of North Ostrobothnia, Oulu, Finland	Clinical Group / Task Force	

Laure Morin-Papunen	Wellbeing services county of North Ostrobothnia, Oulu, Finland	Clinical Group / Task Force	
Maarit Niinimäki	Wellbeing services county of North Ostrobothnia, Oulu, Finland	Clinical Group / Task Force	
Terhi Piltonen	Wellbeing services county of North Ostrobothnia, Oulu, Finland	Clinical Group / Task Force	
Reetta Kälviäinen	Wellbeing services county of North Savo, Kuopio, Finland	Clinical Group / Task Force	
Valtteri Julkunen	Wellbeing services county of North Savo, Kuopio, Finland	Clinical Group / Task Force	
Hilkka Soininen	Wellbeing services county of North Savo, Kuopio, Finland	Clinical Group / Task Force	
Mikko Kiviniemi	Wellbeing services county of North Savo, Kuopio, Finland	Clinical Group / Task Force	
Oili Kaipiainen-Seppänen	Wellbeing services county of North Savo, Kuopio, Finland	Clinical Group / Task Force	
Margit Pelkonen	Wellbeing services county of North Savo, Kuopio, Finland	Clinical Group / Task Force	
Päivi Auvinen	Wellbeing services county of North Savo, Kuopio, Finland	Clinical Group / Task Force	
Maria Siponen	Wellbeing services county of North Savo, Kuopio, Finland	Clinical Group / Task Force	
Liisa Suominen	Wellbeing services county of North Savo, Kuopio, Finland	Clinical Group / Task Force	
Päivi Mäntylä	Wellbeing services county of North Savo, Kuopio, Finland	Clinical Group / Task Force	

Kai Kaarniranta	Wellbeing services county of North Savo, Kuopio, Finland; University of Lodz, Lodz, Poland	Clinical Group / Task Force	
Jukka Peltola	Wellbeing Services County of Pirkanmaa, Tampere, Finland	Clinical Group / Task Force	
Airi Jussila	Wellbeing Services County of Pirkanmaa, Tampere, Finland	Clinical Group / Task Force	
Katri Kaukinen	Wellbeing Services County of Pirkanmaa, Tampere, Finland	Clinical Group / Task Force	
Pia Isomäki	Wellbeing Services County of Pirkanmaa, Tampere, Finland	Clinical Group / Task Force	
Jussi Hernesniemi	Wellbeing Services County of Pirkanmaa, Tampere, Finland	Clinical Group / Task Force	
Annika Auranen	Wellbeing Services County of Pirkanmaa, Tampere, Finland	Clinical Group / Task Force	
Hannu Uusitalo	Wellbeing Services County of Pirkanmaa, Tampere, Finland	Clinical Group / Task Force	
Teea Salmi	Wellbeing Services County of Pirkanmaa, Tampere, Finland	Clinical Group / Task Force	
Venla Kurra	Wellbeing Services County of Pirkanmaa, Tampere, Finland	Clinical Group / Task Force	
Laura Kotaniemi-Talonen	Wellbeing Services County of Pirkanmaa, Tampere, Finland	Clinical Group / Task Force	
Argyro Bizaki-Vallaskangas	Wellbeing Services County of Pirkanmaa, Tampere, Finland	Clinical Group / Task Force	
Juha Rinne	Wellbeing Services County of Southwest Finland, Turku, Finland	Clinical Group / Task Force	

Roosa Kallionpää	Wellbeing Services County of Southwest Finland, Turku, Finland	Clinical Group / Task Force	
Markku Voutilainen	Wellbeing Services County of Southwest Finland, Turku, Finland	Clinical Group / Task Force	
Antti Palomäki	Wellbeing Services County of Southwest Finland, Turku, Finland	Clinical Group / Task Force	
Laura Pirilä	Wellbeing Services County of Southwest Finland, Turku, Finland	Clinical Group / Task Force	
Riitta Lahesmaa	Wellbeing Services County of Southwest Finland, Turku, Finland	Clinical Group / Task Force	
Kaj Metsärinne	Wellbeing Services County of Southwest Finland, Turku, Finland	Clinical Group / Task Force	
Jenni Aittokallio	Wellbeing Services County of Southwest Finland, Turku, Finland	Clinical Group / Task Force	
Klaus Elenius	Wellbeing Services County of Southwest Finland, Turku, Finland	Clinical Group / Task Force	
Sirkku Peltonen	Wellbeing Services County of Southwest Finland, Turku, Finland	Clinical Group / Task Force	
Leena Koulu	Wellbeing Services County of Southwest Finland, Turku, Finland	Clinical Group / Task Force	
Ulvi Gursoy	Wellbeing Services County of Southwest Finland, Turku, Finland	Clinical Group / Task Force	
Varpu Jokimaa	Wellbeing Services County of Southwest Finland, Turku, Finland	Clinical Group / Task Force	
Tytti Willberg	Wellbeing Services County of Southwest Finland, Turku, Finland	Clinical Group / Task Force	

Adam Ziemann	Abbvie, Chicago, IL, United States	Clinical Group / Task Force	
Nizar Smaoui	Abbvie, Chicago, IL, United States	Clinical Group / Task Force	
Anne Lehtonen	Abbvie, Chicago, IL, United States	Clinical Group / Task Force	
Apinya Lertratanakul	Abbvie, Chicago, IL, United States	Clinical Group / Task Force	
Relja Popovic	Abbvie, Chicago, IL, United States	Clinical Group / Task Force	
Mengzhen Liu	Abbvie, Chicago, IL, United States	Clinical Group / Task Force	
Anneke Den Hollander	AbbVie, Chicago, IL, United States	Clinical Group / Task Force	
Jan Freudenberg	AbbVie, Chicago, IL, United States	Clinical Group / Task Force	
Britney Milkovich	AbbVie, Chicago, IL, United States	Clinical Group / Task Force	
Andrew Blumenfeld	AbbVie, Chicago, IL, United States	Clinical Group / Task Force	
Tushar Kumar	AbbVie, Chicago, IL, United States	Clinical Group / Task Force	
Neelroop Parikshak	Alnylam Pharmaceuticals, Cambridge, MA, United States	Clinical Group / Task Force	
Rajashree Mishra	Alnylam Pharmaceuticals, Cambridge, MA, United States	Clinical Group / Task Force	

Lynne Krohn	Alnylam Pharmaceuticals, Cambridge, MA, United States	Clinical Group / Task Force	
Vincent Battista	Alnylam Pharmaceuticals, Cambridge, MA, United States	Clinical Group / Task Force	
Dirk Paul	Astra Zeneca, Cambridge, United Kingdom	Clinical Group / Task Force	
Bram Prins	Astra Zeneca, Cambridge, United Kingdom	Clinical Group / Task Force	
Eleanor Wheeler	Astra Zeneca, Cambridge, United Kingdom	Clinical Group / Task Force	
Kousik Kundu	Astra Zeneca, Cambridge, United Kingdom	Clinical Group / Task Force	
Santosh Atanur	Astra Zeneca, Cambridge, United Kingdom	Clinical Group / Task Force	
Andrew Lowe	Astra Zeneca, Cambridge, United Kingdom	Clinical Group / Task Force	
Thomas Spargo	Astra Zeneca, Cambridge, United Kingdom	Clinical Group / Task Force	
Oliver Burren	Astra Zeneca, Cambridge, United Kingdom	Clinical Group / Task Force	
Margarete Fabre	AstraZeneca, Cambridge, United Kingdom	Clinical Group / Task Force	
Fabio Baschiera	Bayer AG, Leverkusen, Germany	Clinical Group / Task Force	
Hans van Leeuwen	Bayer AG, Leverkusen, Germany	Clinical Group / Task Force	

Himanshu Manchanda	Bayer AG, Leverkusen, Germany	Clinical Group / Task Force	
Karl Heilbron	Bayer AG, Leverkusen, Germany	Clinical Group / Task Force	
Kaitlyn Price	Bayer AG, Leverkusen, Germany	Clinical Group / Task Force	
Martin Rao	Bayer AG, Leverkusen, Germany	Clinical Group / Task Force	
Nicole Schmidt	Bayer AG, Leverkusen, Germany	Clinical Group / Task Force	
Samu Kurki	Bayer AG, Leverkusen, Germany	Clinical Group / Task Force	
Johanna Mielke	Bayer AG, Leverkusen, Germany	Clinical Group / Task Force	
Juho Immonen	Bayer AG, Leverkusen, Germany	Clinical Group / Task Force	
Thomas Battram	Bayer AG, Leverkusen, Germany	Clinical Group / Task Force	
Tobias Hogrebe	Bayer AG, Leverkusen, Germany	Clinical Group / Task Force	
Susan Eaton	Biogen, Cambridge, MA, United States	Clinical Group / Task Force	
Ketian Yu	Biogen, Cambridge, MA, United States	Clinical Group / Task Force	
Coro Paisan-Ruiz	Biogen, Cambridge, MA, United States	Clinical Group / Task Force	

Elke Markert	Boehringer Ingelheim, Ingelheim am Rhein, Germany	Clinical Group / Task Force	
Frank Li	Boehringer Ingelheim, Ingelheim am Rhein, Germany	Clinical Group / Task Force	
Yao Hu	Boehringer Ingelheim, Ingelheim am Rhein, Germany	Clinical Group / Task Force	
Christoph Ogris	Boehringer Ingelheim, Ingelheim am Rhein, Germany	Clinical Group / Task Force	
Eric Simon	Boehringer Ingelheim, Ingelheim am Rhein, Germany	Clinical Group / Task Force	
Julio Cesar Bolivar Lopez	Boehringer Ingelheim, Ingelheim am Rhein, Germany	Clinical Group / Task Force	
Monika Frysz	Boehringer Ingelheim, Ingelheim am Rhein, Germany	Clinical Group / Task Force	
Marla Hochfeld	Bristol Myers Squibb, New York, NY, United States	Clinical Group / Task Force	
Cara Carty	Bristol Myers Squibb, New York, NY, United States	Clinical Group / Task Force	
Michael Turchin	Bristol Myers Squibb, New York, NY, United States	Clinical Group / Task Force	
Neelakshi Jog	Bristol Myers Squibb, New York, NY, United States	Clinical Group / Task Force	
Corneliu Bodea	Bristol Myers Squibb, New York, NY, United States	Clinical Group / Task Force	
Janie Shelton	Bristol Myers Squibb, New York, NY, United States	Clinical Group / Task Force	

Chen Li	Bristol Myers Squibb, New York, NY, United States	Clinical Group / Task Force	
Kritika Singh	Bristol Myers Squibb, New York, NY, United States	Clinical Group / Task Force	
Peng Jiang	Bristol Myers Squibb, New York, NY, United States	Clinical Group / Task Force	
Stephanie Loomis	Bristol Myers Squibb, New York, NY, United States	Clinical Group / Task Force	
Elena Sanchez	Bristol Myers Squibb, New York, NY, United States	Clinical Group / Task Force	
Lilith Moss	Bristol Myers Squibb, New York, NY, United States	Clinical Group / Task Force	
Zijie Zhao	Bristol Myers Squibb, New York, NY, United States	Clinical Group / Task Force	
Anna Podgornaia	Bristol Myers Squibb, New York, NY, United States	Clinical Group / Task Force	
Natalie Bowers	Genentech, San Francisco, CA, United States	Clinical Group / Task Force	
Edmond Teng	Genentech, San Francisco, CA, United States	Clinical Group / Task Force	
Tim Lu	Genentech, San Francisco, CA, United States	Clinical Group / Task Force	
Hubert Chen	Genentech, San Francisco, CA, United States	Clinical Group / Task Force	
Jennifer Schutzman	Genentech, San Francisco, CA, United States	Clinical Group / Task Force	

Erich Strauss	Genentech, San Francisco, CA, United States	Clinical Group / Task Force	
Hao Chen	Genentech, San Francisco, CA, United States	Clinical Group / Task Force	
David Choy	Genentech, San Francisco, CA, United States	Clinical Group / Task Force	
Rion Pendergrass	Genentech, San Francisco, CA, United States	Clinical Group / Task Force	
Brian Yaspan	Genentech, San Francisco, CA, United States	Clinical Group / Task Force	
Cameron Adams	Genentech, San Francisco, CA, United States	Clinical Group / Task Force	
Mark McCarthy	Genentech, San Francisco, CA, United States	Clinical Group / Task Force	
Michael Rothenberg	Genentech, San Francisco, CA, United States	Clinical Group / Task Force	
Rion Pendergrass	Genentech, San Francisco, CA, United States	Clinical Group / Task Force	
Sergio Dellepiane	Genentech, San Francisco, CA, United States	Clinical Group / Task Force	
Anubha Mahajan	Genentech, San Francisco, CA, United States	Clinical Group / Task Force	
Michael Holmes	Genentech, San Francisco, CA, United States	Clinical Group / Task Force	
Anubha Mahajan	Genentech, San Francisco, CA, United States	Clinical Group / Task Force	

Diana Chang	Genentech, San Francisco, CA, United States	Clinical Group / Task Force	
Tushar Bhangale	Genentech, San Francisco, CA, United States	Clinical Group / Task Force	
Fanli Xu	GlaxoSmithKline, Brentford, United Kingdom	Clinical Group / Task Force	
Laura Addis	GlaxoSmithKline, Brentford, United Kingdom	Clinical Group / Task Force	
John Eicher	GlaxoSmithKline, Brentford, United Kingdom	Clinical Group / Task Force	
Linda McCarthy	GlaxoSmithKline, Brentford, United Kingdom	Clinical Group / Task Force	
Jorge Esparza Gordillo	GlaxoSmithKline, Brentford, United Kingdom	Clinical Group / Task Force	
Joanna Betts	GlaxoSmithKline, Brentford, United Kingdom	Clinical Group / Task Force	
Audrey Chu	GlaxoSmithKline, Brentford, United Kingdom	Clinical Group / Task Force	
Diptee Kulkarni	GlaxoSmithKline, Brentford, United Kingdom	Clinical Group / Task Force	
Janet Kumar	GlaxoSmithKline, Collegeville, PA, United States	Clinical Group / Task Force	
Charli Harlow	GlaxoSmithKline, Collegeville, PA, United States	Clinical Group / Task Force	
Lea Sarow-Blat	GlaxoSmithKline, Collegeville, PA, United States	Clinical Group / Task Force	

Diana L.Cousminer	GlaxoSmithKline, Collegeville, PA, United States	Clinical Group / Task Force	
Jagtar Nijjar	GlaxoSmithKline, Collegeville, PA, United States	Clinical Group / Task Force	
Jessica Chao	GlaxoSmithKline, Collegeville, PA, United States	Clinical Group / Task Force	
Michal Magid	GlaxoSmithKline, Collegeville, PA, United States	Clinical Group / Task Force	
Shashank Jariwala	GlaxoSmithKline, Collegeville, PA, United States	Clinical Group / Task Force	
Chris Floyd	GlaxoSmithKline, Collegeville, PA, United States	Clinical Group / Task Force	
Dan Swerdlow	GlaxoSmithKline, Collegeville, PA, United States	Clinical Group / Task Force	
Erding Hu	GlaxoSmithKline, Collegeville, PA, United States	Clinical Group / Task Force	
Prerak Desai	GlaxoSmithKline, Collegeville, PA, United States	Clinical Group / Task Force	
Stephen Haddad	GlaxoSmithKline, Collegeville, PA, United States	Clinical Group / Task Force	
Damien Croteau-Chonka	GlaxoSmithKline, Collegeville, PA, United States	Clinical Group / Task Force	
Billy Fahy	GlaxoSmithKline, Collegeville, PA, United States	Clinical Group / Task Force	
Paola Bronson	GlaxoSmithKline, Collegeville, PA, United States	Clinical Group / Task Force	

Kirsi Auro	GlaxoSmithKline, Espoo, Finland	Clinical Group / Task Force	
David Pulford	GlaxoSmithKline, Stevenage, United Kingdom	Clinical Group / Task Force	
Sauli Vuoti	Janssen-Cilag Oy, Espoo, Finland	Clinical Group / Task Force	
Dermot Reilly	Johnson & Johnson Innovative Medicine, Boston, MA, United States	Clinical Group / Task Force	
Karen He	Johnson & Johnson Innovative Medicine, Spring House, PA, United States	Clinical Group / Task Force	
Ekaterina Khramtsova	Johnson & Johnson Innovative Medicine, Spring House, PA, United States	Clinical Group / Task Force	
Amy Hart	Johnson & Johnson Innovative Medicine, Spring House, PA, United States	Clinical Group / Task Force	
Meijian Guan	Johnson & Johnson Innovative Medicine, Spring House, PA, United States	Clinical Group / Task Force	
Alessandro Porello	Johnson & Johnson Innovative Medicine, Spring House, PA, United States	Clinical Group / Task Force	
P. Dunnmon	Johnson & Johnson Innovative Medicine, Spring House, PA, United States	Clinical Group / Task Force	
Sara Gale	Johnson & Johnson Innovative Medicine, Spring House, PA, United States	Clinical Group / Task Force	
Brice Keyes	Johnson & Johnson Innovative Medicine, Spring House, PA, United States	Clinical Group / Task Force	
John Kwon	Johnson & Johnson Innovative Medicine, Spring House, PA, United States	Clinical Group / Task Force	

Jonathan Sherlock	Johnson & Johnson Innovative Medicine, Spring House, PA, United States	Clinical Group / Task Force	
Matt Loza	Johnson & Johnson Innovative Medicine, Spring House, PA, United States	Clinical Group / Task Force	
Chris Whelan	Johnson & Johnson Innovative Medicine, Spring House, PA, United States	Clinical Group / Task Force	
W Galpern	Johnson & Johnson Innovative Medicine, Spring House, PA, United States	Clinical Group / Task Force	
Yanfei Zhang	Johnson & Johnson Innovative Medicine, Spring House, PA, United States	Clinical Group / Task Force	
Mona Selej	Johnson & Johnson Innovative Medicine, Spring House, PA, United States	Clinical Group / Task Force	
Abolfazl Doostparast Torshizi	Johnson & Johnson Innovative Medicine, Spring House, PA, United States	Clinical Group / Task Force	
Qingqin S Li	Johnson & Johnson Innovative Medicine, Titusville, NJ, United States	Clinical Group / Task Force	
Sahar Mozzafari	Maze Therapeutics, San Francisco, CA, United States	Clinical Group / Task Force	
Christopher Deboever	Maze Therapeutics, San Francisco, CA, United States	Clinical Group / Task Force	
Jason Miller	Merck, Kenilworth, NJ, United States	Clinical Group / Task Force	
Fabiana Farias	Merck, Kenilworth, NJ, United States	Clinical Group / Task Force	
Andrey Loboda	Merck, Kenilworth, NJ, United States	Clinical Group / Task Force	

Andrew Stiemke	Merck, Kenilworth, NJ, United States	Clinical Group / Task Force	
Jorge Del-aguila	Merck, Kenilworth, NJ, United States	Clinical Group / Task Force	
Elisabeth Vollmann	Merck, Kenilworth, NJ, United States	Clinical Group / Task Force	
Jozsef Karman	Merck, Kenilworth, NJ, United States	Clinical Group / Task Force	
Julie Fiore	Merck, Kenilworth, NJ, United States	Clinical Group / Task Force	
Rajesh Kamath	Merck, Kenilworth, NJ, United States	Clinical Group / Task Force	
Andrei Popescu	Merck, Kenilworth, NJ, United States	Clinical Group / Task Force	
Delphine Fagegaltier	Merck, Kenilworth, NJ, United States	Clinical Group / Task Force	
Travis Barr	Merck, Kenilworth, NJ, United States	Clinical Group / Task Force	
Aristide Merola	Merck, Kenilworth, NJ, United States	Clinical Group / Task Force	
Oliver Freeman	Merck, Kenilworth, NJ, United States	Clinical Group / Task Force	
Simonne Longerich	Merck, Kenilworth, NJ, United States	Clinical Group / Task Force	
Enrico Ferrero	Novartis Institutes for BioMedical Research, Cambridge, MA, United States	Clinical Group / Task Force	

Nikos Patsopoulos	Novartis Institutes for BioMedical Research, Cambridge, MA, United States	Clinical Group / Task Force	
Nancy Finkel	Novartis Institutes for BioMedical Research, Cambridge, MA, United States	Clinical Group / Task Force	
Sabina Pfister	Novartis Institutes for BioMedical Research, Cambridge, MA, United States	Clinical Group / Task Force	
Shola Richards	Novartis Institutes for BioMedical Research, Cambridge, MA, United States	Clinical Group / Task Force	
Katherine Mccauley	Novartis Institutes for BioMedical Research, Cambridge, MA, United States	Clinical Group / Task Force	
Xiaobo Xia	Novartis Institutes for BioMedical Research, Cambridge, MA, United States	Clinical Group / Task Force	
Mike Mendelson	Novartis Institutes for BioMedical Research, Cambridge, MA, United States	Clinical Group / Task Force	
Majd Mouded	Novartis, Basel, Switzerland	Clinical Group / Task Force	
Debby Ngo	Novartis, Basel, Switzerland	Clinical Group / Task Force	
Kirsi Kalpala	Pfizer, New York, NY, United States	Clinical Group / Task Force	
Melissa Miller	Pfizer, New York, NY, United States	Clinical Group / Task Force	
Nan Bing	Pfizer, New York, NY, United States	Clinical Group / Task Force	
Jaakko Parkkinen	Pfizer, New York, NY, United States	Clinical Group / Task Force	

Heli Lehtonen	Pfizer, New York, NY, United States	Clinical Group / Task Force	
Stefan McDonough	Pfizer, New York, NY, United States	Clinical Group / Task Force	
Ying Wu	Pfizer, New York, NY, United States	Clinical Group / Task Force	
Erin Macdonald-Dunlop	Pfizer, New York, NY, United States	Clinical Group / Task Force	
Shih-Feng You	Pfizer, New York, NY, United States	Clinical Group / Task Force	
Leon Tejwani	Pfizer, New York, NY, United States	Clinical Group / Task Force	
Jessica Chung	Pfizer, New York, NY, United States	Clinical Group / Task Force	
Michael McLean	Pfizer, New York, NY, United States	Clinical Group / Task Force	
Joshua Chiou	Pfizer, New York, NY, United States	Clinical Group / Task Force	
Hye In Kim	Pfizer, New York, NY, United States	Clinical Group / Task Force	
Sivakumar Pitchumani	Pfizer, New York, NY, United States	Clinical Group / Task Force	
Sumedha Jassal	Pfizer, New York, NY, United States	Clinical Group / Task Force	
Madhurima Saxena	Pfizer, New York, NY, United States	Clinical Group / Task Force	

Katherine Knutson	Pfizer, New York, NY, United States	Clinical Group / Task Force	
Lindsay King	Pfizer, New York, NY, United States	Clinical Group / Task Force	
Shunjie Guan	Pfizer, New York, NY, United States	Clinical Group / Task Force	
Zhan Ye	Pfizer, New York, NY, United States	Clinical Group / Task Force	
Catherine O’Riordan	Translational Sciences, Sanofi R&D, Framingham, MA, USA	Clinical Group / Task Force	
Samuel Lessard	Translational Sciences, Sanofi R&D, Framingham, MA, USA	Clinical Group / Task Force	
Suzanne Jacobs	Translational Sciences, Sanofi R&D, Framingham, MA, USA	Clinical Group / Task Force	
Hamid Mattoo	Translational Sciences, Sanofi R&D, Framingham, MA, USA	Clinical Group / Task Force	
David Habiel	Translational Sciences, Sanofi R&D, Framingham, MA, USA	Clinical Group / Task Force	
Guanling Huan	Translational Sciences, Sanofi R&D, Framingham, MA, USA	Clinical Group / Task Force	
Lila Kallio	Auria Biobank / University of Turku / Wellbeing Services County of Southwest Finland, Turku, Finland	Biobank directors	
Tiina Wahlfors	THL Biobank / Finnish Institute for Health and Welfare (THL), Helsinki, Finland	Biobank directors	
Jukka Partanen	Finnish Red Cross Blood Service / Finnish Hematology Registry and Clinical Biobank, Helsinki, Finland	Biobank directors	

Eero Punkka	Helsinki Biobank / Helsinki University and Hospital District of Helsinki and Uusimaa, Helsinki	Biobank directors	
Raisa Serpi	Northern Finland Biobank Borealis / University of Oulu / Wellbeing services county of North Ostrobothnia, Oulu, Finland	Biobank directors	
Sanna Siltanen	Finnish Clinical Biobank Tampere / University of Tampere / Wellbeing Services County of Pirkanmaa, Tampere, Finland	Biobank directors	
Veli-Matti Kosma	Biobank of Eastern Finland / University of Eastern Finland / Wellbeing services county of North Savo, Kuopio, Finland	Biobank directors	
Tiina Jokela	Central Finland Biobank / University of Jyväskylä / Wellbeing Services County of Central Finland, Jyväskylä, Finland	Biobank directors	
Anu Jalanko	Institute for Molecular Medicine Finland (FIMM), HiLIFE, University of Helsinki, Helsinki, Finland	FinnGen Teams	Administration
Risto Kajanne	Institute for Molecular Medicine Finland (FIMM), HiLIFE, University of Helsinki, Helsinki, Finland	FinnGen Teams	Administration
Mervi Aavikko	Institute for Molecular Medicine Finland (FIMM), HiLIFE, University of Helsinki, Helsinki, Finland	FinnGen Teams	Administration
Helen Cooper	Institute for Molecular Medicine Finland (FIMM), HiLIFE, University of Helsinki, Helsinki, Finland	FinnGen Teams	Administration
Denise Öller	Institute for Molecular Medicine Finland (FIMM), HiLIFE, University of Helsinki, Helsinki, Finland	FinnGen Teams	Administration
Tarja Laitinen	Institute for Molecular Medicine Finland (FIMM), HiLIFE, University of Helsinki, Helsinki, Finland	FinnGen Teams	Administration
Sofia Kuitunen	University of Helsinki, Helsinki, Finland	FinnGen Teams	Administration
Auli Toivola	Institute for Molecular Medicine Finland (FIMM), HiLIFE, University of Helsinki, Helsinki, Finland	FinnGen Teams	Sample and data logistics
Rodos Rodosthenous	Institute for Molecular Medicine Finland (FIMM), HiLIFE, University of Helsinki, Helsinki, Finland	FinnGen Teams	Sample and data logistics
Mitja Kurki	Institute for Molecular Medicine Finland (FIMM), HiLIFE, University of Helsinki, Helsinki, Finland; Broad Institute, Cambridge, MA, United States	FinnGen Teams	Analysis
Juha Karjalainen	Institute for Molecular Medicine Finland (FIMM), HiLIFE, University of Helsinki, Helsinki, Finland	FinnGen Teams	Analysis

Pietro Della Briotta Parolo	Institute for Molecular Medicine Finland (FIMM), HiLIFE, University of Helsinki, Helsinki, Finland	FinnGen Teams	Analysis
Arto Lehisto	Institute for Molecular Medicine Finland (FIMM), HiLIFE, University of Helsinki, Helsinki, Finland	FinnGen Teams	Analysis
Juha Mehtonen	Institute for Molecular Medicine Finland (FIMM), HiLIFE, University of Helsinki, Helsinki, Finland	FinnGen Teams	Analysis
Reza Jabal	Institute for Molecular Medicine Finland (FIMM), HiLIFE, University of Helsinki, Helsinki, Finland; Broad Institute, Cambridge, MA, United States	FinnGen Teams	Analysis
Mutaamba Maasha	Institute for Molecular Medicine Finland (FIMM), HiLIFE, University of Helsinki, Helsinki, Finland; Broad Institute, Cambridge, MA, United States	FinnGen Teams	Analysis
Sanni Ruotsalainen	Institute for Molecular Medicine Finland (FIMM), HiLIFE, University of Helsinki, Helsinki, Finland	FinnGen Teams	Analysis
Samuel Jones	Institute for Molecular Medicine Finland (FIMM), HiLIFE, University of Helsinki, Helsinki, Finland	FinnGen Teams	Analysis
Raymond Walters	Institute for Molecular Medicine Finland (FIMM), HiLIFE, University of Helsinki, Helsinki, Finland; Broad Institute, Cambridge, MA, United States	FinnGen Teams	Analysis
Paavo Häppölä	Institute for Molecular Medicine Finland (FIMM), HiLIFE, University of Helsinki, Helsinki, Finland	FinnGen Teams	Analysis
L. Elisa Lahtela	Institute for Molecular Medicine Finland (FIMM), HiLIFE, University of Helsinki, Helsinki, Finland	FinnGen Teams	Disease Task Forces
Johanna Paltta	Institute for Molecular Medicine Finland (FIMM), HiLIFE, University of Helsinki, Helsinki, Finland; University of Turku, Turku, Finland	FinnGen Teams	Disease Task Forces
Juulia Partanen	Institute for Molecular Medicine Finland, HiLIFE, University of Helsinki, Finland	FinnGen Teams	Disease Task Forces
Olli K Pietiläinen	Institute for Molecular Medicine Finland, HiLIFE, University of Helsinki, Finland	FinnGen Teams	Disease Task Forces
Veera Timonen	Institute for Molecular Medicine Finland, HiLIFE, University of Helsinki, Finland	FinnGen Teams	Disease Task Forces
Linda Ottensmann	Institute for Molecular Medicine Finland, HiLIFE, University of Helsinki, Finland	FinnGen Teams	Disease Task Forces
Mari Kaunisto	Institute for Molecular Medicine Finland (FIMM), HiLIFE, University of Helsinki, Helsinki, Finland	FinnGen Teams	Communication
Elina Kilpeläinen	Institute for Molecular Medicine Finland (FIMM), HiLIFE, University of Helsinki, Helsinki, Finland	FinnGen Teams	Sandbox & Cloud Services

Tianduanyi Wang	Institute for Molecular Medicine Finland (FIMM), HiLIFE, University of Helsinki, Helsinki, Finland	FinnGen Teams	Sandbox & Cloud Services
Timo P. Sipilä	Institute for Molecular Medicine Finland (FIMM), HiLIFE, University of Helsinki, Helsinki, Finland	FinnGen Teams	Sandbox & Cloud Services
Oluwaseun Alexander Dada	Institute for Molecular Medicine Finland (FIMM), HiLIFE, University of Helsinki, Helsinki, Finland	FinnGen Teams	Sandbox & Cloud Services
Awaisa Ghazal	Institute for Molecular Medicine Finland (FIMM), HiLIFE, University of Helsinki, Helsinki, Finland	FinnGen Teams	Sandbox & Cloud Services
Rigbe Weldatsadik	Institute for Molecular Medicine Finland (FIMM), HiLIFE, University of Helsinki, Helsinki, Finland	FinnGen Teams	Sandbox & Cloud Services
Jaska Uimonen	Institute for Molecular Medicine Finland (FIMM), HiLIFE, University of Helsinki, Helsinki, Finland	FinnGen Teams	Sandbox & Cloud Services
Kati Donner	Institute for Molecular Medicine Finland (FIMM), HiLIFE, University of Helsinki, Helsinki, Finland	FinnGen Teams	Genotyping
Anu Loukola	Helsinki Biobank / Helsinki University and Hospital District of Helsinki and Uusimaa, Helsinki	FinnGen Teams	Sample Collection Coordination
Päivi Laiho	THL Biobank / Finnish Institute for Health and Welfare (THL), Helsinki, Finland	FinnGen Teams	Sample Logistics
Susanna Lemmelä	Institute for Molecular Medicine Finland (FIMM), HiLIFE, University of Helsinki, Helsinki, Finland	FinnGen Teams	Registry Data Operations
Teemu Paajanen	THL Biobank / Finnish Institute for Health and Welfare (THL), Helsinki, Finland	FinnGen Teams	Registry Data Operations
Arto Pietilä	THL Biobank / Finnish Institute for Health and Welfare (THL), Helsinki, Finland	FinnGen Teams	Registry Data Operations
Aki Havulinna	THL Biobank / Finnish Institute for Health and Welfare (THL), Helsinki, Finland	FinnGen Teams	Registry Data Operations
Mary Pat Reeve	Institute for Molecular Medicine Finland (FIMM), HiLIFE, University of Helsinki, Helsinki, Finland; Broad Institute, Cambridge, MA, United States	FinnGen Teams	Phenotype team
Shanmukha Sampath Padmanabhuni	Institute for Molecular Medicine Finland (FIMM), HiLIFE, University of Helsinki, Helsinki, Finland	FinnGen Teams	Phenotype team
Harri Siirtola	University of Tampere, Tampere, Finland	FinnGen Teams	Phenotype team

Javier Gracia-Tabuenca	University of Tampere, Tampere, Finland	FinnGen Teams	Phenotype team
Marika Kaakinen	Institute for Molecular Medicine Finland (FIMM), HiLIFE, University of Helsinki, Helsinki, Finland	FinnGen Teams	Phenotype team
Shuang Luo	Institute for Molecular Medicine Finland (FIMM), HiLIFE, University of Helsinki, Helsinki, Finland	FinnGen Teams	Phenotype team
Vincent Llorens	Institute for Molecular Medicine Finland (FIMM), HiLIFE, University of Helsinki, Helsinki, Finland	FinnGen Teams	Phenotype team
Dawit Yohannes	Institute for Molecular Medicine Finland (FIMM), HiLIFE, University of Helsinki, Helsinki, Finland	FinnGen Teams	Phenotype team
Iina Laak	Institute for Molecular Medicine Finland (FIMM), HiLIFE, University of Helsinki, Helsinki, Finland	FinnGen Teams	Data protection officer
Mervi Ahlroth	Finnish Biobank Cooperative - FINBB	FinnGen Teams	FINBB - Finnish biobank cooperative
Johanna Mäkelä	Finnish Biobank Cooperative - FINBB	FinnGen Teams	FINBB - Finnish biobank cooperative
Pauli Wihuri	Finnish Biobank Cooperative - FINBB	FinnGen Teams	FINBB - Finnish biobank cooperative
Tom Southerington	Finnish Biobank Cooperative - FINBB	FinnGen Teams	FINBB - Finnish biobank cooperative
Meri Lähteenmäki	Finnish Biobank Cooperative - FINBB	FinnGen Teams	FINBB - Finnish biobank cooperative

VA Million Veteran Program

VA Million Veteran Program
Core Acknowledgements for Publications
October 2025

MVP Program Office

- Sumitra Muralidhar, Ph.D., Program Director
  US Department of Veterans Affairs, 810 Vermont Avenue NW, Washington, DC 20420
- Jennifer Moser, Ph.D., Associate Director, Scientific Programs
  US Department of Veterans Affairs, 810 Vermont Avenue NW, Washington, DC 20420

- Jennifer E. Deen, B.S., Associate Director, Cohort & Public Relations
  US Department of Veterans Affairs, 810 Vermont Avenue NW, Washington, DC 20420

MVP Steering Committee

- Co-Chair: Philip S. Tsao, Ph.D.
  VA Palo Alto Health Care System, 3801 Miranda Avenue, Palo Alto, CA 94304
- Co-Chair: Sumitra Muralidhar, Ph.D.
  US Department of Veterans Affairs, 810 Vermont Avenue NW, Washington, DC 20420
- J. Michael Gaziano, M.D., M.P.H.
  VA Boston Healthcare System, 150 S. Huntington Avenue, Boston, MA 02130
- Adriana Hung, M.D., M.P.H.,
  VA Tennessee Valley Healthcare System, 1310 24th Avenue, South Nashville, TN 37212
- Dave Oslin, M.D.
  Philadelphia VA Medical Center, 3900 Woodland Avenue, Philadelphia, PA 19104
- Deepak Voora, M.D.
  Durham VA Medical Center, 508 Fulton Street, Durham, NC 27705

MVP Co-Principal Investigators

- J. Michael Gaziano, M.D., M.P.H.
  VA Boston Healthcare System, 150 S. Huntington Avenue, Boston, MA 02130
- Philip S. Tsao, Ph.D.
  VA Palo Alto Health Care System, 3801 Miranda Avenue, Palo Alto, CA 94304

MVP Core Operations

- Jessica V. Brewer, M.P.H., Director, MVP Cohort Operations
  VA Boston Healthcare System, 150 S. Huntington Avenue, Boston, MA 02130
- Mary T. Brophy M.D., M.P.H., Director, VA Central Biorepository
  VA Boston Healthcare System, 150 S. Huntington Avenue, Boston, MA 02130
- Kelly Cho, M.P.H, Ph.D., Director, MVP Phenomics
  VA Boston Healthcare System, 150 S. Huntington Avenue, Boston, MA 02130
- Lori Churby, B.S., Director, MVP Regulatory Affairs
  VA Palo Alto Health Care System, 3801 Miranda Avenue, Palo Alto, CA 94304
- Jacob T. Kean, Ph.D., Acting Director, VA Informatics and Computing Infrastructure (VINCI)
  VA Salt Lake City Health Care System, 500 Foothill Drive, Salt Lake City, UT 84148
- Saiju Pyarajan Ph.D., Director, Data and Computational Sciences
  VA Boston Healthcare System, 150 S. Huntington Avenue, Boston, MA 02130
- Robert Ringer, Pharm.D., Director, VA Albuquerque Central Biorepository
  New Mexico VA Health Care System, 1501 San Pedro Drive SE, Albuquerque, NM 87108

- Luis E. Selva, Ph.D., Director, MVP Biorepository Coordination
  VA Boston Healthcare System, 150 S. Huntington Avenue, Boston, MA 02130
- Shahpoor (Alex) Shayan, M.S., Director, MVP PRE Informatics
  VA Boston Healthcare System, 150 S. Huntington Avenue, Boston, MA 02130
- Brady Stephens, M.S., Principal Investigator, MVP Information Center
  Canandaigua VA Medical Center, 400 Fort Hill Avenue, Canandaigua, NY 14424
- Stacey B. Whitbourne, Ph.D., Director, MVP Cohort Development and Management VA Boston Healthcare System, 150 S. Huntington Avenue, Boston, MA 02130
