# Supplementary material for: Genetic variants affect diurnal glucose levels throughout the day
Source: Nat Commun. 2026 May 22;17:6717. doi: 10.1038/s41467-026-72432-6 (PMC13385737; doi:10.1038/s41467-026-72432-6)
Supplement: Supplementary file 2 — Description of Additional Supplementary Files [file 41467_2026_72432_MOESM2_ESM.pdf]

## Description of Additional Supplementary Files

**Supplementary Data 1**. Genome-wide association summary statistics for glucose levels in the UK Biobank. P-values represent typical Regenie association summary statistics (main variant effect) for specified glucose phenotypes in the UK Biobank.
