# Supplementary material for: Genetic variants affect diurnal glucose levels throughout the day
Source: Nat Commun. 2026 May 22;17:6717. doi: 10.1038/s41467-026-72432-6 (PMC13385737; doi:10.1038/s41467-026-72432-6)
Supplement: Supplementary file 4 — Reporting Summary [file 41467_2026_72432_MOESM4_ESM.pdf]

nature portfolio

Corresponding author(s): Nasa Sinnott-Armstrong
Samuel Jones
Hanna M. Ollila

Last updated by author(s): 03/04/2026

Reporting Summary

Nature Portfolio wishes to improve the reproducibility of the work that we publish. This form provides structure for consistency and transparency in reporting. For further information on Nature Portfolio policies, see our Editorial Policies and the Editorial Policy Checklist.

Statistics

For all statistical analyses, confirm that the following items are present in the figure legend, table legend, main text, or Methods section.

n/a	Confirmed	
☐	☒	The exact sample size (*n*) for each experimental group/condition, given as a discrete number and unit of measurement
☐	☒	A statement on whether measurements were taken from distinct samples or whether the same sample was measured repeatedly
☐	☒	The statistical test(s) used AND whether they are one- or two-sided<br>*Only common tests should be described solely by name; describe more complex techniques in the Methods section.*
☐	☒	A description of all covariates tested
☐	☒	A description of any assumptions or corrections, such as tests of normality and adjustment for multiple comparisons
☐	☒	A full description of the statistical parameters including central tendency (e.g. means) or other basic estimates (e.g. regression coefficient) AND variation (e.g. standard deviation) or associated estimates of uncertainty (e.g. confidence intervals)
☐	☒	For null hypothesis testing, the test statistic (e.g. *F*, *t*, *r*) with confidence intervals, effect sizes, degrees of freedom and *P* value noted<br>*Give P values as exact values whenever suitable.*
☒	☐	For Bayesian analysis, information on the choice of priors and Markov chain Monte Carlo settings
☒	☐	For hierarchical and complex designs, identification of the appropriate level for tests and full reporting of outcomes
☐	☒	Estimates of effect sizes (e.g. Cohen's *d*, Pearson's *r*), indicating how they were calculated

Our web collection on statistics for biologists contains articles on many of the points above.

Software and code

Policy information about availability of computer code

Data collection	Data was previously collected by core teams of researchers in each biobank study.
Data analysis	Data was analyzed with several custom software scripts, the details of which is available in the Methods for the original finding and each replication in other biobanks.

For manuscripts utilizing custom algorithms or software that are central to the research but not yet described in published literature, software must be made available to editors and reviewers. We strongly encourage code deposition in a community repository (e.g. GitHub). See the Nature Portfolio guidelines for submitting code & software for further information.

Data

Policy information about availability of data

All manuscripts must include a data availability statement. This statement should provide the following information, where applicable:

- Accession codes, unique identifiers, or web links for publicly available datasets
- A description of any restrictions on data availability
- For clinical datasets or third party data, please ensure that the statement adheres to our policy

Summary statistics of meta-analysis are shared in GWAS catalog at the time of publication: https://www.ebi.ac.uk/gwas/.

FinnGen: Individual-level data is restricted under Finnish law and GDPR, requiring approval by the national authority Findata in addition to FinnGen partner organisation affiliation. Access is granted only within a secure cloud-based Sandbox environment, with no data export permitted for individual-level data. The

process takes several weeks to months. Fill out this form to begin: https://elomake.helsinki.fi/lomakkeet/97654/lomake.html. Researchers must follow a 1-year exclusivity policy and may not report results derived from fewer than 5 individuals. Contact: finngen-info@helsinki.fi.

UK Biobank: Data is open to researchers globally from academic, charity, government, and commercial organisations, provided research is health-related and in the public interest. Applications are submitted via an online Access Management System (https://ams.ukbiobank.ac.uk/ams/signup), reviewed within 10 working days, and take an average of 15 weeks from submission to data release, after which a Material Transfer Agreement must be signed and access fees paid. Individual-level data must remain within the secure cloud platform (UKB-RAP) and cannot be downloaded. WES/WGS data cannot be downloaded under any circumstances. Contact: access@ukbiobank.ac.uk.

Estonian Biobank: Access is governed by the Estonian Human Genes Research Act and requires ethics approval from the Estonian Committee on Bioethics and Human Research for each project. Data is issued in pseudonymised/coded form only and must be analysed within the University of Tartu's secure SAPU server environment. The process begins with a preliminary inquiry to estbbresearch@ut.ee, which receives a response within 5 business days.

VA Million Veteran Program: Individual-level data is currently restricted to VA-affiliated researchers on approved, federally funded projects, and is governed by VA privacy policy and applicable US federal ethics regulations. Data must remain within the VA firewall and be accessed through the secure GenISIS environment. Summary-level GWAS/PheWAS results are freely available via dbGaP without application https://ftp.ncbi.nlm.nih.gov/dbgap/studies/phs002453/analyses/GIA/. No formal response timeframe is specified. Contact MVPLOI@va.gov for data access enquiries.

Research involving human participants, their data, or biological material

Policy information about studies with human participants or human data. See also policy information about sex, gender (identity/presentation), and sexual orientation and race, ethnicity and racism.

Reporting on sex and gender	Sex was considered as a covariate in all reported analyses. Sex was determined based on genetic inference.
Reporting on race, ethnicity, or other socially relevant groupings	Genetically-determined ancestry was used to stratify replication analyses in the Million Veteran Program, which was necessary to avoid spurious results related to allele frequencies differences in populations. Results for each group are reported in the manuscript.
Population characteristics	Population characteristics of each biobank cohort is included in the Methods section of the manuscript.
Recruitment	Recruitment description for each biobank cohort is included in the Methods section of the manuscript.

Ethics oversight

Detailed ethical statements for each biobank cohort is included in the manuscript and here:

Ethics statement Estonian Biobank
The activities of the EstBB are regulated by the Human Genes Research Act, which was adopted in 2000 specifically for the operations of EstBB. Individual level data analysis in EstBB was carried out under ethical approval 1.1-12/624 from the Estonian Committee on Bioethics and Human Research (Estonian Ministry of Social Affairs), using data according to release application T19 6-7/GI/16001 from the Estonian Biobank.

Ethics statement UK Biobank
The UK Biobank has received approval as a Research Tissue Bank from the North West Multi-centre Research Ethics Committee (MREC) under MREC permits 11/NW/0382 (2011–2016), 16/NW/0274 (2016–2021) and 21/NW/0157 (2021–2026). Researchers with approved applications are covered by these permits and are not required to seek additional approval, except in specific cases (see section B7 of the UK Biobank Access Procedures document: https://www.ukbiobank.ac.uk/media/omtl1ie4/access-procedures-2011-1.pdf). All participants of the UK Biobank study provided consent, at the baseline visit, for their personal data and biological samples to be collected and stored for research purposes. Participants are given the option to withdraw their consent at any time; any samples that have withdrawn their consent at the time of analysis were excluded from this study. A print version of the electronic consent form is stored as UK Biobank Resource 100252.

Ethics statement FinnGen
Study subjects in FinnGen provided informed consent for biobank research, based on the Finnish Biobank Act. Alternatively, separate research cohorts, collected prior the Finnish Biobank Act came into effect (in September 2013) and start of FinnGen (August 2017), were collected based on study-specific consents and later transferred to the Finnish biobanks after approval by Fimea (Finnish Medicines Agency), the National Supervisory Authority for Welfare and Health. Recruitment protocols followed the biobank protocols approved by Fimea. The Coordinating Ethics Committee of the Hospital District of Helsinki and Uusimaa (HUS) statement number for the FinnGen study is Nr HUS/990/2017.
The FinnGen study is approved by Finnish Institute for Health and Welfare (permit numbers: THL/2031/6.02.00/2017, THL/1101/5.05.00/2017, THL/341/6.02.00/2018, THL/2222/6.02.00/2018, THL/283/6.02.00/2019, THL/1721/5.05.00/2019 and THL/1524/5.05.00/2020), Digital and population data service agency (permit numbers: VRK43431/2017-3, VRK/6909/2018-3, VRK/4415/2019-3), the Social Insurance Institution (permit numbers: KELA 58/522/2017, KELA 131/522/2018, KELA 70/522/2019, KELA 98/522/2019, KELA 134/522/2019, KELA 138/522/2019, KELA 2/522/2020, KELA 16/522/2020), Findata permit numbers THL/2364/14.02/2020, THL/4055/14.06.00/2020, THL/3433/14.06.00/2020, THL/4432/14.06/2020, THL/5189/14.06/2020, THL/5894/14.06.00/2020, THL/6619/14.06.00/2020, THL/209/14.06.00/2021, THL/688/14.06.00/2021, THL/1284/14.06.00/2021, THL/1965/14.06.00/2021, THL/5546/14.02.00/2020, THL/2658/14.06.00/2021, THL/4235/14.06.00/2021, Statistics Finland (permit numbers: TK-53-1041-17 and TK/143/07.03.00/2020 (earlier TK-53-90-20) TK/1735/07.03.00/2021, TK/3112/07.03.00/2021) and Finnish Registry for Kidney Diseases permission/extract from the meeting minutes on 4th July 2019.
The Biobank Access Decisions for FinnGen samples and data utilized in FinnGen Data Freeze 12 include: THL Biobank BB2017_55, BB2017_111, BB2018_19, BB_2018_34, BB_2018_67, BB2018_71, BB2019_7, BB2019_8, BB2019_26, BB2020_1, BB2021_65, Finnish Red Cross Blood Service Biobank 7.12.2017, Helsinki Biobank HUS/359/2017, HUS/248/2020,

HUS/430/2021 §28, §29, HUS/150/2022 §12, §13, §14, §15, §16, §17, §18, §23, §58, §59, HUS/128/2023 §18, Auria Biobank AB17-5154 and amendment #1 (August 17 2020) and amendments BB_2021-0140, BB_2021-0156 (August 26 2021, Feb 2 2022), BB_2021-0169, BB_2021-0179, BB_2021-0161, AB20-5926 and amendment #1 (April 23 2020) and it´s modifications (Sep 22 2021), BB_2022-0262, BB_2022-0256, Biobank Borealis of Northern Finland_2017_1013, 2021_5010, 2021_5010 Amendment, 2021_5018, 2021_5018 Amendment, 2021_5015, 2021_5015 Amendment, 2021_5015 Amendment_2, 2021_5023, 2021_5023 Amendment, 2021_5023 Amendment_2, 2021_5017, 2021_5017 Amendment, 2022_6001, 2022_6001 Amendment, 2022_6006 Amendment, 2022_6006 Amendment, 2022_6006 Amendment_2, BB22-0067, 2022_0262, 2022_0262 Amendment, Biobank of Eastern Finland 1186/2018 and amendment 22§/2020, 53§/2021, 13§/2022, 14§/2022, 15§/2022, 27§/2022, 28§/2022, 29§/2022, 33§/2022, 35§/2022, 36§/2022, 37§/2022, 39§/2022, 7§/2023, 32§/2023, 33§/2023, 34§/2023, 35§/2023, 36§/2023, 37§/2023, 38§/2023, 39§/2023, 40§/2023, 41§/2023, Finnish Clinical Biobank Tampere MH0004 and amendments (21.02.2020 & 06.10.2020), BB2021-0140 8§/2021, 9§/2021, §9/2022, §10/2022, §12/2022, 13§/2022, §20/2022, §21/2022, §22/2022, §23/2022, 28§/2022, 29§/2022, 30§/2022, 31§/2022, 32§/2022, 38§/2022, 40§/2022, 42§/2022, 1§/2023, Central Finland Biobank 1-2017, BB_2021-0161, BB_2021-0169, BB_2021-0179, BB_2021-0170, BB_2022-0256, BB_2022-0262, BB22-0067, Decision allowing to continue data processing until 31st Aug 2024 for projects: BB_2021-0179, BB22-0067,BB_2022-0262, BB_2021-0170, BB_2021-0164, BB_2021-0161, and BB_2021-0169, and Terveystalo Biobank STB 2018001 and amendment 25th Aug 2020, Finnish Hematological Registry and Clinical Biobank decision 18th June 2021, Arctic biobank P0844: ARC_2021_1001.

Ethics statement MGBB
The MGB Biobank has obtained a Certificate of Confidentiality. In addition, The MGB Biobank works in close collaboration with the Partners Human Research Committee (PHRC) (the Institutional Review Board). This collaboration has ensured that the Biobank's actions and procedures meet the ethical standards for research with human subjects. Biobank patients are recruited from inpatient stays, emergency department settings, outpatient visits, and electronically through a secure online portal for patients. Recruitment and consent materials are fully translated in Spanish to promote patient inclusion. The systematic enrollment of patients across the MGB network and the active inclusion of patients from diverse backgrounds contribute to a Biobank reflective of the overall demographic of the population receiving care within the MGB network. Recruitment for the Biobank launched in 2009 and is ongoing through both in-person recruitment at participating clinics and electronically through the patient portal. The recruitment strategy has been described previously40. All recruited patients provided written consent upon enrollment, and are offered an option to refuse consent.

Ethics statement MVP
The VA Office of Research and Development Central IRB approved the MVP003 and MVP028 study protocols. For all other studies with summary statistic level data the participants provided written informed consent in IRB approved protocols.

Note that full information on the approval of the study protocol must also be provided in the manuscript.

Field-specific reporting

Please select the one below that is the best fit for your research. If you are not sure, read the appropriate sections before making your selection.

[x] Life sciences   [ ] Behavioural & social sciences   [ ] Ecological, evolutionary & environmental sciences

For a reference copy of the document with all sections, see nature.com/documents/nr-reporting-summary-flat.pdf

Life sciences study design

All studies must disclose on these points even when the disclosure is negative.

Sample size	Sample sizes were chosen for each biobank where genotype and relevant phenotypic data were available.
Data exclusions	As standard practice, individuals with poor quality genotyping data were removed. In addition, individuals with dysregulated blood glucose levels were excluded.
Replication	Our original finding in the UK Biobank was replicated in the following independent cohorts: Estonian Biobank, FinnGen, MGB Biobank, and Million Veteran Program.
Randomization	Randomization was not relevant to this study. Standard and appropriate covariates were used in all analyses.
Blinding	In this retrospective epidemiological study, blinding was not relevant.

Reporting for specific materials, systems and methods

We require information from authors about some types of materials, experimental systems and methods used in many studies. Here, indicate whether each material, system or method listed is relevant to your study. If you are not sure if a list item applies to your research, read the appropriate section before selecting a response.

Materials & experimental systems

n/a	Involved in the study
☒	☐ Antibodies
☒	☐ Eukaryotic cell lines
☒	☐ Palaeontology and archaeology
☒	☐ Animals and other organisms
☒	☐ Clinical data
☒	☐ Dual use research of concern
☒	☐ Plants

Methods

n/a	Involved in the study
☒	☐ ChIP-seq
☒	☐ Flow cytometry
☒	☐ MRI-based neuroimaging

Plants

Seed stocks	NA
Novel plant genotypes	NA
Authentication	NA
