# Supplementary material for: Genetic variants affect diurnal glucose levels throughout the day
Source: Nat Commun. 2026 May 22;17:6717. doi: 10.1038/s41467-026-72432-6 (PMC13385737; doi:10.1038/s41467-026-72432-6)
Supplement: Supplementary file 5 — Transparent Peer Review File [file 41467_2026_72432_MOESM5_ESM.pdf]

nature portfolio

Peer Review File

Genetic variants affect diurnal glucose levels throughout the day

Corresponding Author: Dr Hanna Ollila

This file contains all reviewer reports in order by version, followed by all author rebuttals in order by version.

Version 0:

Reviewer comments:

Reviewer #1

(Remarks to the Author)
The paper by Ollila and the team reports intriguing findings investigating the link between genetic variants and the diurnal regulation of glucose levels. The authors should be acknowledged for their rigorous analyses and important biological reasoning. The key findings from the current study provide valuable insights into the role of circadian rhythms in glucose homeostasis, particularly the link between genetic variants in the MTNR1B and CRY2 genes and glucose regulation. Generally technical part of the analysis is well conducted. One of the key novelties of the study is the robustness of the cosinor analysis, which is well-suited for modeling rhythmic patterns in time-series data. Authors are congratulated on tremendous work and lots of important information in the manuscript, however, the conclusions could benefit from angling it towards potential implications of the results. I have a few comments to the authors that hopefully could help to improve this aspect of the findings.
-The Figure 2A and B describing SNP effects on glucose levels at different times of the day is very informative. A plot B illustrating the glucose concentrations at different times supports the physiological effects of melatonin, apart from the rise in glucose towards night that could be attributed to after dinner meal effects. Would it possible to generate similar curves of glucose excursions for the EsBB and the FinnGen studies?
-The MTNR1B variant G-allele has been proposed to potentiate melatonin signaling, thereby reducing insulin secretion, which aligns well with the current understanding of melatonin's inhibitory role in insulin release during the night-time. It is intriguing that an opposite effect is seen for the CRY2 variants. Respectively, variants in the MTNR1B gene confirm an increased risk of type 2 diabetes, while variants in the CRY2 gene associated with reduced risk of diabetes. Does it suggest that generally circadian rhythms "evening" effects on glucose excursion are less important than the "morning" effects. Variants in the MTNR1B were also reported to be associated with elevated HbA1c levels. If this is a systemic effect, it would be interesting to see whether similar diurnal patterns also influence HbA1c measurements that are used to assess long-term glucose control particularly in patients with diabetes.
-The findings that individuals who reported daytime napping or sleepiness showed no association between MTNR1B or CRY2 genetic variants and glucose levels might further support the importance of direct or indirect effect of circadian rhythms genes on other related conditions, i.e. stress phenotypes. If this is the case, it would be interesting to explore in the PheWAS analyses whether the risk of type 2 diabetes, HbA1c levels and previously reported MTNR1B-related associations with stress, anxiety and depression would be similarly diminished in the individuals with daytime napping. Such insights could have translational significance for individuals carrying the G-allele in MTNR1B.
-Variants in MTNR1B have also been associated with increased insulin resistance in several studies. The effects of MTNR1B variants on stress-related conditions, such as sleep disorders, particularly under chronic stress or circadian misalignment could provide further explanation the association between these disorders and insulin resistance.
The above-mentioned additional information would help to strengthen the manuscript and provide a comprehensive understanding and translational implications of the genetic and physiological mechanisms underlying the observed patterns of circadian regulation of glucose levels.
Minor comments:

1. Abstract: it is not clear to me what message the authors want to convey in this sentence "Furthermore, we show that sleep modulates glucose levels, and the genetic variants have a separate mechanism of diurnal control". What does separate control mean or refer to? Does it mean a mechanism independent of sleep and circadian hormones? Or the authors are looking for another missing link?
2. The similar applies to the conclusion statement. It seems that the authors aim to emphasise two key findings: 1) the

association between sleep (or diurnal rhythm perhaps?) and metabolism (represented by glucose concentrations) and 2) genetic variants modulating glucose levels independently of this relationship? It is unclear to me. If this is the intended message, it could benefit from an even more clear articulation.

Reviewer #2

(Remarks to the Author)
In “Genetic variants affect diurnal glucose levels throughout the day”, Sinnott-Armstrong et al. use data from several large population from biobanks worldwide to demonstrate that glucose levels are under diurnal control, and that in addition to sleep modulating glucose levels, specific gene SNPs (melatonin receptor 1B- MTNR1B, and CRY2) show a strong correlation with the diurnal regulation of blood glucose. The authors based their initial findings on data from the UK biobank, but verify them using large biobanks from different international sites, which is an asset of the study. Furthermore, the authors demonstrate that melatonin supplementation is associated with type II diabetes as well as earlier onset of type II diabetes. This is an important study, and the link between melatonin supplementation and diabetes risk is particularly novel and important, considering the rampant use of melatonin as a sleep aid for many individuals.

Main comments:
1. In the introduction, I think the authors should be a bit more in depth as to what the core clock is- a 24h transcriptional feedback loop that operates in almost all cell types of the body. This may provide a better context for the importance of the CRY SNP in particular. (For example, CRY2 has been shown to regulate gluconeogenesis in the liver, so the fact that mutations might contribute to alterations in glucose metabolism might not be so surprising.)
2. It would also be helpful to describe the nature of the melatonin receptor, which is a cell membrane bound G protein coupled receptor, which has tissue-specific effects. It is well known to cause vasodilation in addition to its effects on the arousal system.
3. The authors may consider mentioning in the text if there was data related to whether or not patients who had a prescription for melatonin actually took it.
4. The materials and methods outline that for various biobanks that “lifestyle data” was collected. It is not clear whether for sleep or chronotype information what specific questions or alternatively questionnaires (i.e. Munich chronotype questionnaire, morningness-eveningness questionnaire, etc.) were used. I think this would be helpful to know since sleep attributes and chronotype were also added to the model. The authors mention “morningness-eveningness” but it is not clear what data this stratification is based on.

Minor details:
1. All gene symbols should be defined at the first use (i.e. clock, bmal1, pers, crys….)
2. I find the legend for 5B slightly confusing. As I understand it, the top 3 categories refer to patients who do not have a melatonin prescription?

Reviewer #3

(Remarks to the Author)
This large-scale genomic study investigates how common genetic variants influence diurnal variation in blood glucose levels. Using UK Biobank data (~420,000 individuals) and replication in several major biobanks (FinnGen, MVP, Estonian Biobank, and MGB Biobank), the authors identify time-dependent associations at two key circadian loci, MTNR1B and CRY2. These variants show opposing effects on glucose levels depending on the time of day and are independently associated with type 2 diabetes risk. The work employs cosinor models, generalized additive models, and heterogeneity analyses to dissect time-varying effects. This manuscript presents clear novelty, robust methodology, and important implications for circadian medicine and metabolic research. Addressing a few interpretative and methodological clarifications would further enhance its clarity and translational impact.

While the use of cosinor models is appropriate for modeling circadian effects, they assume a fixed 24-hour rhythm and a symmetric sinusoidal waveform, which may not accurately capture individual variability or asymmetric patterns in glucose rhythms. In addition, as the UK Biobank mostly collected blood samples in the morning, the cosinor fitting curve may be biased. It would be helpful to report the model’s goodness-of-fit statistics.

Although the authors performed within-individual comparisons of morning and evening glucose values in 22,456 individuals from FinnGen, it remains unclear whether these paired measurements were taken on the same day. Given the nature of EHR data, it is likely that the values were collected on different days, which may introduce additional variability and limit the precision of diurnal effect estimation.

The study has made commendable efforts to account for fasting status by stratifying analyses by fasting duration and sample collection time. However, the impact of glucose-lowering medications or diabetes treatment was not uniformly adjusted across cohorts, which may influence the observed associations.

Version 1:

Reviewer comments:

Reviewer #1

(Remarks to the Author)
The authors have satisfactory addressed all queries. I have only comment related to the title of the manuscript. It is noteworthy to indicate that this work specifically addresses the genetic variants in the MTNR1B and CRY2 genes.

The authors should be congratulated on this large amount of interesting work. I have no further comments.

Reviewer #2

(Remarks to the Author)
The authors have adequately and thoroughly addressed all of my initial concerns.

Reviewer #3

(Remarks to the Author)
Thanks for addressing the comments. I have no further questions.

Open Access This Peer Review File is licensed under a Creative Commons Attribution 4.0 International License, which permits use, sharing, adaptation, distribution and reproduction in any medium or format, as long as you give appropriate credit to the original author(s) and the source, provide a link to the Creative Commons license, and indicate if changes were made.
In cases where reviewers are anonymous, credit should be given to 'Anonymous Referee' and the source.
The images or other third party material in this Peer Review File are included in the article's Creative Commons license, unless indicated otherwise in a credit line to the material. If material is not included in the article's Creative Commons license and your intended use is not permitted by statutory regulation or exceeds the permitted use, you will need to obtain permission directly from the copyright holder.
To view a copy of this license, visit https://creativecommons.org/licenses/by/4.0/

REVIEWER COMMENTS

Reviewer #1 (Remarks to the Author):

The paper by Ollila and the team reports intriguing findings investigating the link between genetic variants and the diurnal regulation of glucose levels. The authors should be acknowledged for their rigorous analyses and important biological reasoning. The key findings from the current study provide valuable insights into the role of circadian rhythms in glucose homeostasis, particularly the link between genetic variants in the MTNR1B and CRY2 genes and glucose regulation. Generally technical part of the analysis is well conducted. One of the key novelties of the study is the robustness of the cosinor analysis, which is well-suited for modeling rhythmic patterns in time-series data. Authors are congratulated on tremendous work and lots of important information in the manuscript, however, the conclusions could benefit from angling it towards potential implications of the results. I have a few comments to the authors that hopefully could help to improve this aspect of the findings.

Question 1.1 The Figure 2A and B describing SNP effects on glucose levels at different times of the day is very informative. A plot B illustrating the glucose concentrations at different times supports the physiological effects of melatonin, apart from the rise in glucose towards night that could be attributed to after dinner meal effects. Would it possible to generate similar curves of glucose excursions for the EsBB and the FinnGen studies?

Response 1.1 This is an excellent suggestion, and we now provide the glucose curves for the EstBB and FinnGen studies (Figure S9). The curves clearly show that glucose levels recapitulate the higher SNP effect in the morning and the lower effect in the evening. In both cohorts, the midday curve shows a smaller glucose or food intake related increase at lunchtime, which likely reflects differences in hospital-based sample collection in EstBB and FinnGen compared to the invited patient selection in UKB.

Question 1.2 The MTNR1B variant G-allele has been proposed to potentiate melatonin signaling, thereby reducing insulin secretion, which aligns well with the current understanding of melatonin’s inhibitory role in insulin release during the night-time. It is intriguing that an opposite effect is seen for the CRY2 variants. Respectively, variants in the MTNR1B gene confirm an increased risk of type 2 diabetes, while variants in the CRY2 gene associated with reduced risk of diabetes. Does it suggest that generally circadian rhythms “evening” effects on glucose excursion are less important than the “morning” effects.

Variants in the MTNR1B were also reported to be associated with elevated HbA1c levels. If this is a systemic effect, it would be interesting to see whether similar diurnal patterns also influence HbA1c measurements that are used to assess long-term glucose control particularly in patients with diabetes.

Response 1.2 The question about evening versus morning effects is interesting. While evening and morning effects differ, these could be explained also by the difference by primarily circadian (homeostatic) effects of the variants on glucose levels whereas evening associations likely reflect post-prandial or environmental response to glucose intake.
To understand and formally test the role of genetic variants on diurnal long-term elevated glucose and HbA1c we examined the effect of the *MTNR1B* rs10840963 variant on HbA1c levels in the UK Biobank sample, and found that while there is an effect of the allele on HbA1c, its effect was consistent throughout the day (**Fig. S13**). Considering that HbA1c levels are reflective of long-term elevated blood sugar, however, we do not necessarily expect a similar diurnal pattern as the rs10830963 has on blood glucose levels. These findings are now included in the manuscript.

Question 1.3 The findings that individuals who reported daytime napping or sleepiness showed no association between MTNR1B or CRY2 genetic variants and glucose levels might further support the importance of direct or indirect effect of circadian rhythms genes on other related conditions, i.e. stress phenotypes. If this is the case, it would be interesting to explore in the PheWAS analyses whether the risk of type 2 diabetes, HbA1c levels and previously reported MTNR1B-related associations with stress, anxiety and depression would be similarly diminished in the individuals with daytime napping. Such insights could have translational significance for individuals carrying the G-allele in MTNR1B.

Response 1.3. We have examined the variant effects on T2DM, HbA1c, anxiety and depression, stratified by napping. We did not observe association with major depressive disorder or with anxiety. Instead, we found that the effect of the MTNR1B G allele (rs10830963) was significant on HbA1c and type 2 diabetes, but that the effects were consistent for individuals who never, sometimes, or often napped or slept during the day. The lack of association of rs10830963 with HbA1c and type 2 diabetes in individuals who often napped is likely due to lower power (n=11,541-12,117) compared to individuals who reported sometimes (n=89,594-93,964) or never/rarely napping during the day (n=334,085-350,512) (Figure 1).

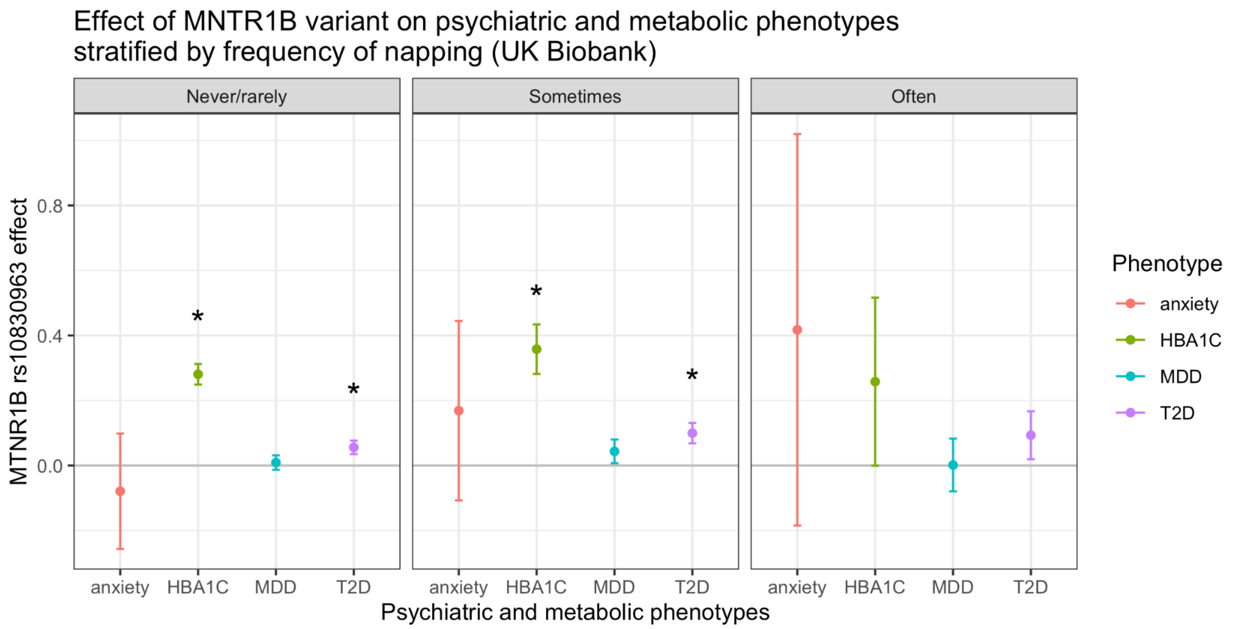

Figure 1 Effect of MTNR1B G allele (rs10830963) on psychiatric and metabolic phenotypes. T2D = type 2 diabetes, MDD = major depressive disorder. * indicates adjusted p value < 0.05.

Question 1.4 Variants in MTNR1B have also been associated with increased insulin resistance in several studies. The effects of MTNR1B variants on stress-related conditions, such as sleep disorders, particularly under chronic stress or circadian misalignment could provide further explanation the association between these disorders and insulin resistance. The above-mentioned additional information would help to strengthen the manuscript and provide a comprehensive understanding and translational implications of the genetic and physiological mechanisms underlying the observed patterns of circadian regulation of glucose levels.

Response 1.4 It is possible that chronic stress influences glucose levels and diurnal variation. However, in our analyses stratified by napping, sleep traits, anxiety, and depression, as well as in the broader PheWAS, we did not observe evidence of single-variant associations that would support a stress-related interaction.

In contrast, gene-level PheWAS results from the Sleep Disorder Knowledge Portal showed significant associations at the *MTNR1B* and *CRY2* loci with psychiatric and sleep traits, suggesting a broader genomic link between these regions and sleep-related biology.

The role of sleep is further supported by previous chronotype GWAS findings, where *MTNR1B* variants (rs10830963 and rs4237555, r2 = 0.32; Figure S14) showed independent associations with chronotype. While we did not find a genome-wide significant signal at *CRY2* in our data, region-wide PheWAS results indicate an association with chronotype that is independent of glucose-related effects (*CRY2*

rs34239319 and rs12419690, r2 = 0.008). The glucose lead variant itself shows a nominal association with sleep traits (Figure S14b).

We plan to examine the connection between glucose, sleep and the role of genetic risk factors in future work using continuous glucose monitoring and accelerometer devices in order to have comprehensive data at individual level both at sleep and glucose levels.

Minor comments:

1. Abstract: it is not clear to me what message the authors want to convey in this sentence "Furthermore, we show that sleep modulates glucose levels, and the genetic variants have a separate mechanism of diurnal control". What does separate control mean or refer to? Does it mean a mechanism independent of sleep and circadian hormones? Or the authors are looking for another missing link?

Minor 1.1. We have clarified this sentence stating that the genetic variants have an effect on diurnal glucose levels.

2. The similar applies to the conclusion statement. It seems that the authors aim to emphasise two key findings: 1) the association between sleep (or diurnal rhythm perhaps?) and metabolism (represented by glucose concentrations) and 2) genetic variants modulating glucose levels independently of this relationship? It is unclear to me. If this is the intended message, it could benefit from an even more clear articulation.

Minor 1.2. We have also clarified the conclusion.

Reviewer #2 (Remarks to the Author):

In "Genetic variants affect diurnal glucose levels throughout the day", Sinnott-Armstrong et al. use data from several large population from biobanks worldwide to demonstrate that glucose levels are under diurnal control, and that in addition to sleep modulating glucose levels, specific gene SNPs (melatonin receptor 1B- MTNR1B, and CRY2) show a strong correlation with the diurnal regulation of blood glucose. The authors based their initial findings on data from the UK biobank, but verify them using large biobanks from different international sites, which is an asset of the study. Furthermore, the authors demonstrate that melatonin supplementation is associated with type II diabetes as well as earlier onset of type II diabetes. This is an important study, and the link between melatonin supplementation and diabetes risk is particularly novel and important, considering the rampant use of melatonin as a sleep aid for many individuals.

Main comments:

Question 2.1. In the introduction, I think the authors should be a bit more in depth as to what the core clock is- a 24h transcriptional feedback loop that operates in almost all cell types of the body. This may provide a better context for the importance of the CRY SNP in particular. (For example, CRY2 has been shown to regulate gluconeogenesis in the liver, so the fact that mutations might contribute to alterations in glucosemetabolism might not be so surprising.)

Response 2.1 This is a great suggestion and we have included description how the clock operates in every cell in the body as following:

Circadian rhythms not only orchestrate the timing of wake and sleep but also regulate homeostasis within the body. Circadian rhythms are controlled by core circadian genes CLOCK and BMAL1. CLOCK and BMAL drive the expression of their repressors PER and CRY genes and create a ~24-hour oscillation of gene activity1,2. This molecular clock is present in virtually all cells, with the central pacemaker located in the suprachiasmatic nucleus of the hypothalamus. The circadian system integrates external cues such as light, temperature, feeding, and activity to synchronize internal homeostasis and physiology with the environment. Circadian rhythms also significantly contribute to physiological processes, including diurnal variation in homeostasis and gene expression. In addition to sleep-wake regulation, circadian rhythms contribute to the regulation of metabolism, affecting nutrient sensing, energy expenditure, glucose and lipid homeostasis, and hormone release. Furthermore, the core clock genes directly connect with metabolism. Glucose metabolism is affected by gluconeogenesis that is coordinated by the CRY genes. In addition, CLOCK and BMAL activate downstream effector genes like REV-ERBa directly at central and peripheral tissues affecting diurnal control of metabolism. Finally, disruption of circadian genes in model organisms manifests with altered glucose metabolism and diabetes.

Question 2.2. It would also be helpful to describe the nature of the melatonin receptor, which is a cell membrane bound G protein coupled receptor, which has tissue-specific effects. It is well known to cause vasodilation in addition to its effects on the arousal system.

Response 2.2 This is an excellent point. We have updated the manuscript and provide a description of the G protein coupled receptor system and their relevance as pharmaceutical targets.

MTNR1B is a G protein-coupled receptor (GPCR). Overall, GPCRs constitute the largest class of membrane receptors and the central role of GPCRs in human biology is

highlighted by the fact that 36% of FDA-approved drugs act on GPCRs. GPCRs have a wide tissue distribution and can have tissue specific regulatory effects. Such effects are also accentuated with MTNR1B including the effects in the central nervous system in sleep and circadian rhythms, role in glucose metabolism in the pancreas and effects on temperature regulation and cardiovascular system through vasodilation. This underscores both the clinical importance and the vast biological and clinical effects of GPCRs including MTNR1B which is already used as a pharmaceutical target.

Question 2.3 The authors may consider mentioning in the text if there was data related to whether or not patients who had a prescription for melatonin actually took it.

Response 2.3 In the current data we record prescriptions and purchases only but not compliance with drug use. We have included this point in the limitations section.

Question 2.4 The materials and methods outline that for various biobanks that "lifestyle data" was collected. It is not clear whether for sleep or chronotype information what specific questions or alternatively questionnaires (i.e. Munich chronotype questionnaire, morningness-eveningness questionnaire, etc.) were used. I think this would be helpful to know since sleep attributes and chronotype were also added to the model. The authors mention "morningness-eveningness" but it is not clear what data this stratification is based on.

Response 2.4 This is an excellent point. All UK Biobank questions were assessed with a single question and the larger questionnaires were not implemented. To improve the clarity and readability of the Materials and methods section we provide the exact questions used in the UK Biobank baseline questionnaire.

Minor details:
Minor Q2.1. All gene symbols should be defined at the first use (i.e. clock, bmal1, pers, crys….).

Minor R2.1. Many gene acronyms are derived from non-informative original gene names. We are including the current acronyms only in the manuscript for readability.

Minor Q2.2. I find the legend for 5B slightly confusing. As I understand it, the top 3 categories refer to patients who do not have a melatonin prescription?

Minor R2.2 This is correct. In the figure we have individuals who develop T2DM without melatonin and individuals with melatonin. The individuals who have melatonin prescription have earlier disease onset compared to those who do not have melatonin prescription.

Reviewer #3 (Remarks to the Author):

This large-scale genomic study investigates how common genetic variants influence diurnal variation in blood glucose levels. Using UK Biobank data (~420,000 individuals) and replication in several major biobanks (FinnGen, MVP, Estonian Biobank, and MGB Biobank), the authors identify time-dependent associations at two key circadian loci, MTNR1B and CRY2. These variants show opposing effects on glucose levels depending on the time of day and are independently associated with type 2 diabetes risk. The work employs cosinor models, generalized additive models, and heterogeneity analyses to dissect time-varying effects. This manuscript presents clear novelty, robust methodology, and important implications for circadian medicine and metabolic research. Addressing a few interpretative and methodological clarifications would further enhance its clarity and translational impact.

Question 3.1 While the use of cosinor models is appropriate for modeling circadian effects, they assume a fixed 24-hour rhythm and a symmetric sinusoidal waveform, which may not accurately capture individual variability or asymmetric patterns in glucoserhythms. In addition, as the UK Biobank mostly collected blood samples in the morning, the cosinor fitting curve may be biased. It would be helpful to report the model's goodness-of-fit statistics.

Response 3.1 This is an excellent suggestion. We provide goodness of fit statistics for the cosinor model. We found that while the r^2 of the model was not substantial (sine(time) r^2 =0.0098, cosine(time) r^2 = 0.010), there was a significant difference between models including cosinor time and a null model containing only covariates (sine(time) p value = 2.026x10-12, cosine(time) p value = $2.96x10^{-34}$). We provide the goodness of fit statistics in the supplementary data.

These results show that while blood glucose levels are affected by a myriad of environmental factors, circadian time is still a significant contributor to the measured levels. While cosinor models may not perfectly model the relationship of blood glucose measurements to time of day, particularly in non-fasting glucose measurements, our subsequent analysis of heterogeneity of effect sizes in the MTNR1B and CRY2 loci throughout the day support a picture of genetic variants that interact with time to affect blood glucose levels differentially throughout the day.

Question 3.2 Although the authors performed within-individual comparisons of morning and evening glucose values in 22,456 individuals from FinnGen, it remains unclear whether these paired measurements were taken on the same day. Given the nature of EHR data, it is likely that the values were collected on different days, which may introduce additional variability and limit the precision of diurnal effect estimation.

Response 3.2 This is an important point and there is likely additional variability caused when the estimates are not from the same day. In our paired dataset, only 15.5% of the paired values were taken within 24 hours of one another. When we examined the difference in morning-evening glucose values in this small subset of individuals (n=3,497), we found a consistent effect size for both *MTNR1B* and *CRY2* risk variants, but were underpowered to see a significant association *MTNR1B* rs10830963 beta = -0.0347, p=0.627, *CRY2* rs12419690 beta = 0.0427, p=0.547). This finding suggests that a more systematic measurement of glucose over time of day, such as with continuous glucose monitoring, would allow us to better understand such diurnal metabolic traits.

Question 3.3 The study has made commendable efforts to account for fasting status by stratifying analyses by fasting duration and sample collection time. However, the impact of glucose-lowering medications or diabetes treatment was not uniformly adjusted across cohorts, which may influence the observed associations.

Response 3.3 We agree with this point, and have edited the limitations section. We now write that:

"While the cohorts have extensive medical information, the available data types such glucose-lowering medications or diabetes treatment was not available at similar resolution at all cohorts. This may influence the observed associations in the study."
